# Phenolate‐Induced N−O Bond Formation versus TiemannType Rearrangement for the Synthesis of 3‐Aminobenzisoxazoles and 2‐Aminobenzoxazoles

**DOI:** 10.1002/open.202200252

**Published:** 2022-12-23

**Authors:** Benedikt Hufnagel, W. Felix Zhu, Hanna M. Franz, Ewgenij Proschak, Victor Hernandez‐Olmos

**Affiliations:** ^1^ Fraunhofer Institute for Translational Medicine and Pharmacology ITMP Theodor-Stern-Kai 7 60596 Frankfurt am Main Germany; ^2^ Institute of Pharmaceutical Chemistry Goethe University Frankfurt Max-von-Laue-Str. 9 60438 Frankfurt am Main Germany

**Keywords:** 3-aminobenzisoxazoles, 2-aminobenzoxazoles, nitrenoid precursors, oxadiazolone, Tiemann rearrangement

## Abstract

A novel oxadiazolone‐based method for the synthesis of 3‐aminobenzisoxazoles by N−O bond formation and of 2‐aminobenzoxazoles through a Tiemann‐type rearrangement has been developed. The synthesis of these two pharmaceutically relevant heterocycles was realized by an unexplored retrosynthetic disconnection using a cyclic nitrenoid precursor‐based strategy. The selective formation of the two isomers was significantly influenced by steric and electronic effects of substituents. However, tetrabutylammonium chloride (TBACl) efficiently promoted the Tiemann‐type rearrangement over N−O bond formation. Control experiments indicate that deprotonation of the phenol induces both rearrangements.

## Graphical abstract

**Both the first intramolecular N−O bond formation** and the competitive Tiemann‐type rearrangement via oxadiazolones as cyclic nitrenoid precursors were developed. Selectivity for the two isomers was remarkably influenced by steric and electronic effects, but tetrabutylammonium chloride (TBACl) was discovered as an effective additive to promote the Tiemann‐type rearrangement over N−O bond formation.

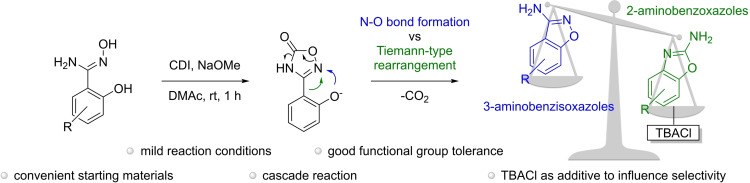

## Introduction

**Figure 1:**
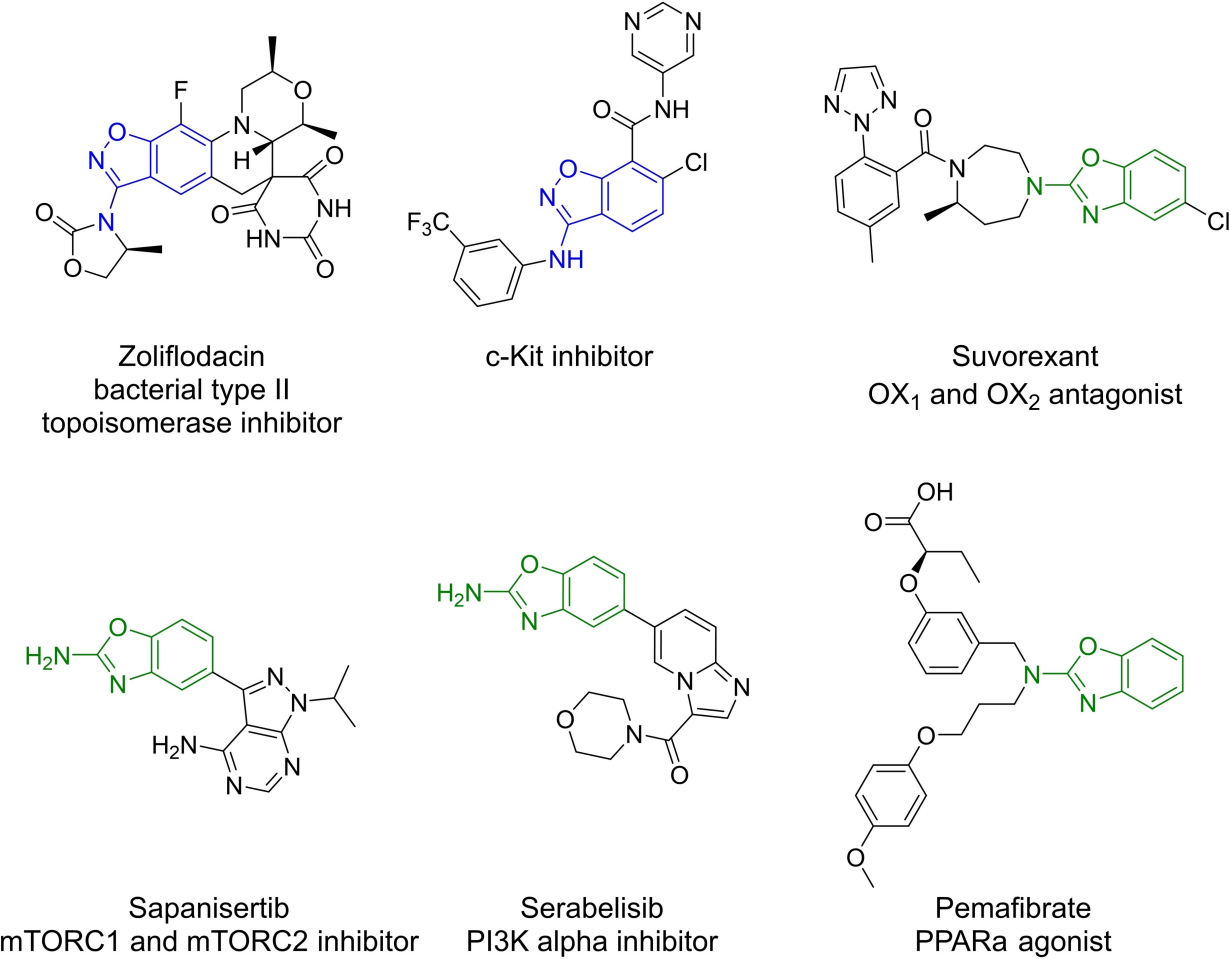
Approved, investigational new or experimental drugs, which contain 3aminobenzisoxazole (dark blue) or 2aminobenzoxazole (green) scaffold.

3‐Aminobenzisoxazoles and 2‐aminobenzoxazoles are both important building blocks in drug discovery, as they occur in numerous biologically active molecules. This is underlined by several approved, investigational new, or experimental drugs, which contain these heterocycles (Figure 1).^[1]^

**Scheme 1:**
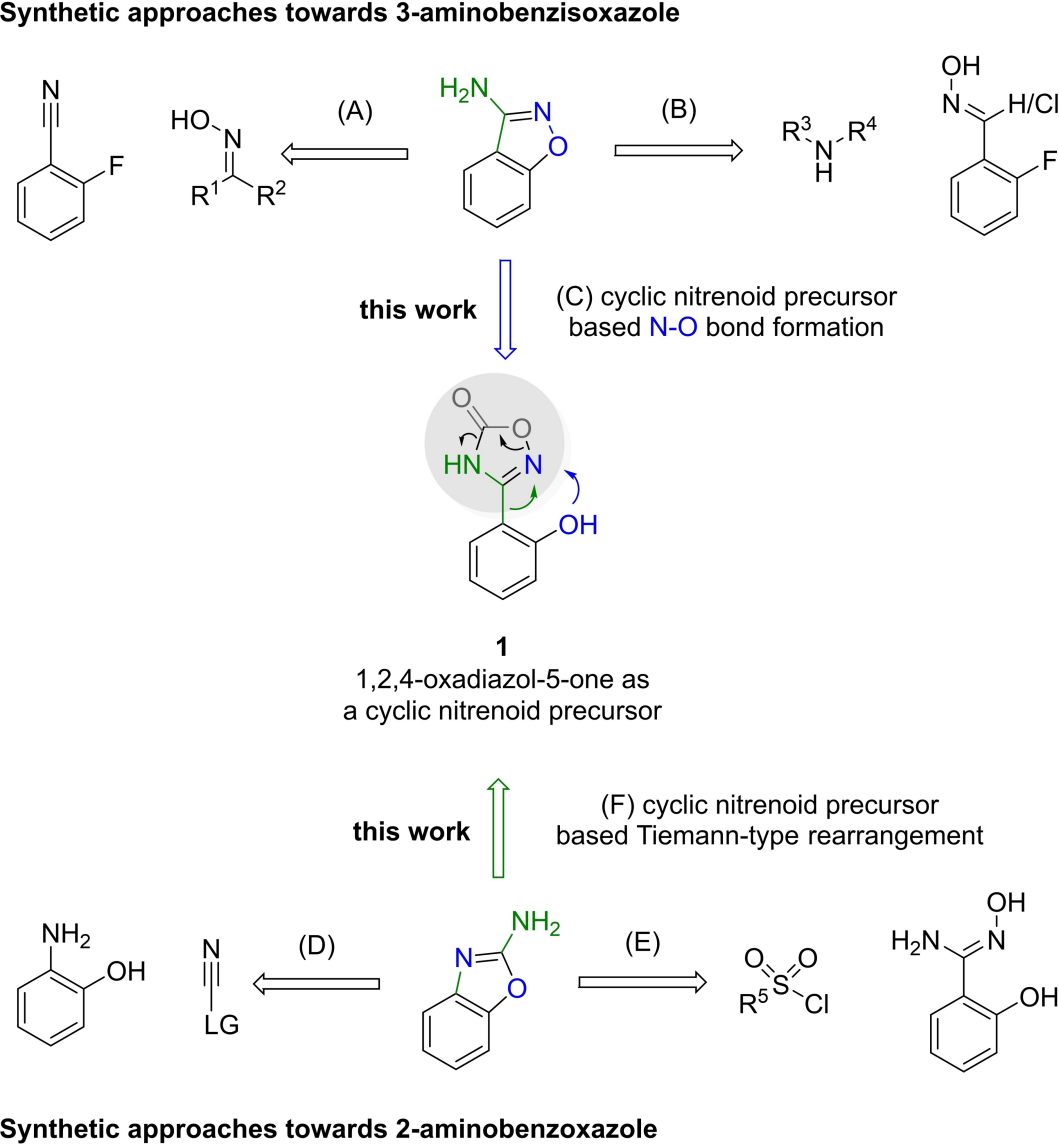
Synthetic approaches towards 3‐aminobenzisoxazole and 2*‐*aminobenzoxazole.

Although various protocols for their synthesis exist, development of novel strategies with unexplored retrosynthetic disconnections could complement them regarding functional group tolerance, efficiency, and employable reactants (Scheme 1). 3‐Aminobenzisoxazoles are usually prepared by an S_N_Ar reaction of 2‐fluorobenzonitriles with oximes, followed by hydrolysis of the oxime and intramolecular condensation (A).^[2]^ Alternatively, 2‐fluorobenzaldehydes form aldoximes with hydroxylamines (B). Then, chlorination of the resulting aldoxime is followed by nucleophilic displacement by amines. Finally, cyclization through an S_N_Ar reaction provides the desired 3‐amino‐substituted benzisoxazoles.^[3]^ Both strategies are limited to electron‐poor aromatic rings because they rely on an S_N_Ar reaction in one of the steps.

In all of the existing methods, the N−O bond originates from hydroxylamine but the retrosynthetic N−O bond disconnection for the synthesis of 3‐aminobenzisoxazoles has not been established yet. This approach would allow the use of more electronically diverse aromatic rings. One possibility to enable 3‐aminobenzisoxazole synthesis via N−O bond formation is the coupling of a hydroxy group in *ortho* position with an electrophilic imidoyl nitrene (C). Nitrenes are highly reactive species and generated in situ from suitable precursors. Cyclic nitrenoid precursors are an emerging class of nitrene transfer reagents, which can be activated by temperature, light, transition metals, or combinations thereof.^[4]^ One representative subclass of them, namely 1,2,4‐oxadiazol‐5(4*H*)‐ones, have been described as precursor to the required imidoyl nitrene.^[4]^ In this work, we envisioned that 3‐(2‐hydroxyphenyl)‐1,2,4oxadiazol‐5(4*H*)‐one (**1**) could therefore lead to the synthesis of 3‐aminobenzisoxazoles via N−O bond formation and CO_2_‐elimination.

2‐Aminobenzoxazoles are synthesized from 2‐aminophenol derivatives, which react with an electrophilic cyanating reagent (D). Cyanogen bromide is an effective but very hazardous reagent for this purpose.^[5]^ Continuous‐flow adaptation^[6]^ and development of alternative cyanogen sources (e. g., isothiocyanates,^[7]^ *N*‐cyanobenzotriazole,^[8]^ di(imidazole‐1‐yl‐methanimine^[9]^ and *N*‐cyano‐*N*‐phenyl‐*p*‐toluenesulfonamide^[10]^) have led to safer synthetic procedures. Furthermore, C−H amination,^[11]^ C−X (X=halide) amination^[12]^ and other reactions^[13]^ have also been reported to obtain 2‐aminobenzoxazoles. Another possibility is the Tiemann rearrangement with a hydroxyl group in *ortho* position, where *O*‐sulfonylation of amidoximes induces N−O bond cleavage and aryl migration to afford cyanamides.[^14^, ^15^, ^16^, ^17^] Since 1,2,4‐oxadiazol‐5(4*H*)‐ones also possess activatable N−O bonds, a Tiemann‐type rearrangement might occur analogous to Curtius‐type rearrangements, which are known to take place upon activation of 1,4,2‐dioxazol‐5‐ones.^[18]^ Therefore, **1** could also facilitate the synthesis of 2‐aminobenzoxazoles via a Tiemann‐type rearrangement (F).

In this study, we present our results about both the first intramolecular N−O bond formation for the synthesis of 3‐aminobenzisoxazoles and the competitive Tiemann‐type rearrangement for the synthesis of 2‐aminobenzoxazoles via cyclic nitrenoid precursors.

## Results and Discussion

Recently, we have disclosed the intramolecular N−N bond formation of 1,2,4‐oxadiazol‐5(4*H*)‐ones with secondary amines in *ortho* position (corresponding to Scheme 1C).^[19]^ Cyclization takes place under thermal conditions and the Tiemann‐type rearrangement product was never observed. Using amidoxime **2 a** as the model substrate, we investigated in this study whether an analogous N−O bond formation would afford 3‐aminobenzisoxazole (**3 a**) (Table 1). Contrary to our previous findings, the reaction was faster, as it was already completed within 1 h at rt, affording **3 a** in 14 % yield. Additionally, Tiemann‐type rearrangement product **4 a** was also identified even with a higher yield (entry 1). This was unexpected, because usually heat, photocatalysis, or transition metal catalysis is required for the activation of cyclic nitrenoid precursors. Evaluation of bases revealed their strong influence on both selectivity and yield, whereby NaOMe afforded the best results in favor of **3 a** (entries 2‐6). Varying solvents, equivalents of base, reagents, concentration, or temperature from entry 6 did not improve yields. Performing the reaction under inert atmosphere finally led to the highest absolute yield of **3 a** accompanied by quantitative reaction conversion considering the two isomers.

**Table 1:** Optimization of reaction conditions.^[a]^

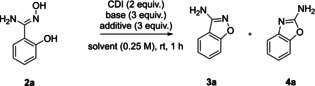					
Entry	Solvent	Base/additive	**3 a** [%]^[b]^	**4 a** [%]^[b]^	Selectivity **3 a**/**4 a**
1	DMAc	Cs_2_CO_3_	14	25	0.56
2	DMAc	K_3_PO_4_	27	31	0.87
3	DMAc	KO*t*Bu	45	12	3.75
4	DMAc	NaO*t*Bu	57	19	3.00
5	DMAc	NaH	58	–	–
6	DMAc/DMAc^[c]^	NaOMe	63/73	15/27	5.25/2.70
7	DMAc	K_3_PO_4_/TBACl	9	46	0.19
8	DMAc^[c]^	NaOMe/TBACl	7	93	0.08

[a] 1 equiv.=0.5 mmol, Atmospheric conditions unless otherwise noted. MAc=dimethylacetamide. [b] Isolated yield. [c] Argon atmosphere.

During the optimization, we found that addition of tetrabutylammonium chloride (TBACl) shifted the selectivity towards the other isomer (entry 2 vs. entry 7). Based on this observation, we tried to find another set of reaction conditions, which are optimized for the synthesis of **4 a**. In the end, the highest yield of **4 a** was again obtained by the combination of dimethylacetamide (DMAc) and NaOMe but with addition of TBACl (entry 8). Thereby, selectivity was completely reversed solely by the additive, while quantitative total yield of both isomers was retained (entry 8 vs. entry 6).

Based on the finding that TBACl influenced the selectivity of the reaction, the role of further additives was examined (Table 2). Similar yields and selectivities were obtained with other tetrabutylammonium salts indicating that the counter anion is not responsible for the additive effect (entries 3‐6 compared to entries 1 and 2). TBAI was an exception, affording a lower total yield but still favoring the formation of **4 a** (entry 7). Instead, it was suspected that coordination of the tetrabutylammonium cation to the phenolate anion was responsible for the favored Tiemann‐type rearrangement (see below for a discussion of the reaction mechanism). This was supported by employment of different chloride salts, where NaCl and KCl did not influence the isomer ratio (entries 9‐10 compared to entry 7). NH_4_Cl displayed some additive effect albeit with a lower efficiency than TBACl (entry 11 compared to entry 8). Taken together, these results suggest that tetrabutylammonium cation effectively promotes the Tiemann‐type rearrangement over N−O bond formation.

**Table 2:** Influence of additives on selectivity.^[a]^

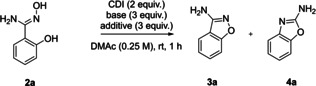					
Entry	Solvent	Base/additive	**3 a** [%]^[b]^	**4 a** [%]^[b]^	Selectivity **3 a**/**4 a**
1	DMAc	K_3_PO_4_	27	31	0.87
2	DMAc	K_3_PO_4_/TBACl	9	46	0.19
3	DMAc	K_3_PO_4_/TBAF⋅H_2_O	–	42	–
4	DMAc	K_3_PO_4_/TBABr	12	45	0.27
5	DMAc	K_3_PO_4_/TBAI	4	13	0.31
6	DMAc	K_3_PO_4_/TBA[HSO_4_]	3	38	0.08
7	DMAc^[c]^	NaOMe	73	27	2.70
8	DMAc^[c]^	NaOMe/TBACl	7	93	0.08
9	DMAc^[c]^	NaOMe/NaCl^[d]^	28	13	2.15
10	DMAc^[c]^	NaOMe/KCl^[d]^	38	12	2.92
11	DMAc^[c]^	NaOMe/NH_4_Cl	30	34	0.88

[a] 1 equiv.=0.5 mmol, Atmospheric conditions unless otherwise noted. [b] Isolated yield. [c] Argon atmosphere. [d] 0.125 m solution in DMAc.

**Scheme 2:**
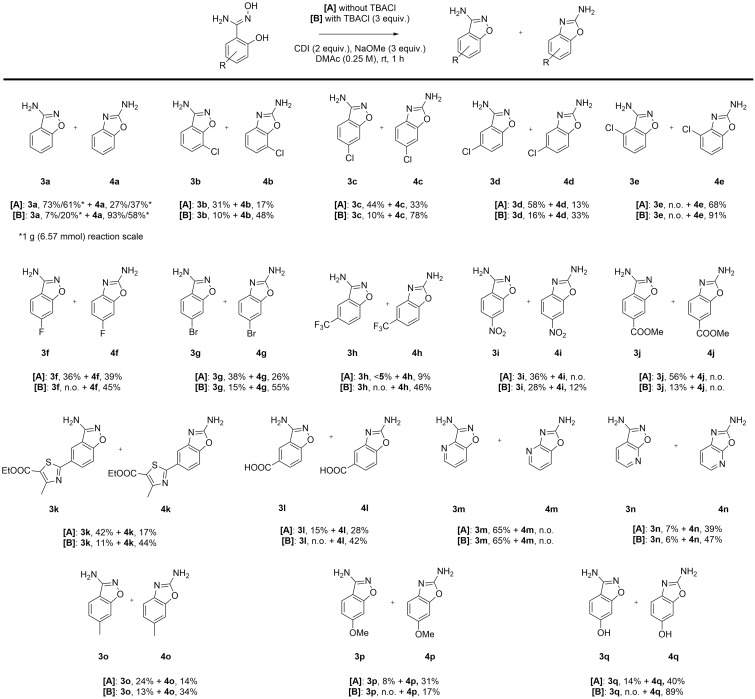
Scope of reaction with substituted amidoximes. Reactions were usually carried out on a 0.5 mmol reaction scale. n.o.=not obtained.

Using the optimized conditions for each isomer, we studied the scope of the reaction with different substituents and substitution patterns of the phenyl ring (Scheme 2). It should be noted that the required amidoximes can be conveniently synthesized from the corresponding nitriles.^[20]^ Diverse substituents, including halides (**3 b**–**h**+**4 b**–**h**), nitro group (**3 i**+**4 i**), esters (**3 j‐k**+**4 j**–**k**) and carboxylic acid group (**3 l**+**4 l**) were tolerated under moderate to good total yields. Furthermore, N‐heterocyclic amidoximes revealed a strong preference for different isomers (**3 m**–**n**+**4 m**–**n**). Electron‐rich derivatives could also be synthesized (**3 o**–**q**+**4 o**–**q**). Addition of TBACl increased the yield of the respective 2‐aminobenzoxazole in all cases except for **3 j**+**4 j** and **3 p**+**4 p** proving its effectiveness in promoting the Tiemann‐type rearrangement. However, it became evident that both electronic and steric effects exhibited a remarkable influence on the selectivity of this reaction, which primarily determined the ratio of each set of isomers. Interestingly, comparison of differently chloro‐substituted derivatives (**3 b**–**e**+**4 b**–**e**) revealed that steric hindrance in *ortho* position to the amidoxime inhibits N−O bond formation completely. Unfortunately, we were unsuccessful in synthesizing the amidoxime with an *ortho*‐substituted methyl group, which would have allowed for comparison of different electronic effects in this position. Very electron‐poor substituents (**3 i**–**j**+**4 i**–**j**) in *para* position disfavored formation of the respective 2‐aminobenzoxazole. We assumed that this was because substituents that withdraw electron density from the aromatic carbon attached to the oxadiazolone (hereinafter referred to as ‘C^1^’), discourage aryl migration and therefore the Tiemann‐type rearrangement. This was further supported by electron‐poor derivative **3 l**+**4 l**. Here, Tiemann‐type rearrangement was the major product formation pathway because the carboxylic acid is in *meta* position to the oxadiazolone, and electron density is withdrawn from the phenolate anion rather than from C^1^. The previously mentioned selectivity for N‐heterocyclic derivatives can be explained by the electronic influence on either C^1^ (**3 m**+**4 m**) or the phenolate anion (**3 n**+**4 n**) as well. In the first case, the inductive effect of the nitrogen disfavors aryl migration, while in the second case, the decreased nucleophilicity of the phenolate anion impedes N−O bond formation. Lastly, the difference in selectivity among the electron‐rich derivatives **3 o**–**q**+**4 o**–**q** can also be explained by the mesomeric effect of the methoxy and hydroxy substituents in comparison to the inductive effect of the methyl group. Repeating the reaction on a gram scale afforded slightly lower yields and selectivity (**3 a**+**4 a**).

**Scheme 3:**
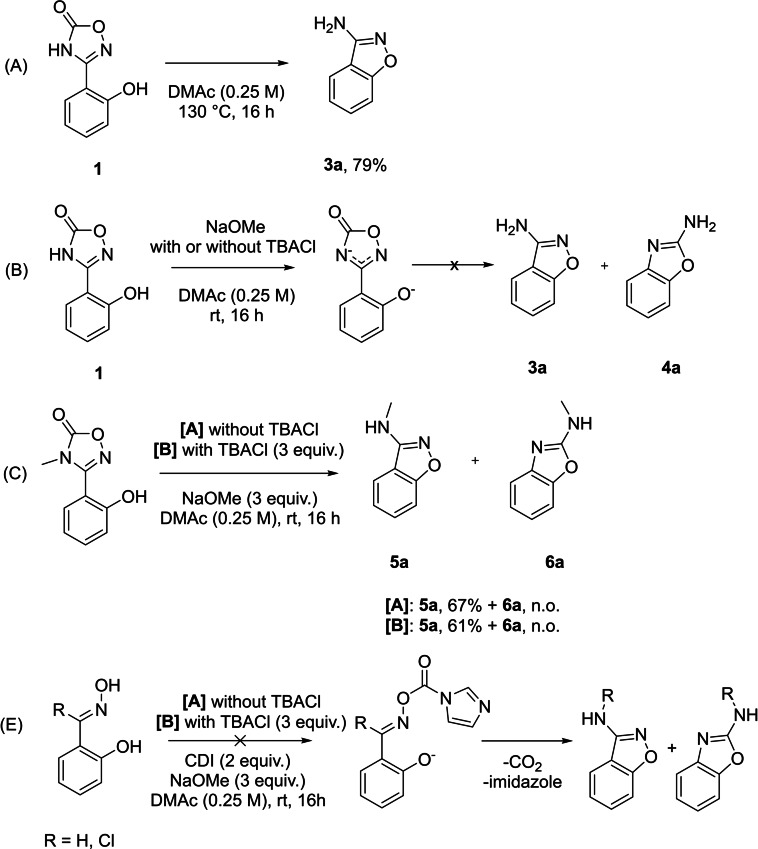
Control experiments: (A) Thermal rearrangement of **1** affords the N−O bond formation product. (B) Deprotonation of oxadiazolone deactivates reaction. (C) Methylated oxadiazolone undergoes N−O bond formation. (D) Aldoxime and chlorinated analogue undergo neither N−O bond formation nor Tiemann‐type rearrangement.

The investigation of the substrate scope revealed how steric hindrance and electronic effects impacted the two competing reactions. Further control experiments were conducted to gain additional insights about the reaction mechanisms (Scheme 3). We aimed to find an explanation for the high reactivity of both reactions and the competitiveness of the Tiemann‐type rearrangement since the corresponding N−N bond formation requires higher temperatures and 3‐aminoindazoles are the only products.[^19^, ^21^] Therefore, we isolated the putative intermediate **1** and investigated its phenolate‐induced rearrangements. We hypothesized that N−O bond formation should be facilitated by the increased nucleophilicity of the hydroxy group upon deprotonation. At the same time, the positive mesomeric effect of the phenolate anion is stronger compared to phenol or aniline, which might be the reason why the Tiemann‐type rearrangement is able to compete. If both reactions would only proceed at these mild conditions through phenolate‐induced activation, protonated compound **1** should be less reactive and possible to isolate. Indeed, the synthesis of putative intermediate **1** was successful under acidic conditions (Scheme S1, Supporting Information). Without presence of base, **1** was stable in solution at room temperature. Only N−O bond formation occurred upon heating, which is in agreement with the N−N bond formation regarding reaction kinetics and chemoselectivity (A).[^19^, ^21^] Then, deprotonation of **1** was induced with or without TBACl (B). Following the idea of phenolate‐induced activation, conversion of **1** to both **3 a** and **4 a** was monitored. However, no reaction was observed upon treatment with base even after a prolonged reaction time. We attributed this to the deprotonation of not only the phenol, but the oxadiazolone as well. N−H groups of oxadiazolones are acidic and increased electron density resulting from electron pair delocalization presumably deactivates both aryl migration and N−O bond formation.^[22]^ This hypothesis was examined by performing the same control experiment with the methylated oxadiazolone (C). In that case, N−O bond formation occurred in comparable yield, proving that deprotonation of the oxadiazolone ring indeed deactivates this reaction. The Tiemann‐type rearrangement product was, however, not observed even with addition of TBACl. An alternative reaction mechanism would consist of the actual Tiemann rearrangement with 1,1′‐carbonyldiimidazole (CDI) for activation instead of sulfonylation (E). Reaction would occur before cyclization to the oxadiazolone under extrusion of CO_2_. Aldoxime and its chlorinated analogue were used as model substrates, as they cannot cyclize to a nitrenoid precursor. In both cases, neither N−O bond formation nor Tiemann rearrangement was observed, indicating that this mechanistic pathway is not viable under these reaction conditions.

**Scheme 4:**
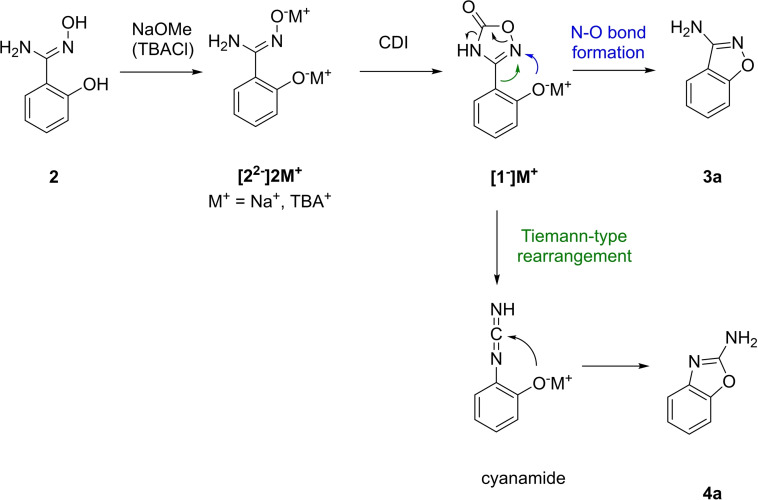
Proposed mechanism for cyclic nitrenoid‐based N−O bond formation and for the Tiemann‐type rearrangement.

Combining these results, we propose the following reaction mechanism (Scheme 4). Although the Tiemann‐type rearrangement was not observed with the methylated oxadiazolone, it is most likely that both reactions occur via the cyclic nitrenoid precursor as the intermediate. This is supported by various analogous rearrangements with related heterocycles in combination with the experiments from Scheme 3F.[^18^, ^23^] First, sufficient phenol deprotonation before cyclization has proven crucial for reaction outcome (**[2^2−^]2 M^+^**). Furthermore, TBA coordinates as counter cation if employed as an additive to promote the Tiemann‐type rearrangement (M=TBA^+^, otherwise M=Na^+^). Stabilization of the phenolate anion through coordination of tetrabutylammonium cation likely decreases the tendency to undergo N−O bond formation. Both N−O bond formation and the Tiemann‐type rearrangement must occur before deprotonation of oxadiazolone (**[1^−^]M^+^**). Driving forces of intramolecular N−O bond formation are decarboxylation and aromative annulation, which afford **3 a**.[^19^, ^21^] Alternatively, aryl migration leads to formation of cyanamide equivalent to the Tiemann rearrangement.[^14^, ^15^, ^16^] Subsequent nucleophilic attack by phenolate anion yields **4 a**.^[17]^

## Conclusion

The oxadiazolone‐based synthesis of both 3aminobenzisoxazoles and 2‐aminobenzoxazoles has been developed. This enables the use of commercially available 2‐hydroxybenzonitriles as convenient starting materials. Starting from 3‐(2‐hydroxyphenyl)‐1,2,4‐oxadiazol‐5(4*H*)‐one (**1**), this study provides not only the first example of a nitrenoid precursor‐based, intramolecular N−O bond formation, but also for a nitrenoid precursor‐based aryl migration that affords 2‐aminobenzoxazoles. Substrate scope studies revealed remarkable steric and electronic effects on the selectivity of the two competing reactions. While these primarily defined the selectivity of the two isomers, TBACl was discovered as an effective, safe, and affordable additive, which promotes the Tiemann‐type rearrangement. Insights about the two reactions were gained through several control experiments, including their phenolate‐induced activation, which is responsible for the mild reaction conditions and short reaction time. In conclusion, this study represents another successful application of 1,2,4oxadiazol‐5(4*H*)ones as valuable synthetic precursors in an emerging research field of organic synthesis.

## Experimental Section

**General information**: Reactions were carried out under argon atmosphere using an argon filled balloon. NMR spectra were recorded with Brucker Avance DPX250, Bruker Avance 300, Bruker Avance 400, or Bruker Avance 500 spectrometers all from Bruker operating at ambient temperature. Proton spectra were recorded in DMSO‐d_6_ and ^1^H NMR chemical shifts were referenced to the residual signal of DMSO‐d_6_ (at δ=2.50 ppm). ^13^C{1H} NMR chemical shifts were referenced against the central line of DMSO‐d_6_ at δ=39.52 ppm. ^19^F NMR chemical shifts were referenced to external CFCl_3_ (0.0 ppm). Chemical shifts are given on δ scale (ppm). Coupling constants (*J*) are given in Hz. Multiplicities are indicated as follows: s (singlet), d (doublet), t (triplet), q (quartet), quint or m (multiplet). ESI‐MS were measured with LCMS‐2020 from Shimadzu and HRMS with MALDI Orbitrap XL from Thermo Scientific. TLC was carried out on silica gel plates from Marcherey‐Nagel (ALUGRAM®) and visualized with an UV lamp (254 nm and/or 366 nm). Purification of products was performed by flash chromatography using puriFlash XS420. Silica HP 30 μm columns or C18‐HP 30 μm columns were employed as stationary phases, all from Interchim. *n*‐Hexane/EtOAc or DCM/MeOH were used as eluents for normal phase column chromatography and H_2_O/MeOH was used as eluent for reversed phase column chromatography. Semipreparative HPLC was conducted by SHIMADZU prominence with a SPD20 A UV/Vis detector from Shimadzu. Stationary phase was Luna 10u C18(2) (250 ⋅ 21.20 mm) from Phenomenex and the eluent was a mixture of ACN and aq. formic acid solution (0.1 %). Flow rate was set to 21 mL min^−1^.

**General procedure for the synthesis of** ***N*** **′‐hydroxybenzimidamides**: The respective 2‐hydroxybenzonitrile (1.0 equiv.) was dissolved in EtOH (0.2 m). Hydroxylamine hydrochloride (2.0 equiv.) and triethylamine (2.0 equiv.), or alternatively, an aq. hydroxylamine solution (50 wt%, 2.0 equiv.) was added. The reaction mixture was heated to reflux in an oil bath overnight. Once the reaction mixture cooled down to rt, it was concentrated under reduced pressure, diluted with EtOAc, washed 2×with an aq. saturated NaHCO_3_ solution and 1×with an aq. saturated NaCl solution. The organic phase was dried over MgSO_4_ and concentrated under reduced pressure. Purification was performed by flash chromatography on silica gel. *N*′‐Hydroxybenzimidamides were usually unstable under HPLC conditions and purity was ensured by NMR analysis.

**General procedure for the synthesis of 3‐aminobenzisoxazoles and 2‐aminobenzoxazoles**: The respective *N′*‐hydroxybenzimidamide (1.0 equiv.) was dissolved in DMAc (0.25 m). TBACl (3.0 equiv.) was added, if 2‐aminobenzoxazole was the preferred product, followed by NaOMe (3.0 equiv.) and the reaction mixture was stirred for 10 min at rt. Then, CDI (2.0 equiv.) was added, and the reaction mixture was stirred for another 1 h at rt. The reaction mixture was neutralized with aq. saturated NH_4_Cl solution and diluted with EE (50×V_DMAc_). The organic phase was washed 2×with aq. saturated NaHCO_3_ solution, 1×with an aq. saturated NaCl solution, dried over MgSO_4_, and concentrated under reduced pressure. Purification was performed by flash chromatography on silica gel.

## Conflict of interest

The authors declare no conflict of interest.

1

## Supporting information

As a service to our authors and readers, this journal provides supporting information supplied by the authors. Such materials are peer reviewed and may be re‐organized for online delivery, but are not copy‐edited or typeset. Technical support issues arising from supporting information (other than missing files) should be addressed to the authors.

Supporting Information

## Data Availability

The data that support the findings of this study are available in the supplementary material of this article.
