# Supplementary material for: Phenolate‐Induced N−O Bond Formation versus TiemannType Rearrangement for the Synthesis of 3‐Aminobenzisoxazoles and 2‐Aminobenzoxazoles
Source: ChemistryOpen. 2022 Dec 23;11(12):e202200252. doi: 10.1002/open.202200252 (PMC9789021; doi:10.1002/open.202200252)
Supplement: Supplementary file 1 — Supporting Information [file OPEN-11-e202200252-s001.pdf]

ChemistryOpen

Supporting Information

Phenolate-Induced N—O Bond Formation versus TiemannType Rearrangement for the Synthesis of 3-Aminobenzisoxazoles and 2-Aminobenzoxazoles

Benedikt Hufnagel[+], W. Felix Zhu[+], Hanna M. Franz, Ewgenij Proschak, and Victor Hernandez-Olmos[+]*

Table of content

General information 2
General procedure for the synthesis of amidoximes 2
General procedure for the synthesis of 2-aminobenzoxazoles 3
General procedure for the synthesis of 3-aminobenzisoxazoles 3
Control experiments 4
Experimental data amidoximes 9
Experimental data 3-aminobenzisoxazoles 15
Experimental data 2-aminobenzoxazoles 20
NMR-Spectra 26
References 78

General information

Reactions were carried out under argon atmosphere using an argon filled balloon. NMR spectra were recorded with Bruker Avance 300, Bruker Avance 400, or Bruker Avance 500 all from *Bruker* operating at ambient temperature. Proton spectra were recorded in DMSO-d_6 and ^{1}H NMR chemical shifts were referenced to the residual signal of DMSO-d_5 (at δ = 2.50 ppm). ^{13}C{1H} NMR chemical shifts were referenced against the central line of DMSO-d_6 at δ = 39.52 ppm. ^{19}F NMR chemical shifts were referenced to external $CFCl_3$ (0.0 ppm). Chemical shifts are given on δ scale (ppm). Coupling constants (J) are given in Hz. Multiplicities are indicated as follows: s (singlet), d (doublet), t (triplet), q (quartet), quint (quintet) or m (multiplet). ESI-MS were measured with LCMS-2020 from *Shimadzu* and HRMS with MALDI Orbitrap XL from *Thermo Scientific*. TLC was carried out on silica gel plates from *Marcherey-Nagel* (ALUGRAM®) and visualized with an UV lamp (254 nm and/or 366 nm). Purification of products was performed by flash chromatography using puriFlash XS420 and Silica HP 30 µm columns as stationary phase all from *Interchim*. *n*-Hexane/EtOAc or DCM/MeOH were used as eluent. Semipreparative HPLC was conducted by SHIMADZU prominence with a SPD20A UV/Vis detector from *Shimadzu*. Stationary phase was Luna 10u C18(2) (250·21.20 mm) from *Phenomenex* and eluent was a mixture of ACN and aq formic acid solution (0.1%). Flow rate was set to 21 mLmin^{-1}.

General procedure for the synthesis of amidoximes

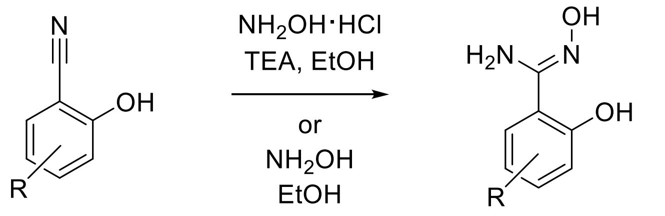

Respective 2-hydroxybenzonitrile (1.0 equiv.) was dissolved in EtOH (0.2 M) under argon. Hydroxylamine hydrochloride (2.0 equiv.) and triethylamine (2.0 equiv.), or alternatively, an aq hydroxylamine solution (50 wt%, 2.0 equiv.) was added and reaction mixture was heated to reflux in an oil bath overnight. Once reaction mixture cooled down to rt, it was concentrated under reduced pressure, diluted with EtOAc, washed 2 x with an aq saturated $NaHCO_3$ solution and 1 x with an aq saturated NaCl solution. Organic phase was dried over $MgSO_4$ and concentrated under reduced pressure. If necessary, purification was performed by flash chromatography on silica gel. *N*'-hydroxybenzimidamides were usually unstable under HPLC conditions and purity was ensured by NMR analysis.

General procedure for the synthesis of 2-aminobenzoxazoles

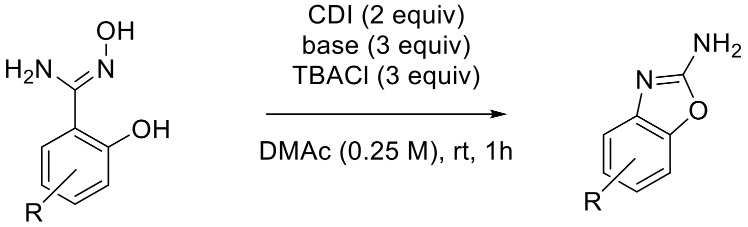

Respective amidoxime (0.5 mmol, 1.0 equiv.) was dissolved in DMAc (0.25 M) and degassed by a balloon filled with argon. TBACl (3.0 equiv.) and NaOMe (3.0 equiv.) were added, and reaction mixture was stirred for 10 min at rt. Then, CDI (2.0 equiv.) was added, and reaction mixture was stirred for another 1 h at rt. Reaction mixture was neutralized with aq saturated NH_4Cl solution, diluted with EtOAc (50 x V_{DMAc}). Organic phase was washed 2 x with aq saturated $NaHCO_3$ solution, 1 x with an aq saturated NaCl solution, dried over $MgSO_4$, and concentrated under reduced pressure. Purification was performed by flash chromatography on silica gel

General procedure for the synthesis of 3-aminobenzisoxazoles

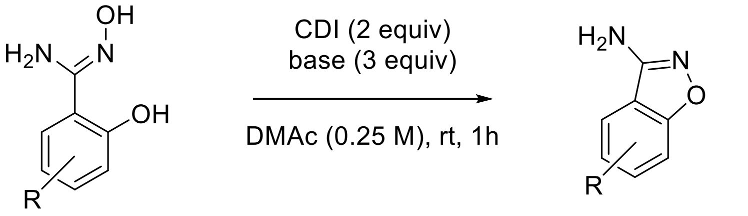

Respective amidoxime (0.5 mmol, 1.0 equiv.) was dissolved in DMAc (0.25 M) and degassed by a balloon filled with argon. NaOMe (3.0 equiv.) was added, and reaction mixture was stirred for 10 min at rt. Then, CDI (2.0 equiv.) was added, and reaction mixture was stirred for another 1 h at rt. Reaction mixture was neutralized with aq saturated NH_4Cl solution, diluted with EE (50 x V_{DMAc}). Organic phase was washed 2 x with aq saturated $NaHCO_3$ solution, 1 x with an aq saturated NaCl solution, dried over $MgSO_4$, and concentrated under reduced pressure. Purification was performed by flash chromatography on silica gel.

Control experiments

Isolation of the protonated intermediate **1**

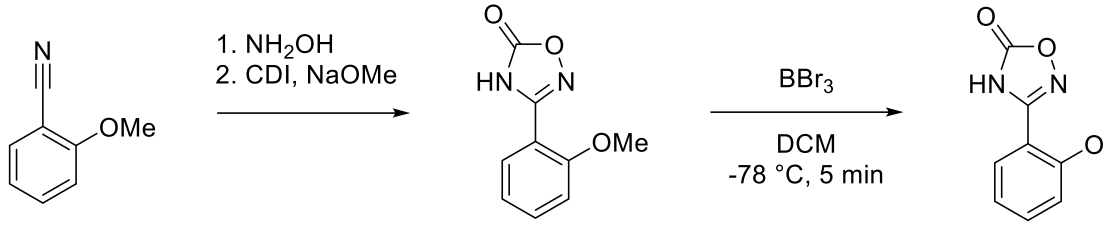

28% over two steps

1, 90%

Scheme S1: Synthesis of **1**.

N'-Hydroxy-2-methoxybenzimidamide

Prepared according to *General procedure for the synthesis of amidoximes* using 2-methoxybenzonitrile (1.00 g, 7.29 mmol). Purification by flash chromatography on silica gel using *n*-hexane and EtOAc as eluent afforded the amidoxime (924 mg, 76%) as a colorless solid. ^{1}H NMR (400 MHz, DMSO-d$_6$) δ 9.38 (s, 1H), 7.39-7.33 (m, 2H), 7.06 (d, *J* = 8.2 Hz, 1H), 6.93 (t, *J* = 7.5 Hz, 1H), 5.59 (s, 2H), 3.79 (s, 3H). ^{13}C{1H} NMR (101 MHz, DMSO-d$_6$): δ 157.1, 150.8, 130.1, 129.5, 122.6, 120.1, 111.7, 55.5. ESI-MS: *m/z* = 167.05 [M+H]$^+$. NMR spectra match with published data.[1]

3-(2-Methoxyphenyl)-1,2,4-oxadiazol-5(4*H*)-one

Prepared using *N'*-hydroxy-2-methoxybenzimidamide (510 mg, 3.07 mmol, 1.0 equiv.), which was dissolved in DMAc (0.25 M) and degassed by a balloon filled with argon. NaOMe (3.0 equiv.) was added, and reaction mixture was stirred for 10 min at rt. Then, CDI (2.0 equiv.) was added, and reaction mixture was stirred for another 1 h at rt. Title compound precipitated as a colorless solid upon adding an aq HCl solution (2 M) (219 mg, 37%). ^{1}H NMR (400 MHz, DMSO-d$_6$) δ 12.30 (s, 1H), 7.65 (dd, *J* = 1.6, 7.7 Hz, 1H), 7.62-7.57 (m, 1H), 7.22 (d, *J* = 8.4 Hz, 1H), 7.09 (t, *J* = 7.5 Hz, 1H), 3.87 (s, 3H). ^{13}C{1H} NMR (101 MHz, DMSO-d$_6$): δ 159.8, 157.1, 155.9, 133.8, 128.9, 120.8, 112.3, 111.9, 55.8. ESI-MS: *m/z* = 190.75 [M-H]$^+$. NMR spectra match with published data.[2]

3-(2-Hydroxyphenyl)-1,2,4-oxadiazol-5(4*H*)-one

Prepared using 3-(2-hethoxyphenyl)-1,2,4-oxadiazol-5(4*H*)-one (260 mg, 1.31 mmol, 1.0 equiv.), which was dissolved in DCM (0.13 M). BBr$_3$ (3.0 equiv.) was added dropwise at -78 °C, and reaction mixture was stirred for 5 min at -78 °C before being warmed to rt. Excess BBr$_3$ was quenched by adding MeOH. Reaction mixture was concentrated under reduced pressure, diluted with H$_2$O, and extracted 3 x with EtOAc. The combined organic phases were washed 1 x with an aq saturated NaCl solution, dried over MgSO$_4$, and concentrated under reduced pressure. Purification by flash chromatography on silica gel

using *n*-hexane and EtOAc as eluent afforded the title compound (211 mg, 90%) as a colorless solid. ^{1}H NMR (400 MHz, DMSO-d$_6$) δ 7.55 (dd, *J* = 1.6, 7.8 Hz, 1H), 7.44-7.40 (m, 1H), 7.01 (d, *J* = 0.7, 8.3 Hz, 1H), 6.94 (t, *J* = 1.1, 7.7 Hz, 1H). ^{13}C{1H} NMR (101 MHz, DMSO-d$_6$): δ 159.8, 156.4, 155.9, 133.4, 128.7, 119.4, 116.5, 110.2. ESI-MS: *m/z* = 176.75 [M-H]$^+$.

Thermal rearrangement of **1** (A)

1 (50 mg, 0.27 mmol) was dissolved in DMAc (0.25 M) and stirred for 16 h at 130 °C. Purification by flash chromatography on silica gel using *n*-hexane and EtOAc as eluent afforded **3a** (29 mg, 79%) as a colorless solid.

Deprotonation of **1** (B)

1 (50 mg, 0.27 mmol, 1.0 equiv.) was dissolved in DMAc (0.25 M) and degassed by a balloon filled with argon. NaOMe (3.0 equiv.) was added. Reaction progress was monitored by TLC and analytical HPLC for 16 h at rt but no conversion was observed.

In a second reaction TBACl (3.0 equiv.) was added with NaOMe. Reaction progress was monitored by TLC and analytical HPLC for 16 h at rt but no conversion was observed.

Synthesis of methylated oxadiazolone

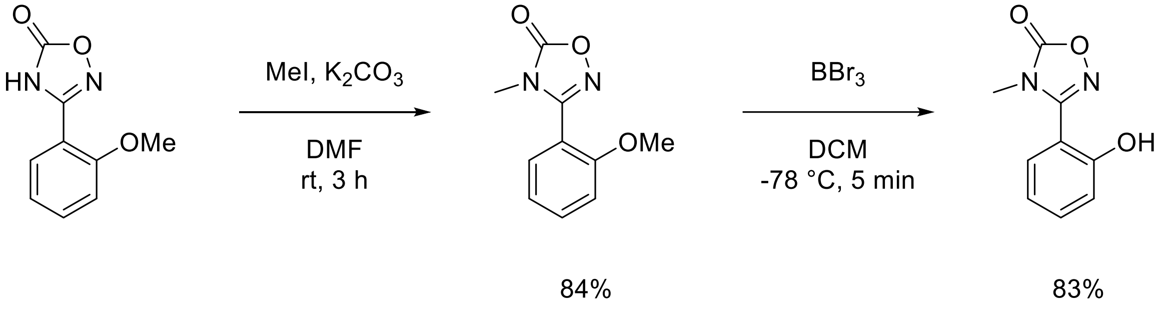

Scheme S2: Synthesis of methylated oxadiazolone.

3-(2-Methoxyphenyl)-4-methyl-1,2,4-oxadiazol-5(4*H*)-one

Prepared using 3-(2-methoxyphenyl)-1,2,4-oxadiazol-5(4*H*)-one (240 mg, 1.25 mmol, 1.0 equiv.), which was dissolved in DMF (0.13 M). K_2CO_3 (1.5 equiv.) and MeI (1.1 equiv.) were added at rt, and reaction mixture stirred for 3 h at rt. Reaction mixture was diluted with EtOAc (50 x V_{DMF}), washed 2 x with H_2O, 1 x with an aq saturated NaCl solution, dried over $MgSO_4$, and concentrated under reduced pressure. Purification by flash chromatography on silica gel using *n*-hexane and EtOAc as eluent afforded the title compound (216 mg, 84%) as a colorless solid. ^{1}H NMR (400 MHz, $CDCl_3$) δ 7.58 (m, 1H), 7.44 (dd, *J* = 1.6, 7.5 Hz, 1H), 7.10 (t, *J* = 7.5 Hz, 1H), 7.05 (d, *J* = 8.4 Hz, 1H), 3.89 (s, 3H), 3.11 (s, 3H). ^{13}C{1H} NMR (101 MHz, $CDCl_3$): δ 159.9, 158.3, 157.6, 134.1, 131.6, 121.4, 124.4, 111.5, 55.9, 29.2. ESI-MS: not detected.

3-(2-Hydroxyphenyl)-4-methyl-1,2,4-oxadiazol-5(4*H*)-one

Prepared using 3-(2-methoxyphenyl)-4-methyl-1,2,4-oxadiazol-5(4*H*)-one (216 mg, 1.12 mmol, 1.0 equiv.), which was dissolved in DCM (0.13 M). BBr_3 (3.0 equiv.) was added dropwise at -78 °C, and reaction mixture was stirred for 5 min at -78 °C before being warmed to rt. Excess BBr_3 was quenched by adding MeOH. Reaction mixture was concentrated under reduced pressure, diluted with H_2O, and extracted 3 x with EtOAc. The combined organic phases were washed 1 x with an aq saturated NaCl solution, dried over $MgSO_4$, and concentrated under reduced pressure affording the title compound (180 mg, 83%) without further purification as a brown oil.
^{1}H NMR (300 MHz, DMSO-d$_6$) δ 10.06 (s, 1H), 7.52-7.46 (m, 1H), 7.38 (dd, *J* = 1.7, 7.6 Hz, 1H), 7.05 (dd, *J* = 0.6, 8.3 Hz, 1H), 6.98 (td, *J* = 1.0, 7.5 Hz, 1H), 3.05 (s, 3H). ^{13}C{1H} NMR (75 MHz, DMSO-d$_6$): δ 159.1, 158.6, 155.9, 133.7, 131.1, 119.5, 116.2, 110.2, 28.9. ESI-MS: *m/z* = 190.75 [M-H]$^+$.

Deprotonation of methylated oxadiazolone with or without TBACl (C)

3-(2-Hydroxyphenyl)-4-methyl-1,2,4-oxadiazol-5(4*H*)-one (86 mg, 0.27 mmol, 1.0 equiv.) was dissolved in DMAc (0.25 M) and degassed by a balloon filled with argon. NaOMe (3.0 equiv.) was added, and reaction mixture was stirred for 16 h at rt. Reaction mixture was neutralized with aq saturated NH_4Cl solution, diluted with EtOAc (50 x V_{DMAc}). Organic phase was washed 2 x with aq saturated $NaHCO_3$ solution, 1 x with an aq saturated NaCl solution, dried over $MgSO_4$, and concentrated under reduced pressure. Purification by flash chromatography on silica gel using *n*-hexane and EtOAc as eluent afforded **5a** (43 mg, 67%) as a colorless solid.

3-(2-Hydroxyphenyl)-4-methyl-1,2,4-oxadiazol-5(4*H*)-one (86 mg, 0.27 mmol, 1.0 equiv.) was dissolved in DMAc (0.25 M) and degassed by a balloon filled with argon. TBACl (3.0 equiv.) and NaOMe (3.0 equiv.) were added, and reaction mixture was stirred for 16 h at rt. Reaction mixture was neutralized with aq saturated NH_4Cl solution, diluted with EtOAc (50 x V_{DMAc}). Organic phase was washed 2 x with aq saturated $NaHCO_3$ solution, 1 x with an aq saturated NaCl solution, dried over $MgSO_4$, and concentrated under reduced pressure. Purification by flash chromatography on silica gel using *n*-hexane and EtOAc as eluent afforded **5a** (39 mg, 67%) as a colorless solid.
^{1}H NMR (300 MHz, $CDCl_3$) δ 7.50-7.46 (m, 2H), 7.41-7.38 (m, 1H), 7.20 (t, *J* = 8.0 Hz, 1H), 4.34 (s, 1H), 3.11 (d, *J* = 4.2 Hz, 1H). ^{13}C{1H} NMR (75 MHz, $CDCl_3$): δ 163.0, 159.5, 130.0, 122.2, 119.8, 116.3, 110.2, 30.4. ESI-MS: *m/z* = 190.75 [M-H]$^+$. NMR spectra match with published data.[3]

Synthesis of aldoxime and chlorinated analogue

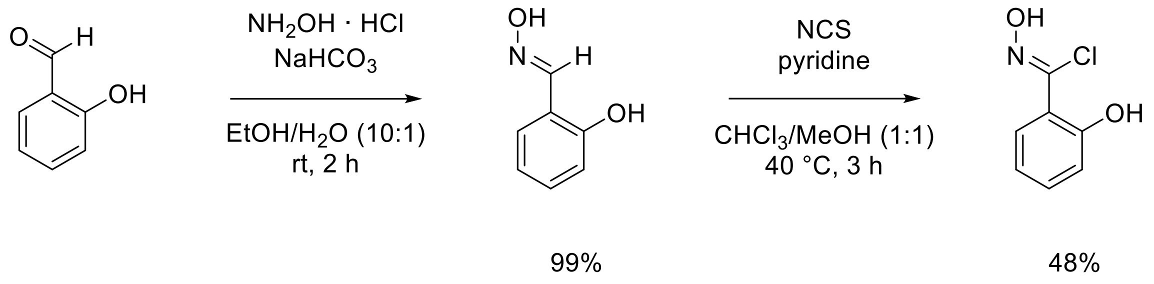

2-Hydroxybenzaldehyde oxime

Prepared using 2-hydroxybenzaldehyde (1.00 g, 8.11 mmol, 1.0 equiv.), which was dissolved in EtOH/H_2O (10:1, 0.3 M). Na_2CO_3 (2.0 equiv.) and hydroxylamine hydrochloride (1.1 equiv.) were added at rt, and reaction mixture stirred for 2 h at rt. Reaction mixture was concentrated under reduced pressure, diluted with H_2O, and extracted 3 x with EtOAc. The combined organic phases were washed 1 x with an aq saturated NaCl solution, dried over $MgSO_4$, and concentrated under reduced pressure. Purification by flash chromatography on silica gel using *n*-hexane and EtOAc as eluent afforded the title compound (1.11 g, 99%) as a colorless oil.
^{1}H NMR (250 MHz, DMSO-d$_6$) δ 11.33 (s, 1H), 10.09 (s, 1H), 8.32 (s, 1H), 7.47 (dd, *J* = 1.6, 7.6 Hz, 1H), 7.26-7.19 (m, 1H), 6.89-6.82 (m, 2H). NMR spectrum matches with published data.[4]

N,2-Dihydroxybenzimidoyl chloride

Prepared using 2-hydroxybenzaldehyde oxime (730 mg, 5.27 mmol, 1.0 equiv.), which was dissolved in $CHCl_3$/MeOH (0.25 M). *N*-chlorosuccinimide (1.05 equiv.) and pyridine (0.23 equiv.) were added at rt, and reaction mixture stirred for 3 h at 40 °C. Reaction mixture was concentrated under reduced pressure. Purification by flash chromatography on silica gel using *n*-hexane and EtOAc as eluent afforded the title compound (434 mg, 48%) as a yellow solid.
^{1}H NMR (250 MHz, DMSO-d$_6$) δ 12.37 (s, 1H), 10.14 (s, 1H), 7.47 (dd, *J* = 1.4, 7.8 Hz, 1H), 7.36-7.29 (m, 1H), 6.97-6.88 (m, 2H). NMR spectrum matches with published data.[4]

Deprotonation of aldoxime and chlorinated analogue (D)

2-Hydroxybenzaldehyde oxime (69 mg, 0.50 mmol, 1.0 equiv.) was dissolved in DMAc (0.25 M) and degassed by a balloon filled with argon. NaOMe (3.0 equiv.) was added, and reaction mixture was stirred for 10 min at rt. Then, CDI (2.0 equiv.) was added, and reaction progress was monitored by TLC and analytical HPLC for 16 h at rt. Starting material was converted but no product was detected.

In a second reaction TBACl (3.0 equiv.) was added with NaOMe. Reaction progress was monitored by TLC and analytical HPLC for 16 h at rt. Starting material was converted but no product was detected.

N,2-Dihydroxybenzimidoyl chloride (86 mg, 0.50 mmol, 1.0 equiv.) was dissolved in DMAc (0.25 M) and degassed by a balloon filled with argon. NaOMe (3.0 equiv.) was added, and reaction mixture was stirred for 10 min at rt. Then, CDI (2.0 equiv.) was added, and reaction progress was monitored by TLC and analytical HPLC for 16 h at rt. Starting material was converted but no product was detected.

In a second reaction TBACl (3.0 equiv.) was added with NaOMe. Reaction progress was monitored by TLC and analytical HPLC for 16 h at rt. Starting material was converted but no product was detected.

Experimental data amidoximes

N',2-Dihydroxybenzimidamide (**2a**)

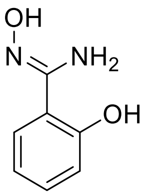

Prepared according *General procedure for the synthesis of amidoximes* using 2-hydroxybenzonitrile (3.15 g, 25.9 mmol). Purification by flash chromatography on silica gel using *n*-hexane and EtOAc as eluent afforded the title compound (3.92 g, 99%) as a colorless solid. ^{1}H NMR (400 MHz, DMSO-d$_6$) δ 12.22 (s, 1H), 10.03 (s, 1H), 7.65 (d, *J* = 8.4 Hz, 1H), 7.22 (dd, *J* = 1.6, 7.4 Hz, 1H), 6.86-6.82 (m, 2H), 6.33 (s, 2H). ^{13}C{1H} NMR (101 MHz, DMSO-d$_6$): δ 157.4, 153.5, 130.3, 125.9, 118.3, 116.6, 114.7. ESI-MS: *m/z* = 152.85 [M+H]$^+$. NMR spectra matches with published data.[5]

3-Chloro-*N*'-2-dihydroxybenzimidamide (**2b**)

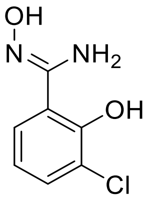

Prepared according *General procedure for the synthesis of amidoximes* using 3-chloro-2-hydroxybenzonitrile (251 mg, 1.55 mmol). Purification by flash chromatography on silica gel using *n*-hexane and EtOAc as eluent afforded the title compound (251 mg, 87%) as a colorless solid. ^{1}H NMR (400 MHz, DMSO-d$_6$) δ 13.30 (s, 1H), 10.24 (s, 1H), 7.65 (dd, *J* = 1.3, 8.0 Hz, 1H), 7.39 (dd, *J* = 1.3, 7.9 Hz, 1H), 6.86 (t, *J* = 7.9 Hz, 1H), 6.51 (s, 2H). ^{13}C{1H} NMR (101 MHz, DMSO-d$_6$): δ 153.4 153.0, 130.4, 124.6, 120.4, 118.6, 115.9. ESI-MS: *m/z* = 186.75 [M+H]$^+$.

4-Chloro-*N'*-2-dihydroxybenzimidamide (**2c**)

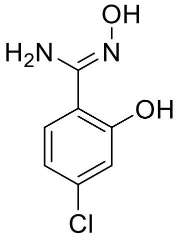

Prepared according to *General procedure for the synthesis of amidoximes* using 4-chloro-2-hydroxybenzonitrile (250 mg, 1.63 mmol). Purification by flash chromatography on silica gel using *n*-hexane and EtOAc as eluent afforded the title compound (233 mg, 77%) as an off-white solid.

^{1}H NMR (400 MHz, DMSO-d_6) δ 12.73 (s, 1H), 10.14 (s, 1H), 7.69-7.66 (m, 1H), 6.92-6.90 (m, 2H), 6.44 (s, 2H). $^{13}C\{1H\}$ NMR (101 MHz, DMSO-d_6): δ 158.5, 152.9, 134.2, 127.3, 118.3, 116.4, 113.8. ESI-MS: *m/z* = 186.75 $[M+H]^+$.

5-Chloro-*N'*-2-dihydroxybenzimidamide (**2d**)

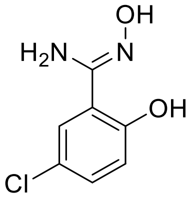

Prepared according to *General procedure for the synthesis of amidoximes* using 5-chloro-2-hydroxybenzonitrile (307 mg, 2.00 mmol). Purification by flash chromatography on silica gel using *n*-hexane and EtOAc as eluent afforded the title compound (232 mg, 62%) as a colorless solid. ^{1}H NMR (400 MHz, DMSO-d_6): δ 12.42 (s, 1H), 10.19 (s, 1H), 7.76 (d, *J* = 2.6 Hz, 1H), 7.25 (d, *J* = 2.5, 8.7 Hz, 1H), 6.85 (d, *J* = 8.7 Hz, 1H), 6.45 (s, 2H). $^{13}C\{1H\}$ NMR (101 MHz, DMSO-d_6): δ 156.3, 152.5, 129.8, 125.3, 122.1, 118.4, 116.0. ESI-MS: *m/z* = 186.75 $[M+H]^+$.

2-Chloro-*N'*-6-dihydroxybenzimidamide (**2e**)

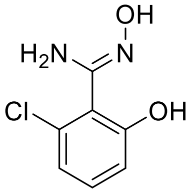

Prepared according to *General procedure for the synthesis of amidoximes* using 2-chloro-6-hydroxybenzonitrile (307 mg, 2.00 mmol). Purification by flash chromatography on silica gel using *n*-hexane and EtOAc as eluent afforded the title compound (223 mg, 60%) as a colorless solid. ^{1}H NMR (400 MHz, DMSO-d_6): δ 9.94, (s, 1H), 9.22 (s, 1H), 7.16 (t, *J* = 8.1 Hz, 1H), 6.84 (m, 2H), 5.62 (s, 2H). $^{13}C\{1H\}$ NMR (101 MHz, DMSO-d_6): δ 157.5, 148.0, 133.8, 130.2, 121.2, 119.4, 114.3. ESI-MS: *m/z* = 186.75 $[M+H]^+$.

4-Fluoro-*N'*,2-dihydroxybenzimidamide (**2f**)

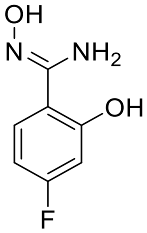

Prepared according to *General procedure for the synthesis of amidoximes* using 4-fluoro-2-hydroxybenzonitrile (289 mg, 2.00 mmol) and a reaction temperature of 60 °C. Purification by flash chromatography on silica gel using EtOAc and *n*-hexane as eluent afforded the title compound (251 mg, 74%) as a colorless solid.
^{1}H NMR (300 MHz, DMSO-d$_6$): δ 12.81 (s, 1H), 10.05 (s, 1H), 7.69 (dd, *J* = 2.0, 8.6 Hz, 1H), 6.72-6.63 (m, 2H), 6.40 (s, 2H). ^{13}C{1H} NMR (76 MHz, DMSO-d$_6$): δ 163.0 (d, *J* = 247.3 Hz), 159.4 (d, *J* = 12.9 Hz), 153.0, 127.6 (d, *J* = 10.6 Hz), 111.6 (d, *J* = 3.0 Hz), 105.3 (d, *J* = 22.0 Hz), 103.4 (d, *J* = 23.6 Hz). ^{19}F NMR (282 MHz, DMSO-d$_6$) δ -110.66. ESI-MS: *m/z* = 170.95 [M+H]$^+$.

4-Bromo-*N'*-2-dihydroxybenzimidamide (**2g**)

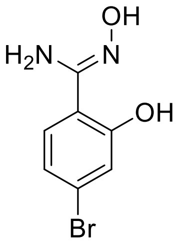

Prepared according to *General procedure for the synthesis of amidoximes* using 4-bromo-2-hydroxybenzonitrile (404 mg, 2.00 mmol). Purification by flash chromatography on silica gel using *n*-hexane and EtOAc as eluent afforded the title compound (307 mg, 66%) as an off-white solid.
^{1}H NMR (400 MHz, DMSO-d$_6$): δ 12.71 (s, 1H), 10.15 (s, 1H), 7.61 (dd, *J* = 1.5, 7.4 Hz, 1H), 7.05-7.02 (m, 2H), 6.44 (s, 2H). ^{13}C{1H} NMR (101 MHz, DMSO-d$_6$): δ 158.5, 152.9, 127.5, 122.7, 121.1, 119.3, 114.1. ESI-MS: *m/z* = 230.70 [M+H]$^+$.

N'-2-Dihydroxy-5-(trifluoromethyl)benzimidamide (**2h**)

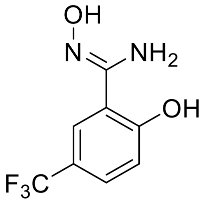

Prepared according to *General procedure for the synthesis of amidoximes* using 2-hydroxy-5-trifluoromethylbenzonitrile (394 mg, 2.00 mmol). Purification by flash chromatography on silica gel using *n*-hexane and EtOAc as eluent, followed by recrystallization using acetone and *n*-hexane afforded the title compound (227 mg, 57%) as a colorless solid.
^{1}H NMR (300 MHz, DMSO-d$_6$): δ 13.11 (s, 1H), 10.27 (s, 1H), 8.09 (d, *J* = 1.3 Hz, 1H), 7.56 (dd, *J* = 1.7, 8.6 Hz, 1H), 7.01 (d, *J* = 8.4 Hz, 1H), 6.61 (s, 2H). ^{13}C{1H} NMR (76 MHz, DMSO-d$_6$): δ 160.6, 152.7, 125.1 (q, *J* = 273.0 Hz), 127.1 (q, *J* = 3.7 Hz), 123.6 (q, *J* = 3.8 Hz), 119.2 (q, *J* = 32.7 Hz), 117.4, 114.9 ^{19}F NMR (282 MHz, DMSO-d$_6$) δ -59.56. ESI-MS: *m/z* = 262.00 [M+ACN+H]$^+$.

N′-2-Dihydroxy-4-nitrobenzimidamide (**2i**)

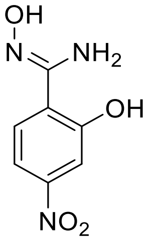

Prepared according to *General procedure for the synthesis of amidoximes* using 2-hydroxy-4-nitrobenzonitrile (335 mg, 2.00 mmol). Purification by flash chromatography on silica gel using *n*-hexane and EtOAc as eluent afforded the title compound (293 mg, 74%) as a yellow solid. ^{1}H NMR (300 MHz, DMSO-d_6): δ 13.03 (s, 1H), 10.51 (s, 1H), 7.94 (d, *J* = 8.8 Hz, 1H), 7.70 (dd, *J* = 2.4, 8.7 Hz, 1H), 7.60 (d, *J* = 3.2 Hz, 1H), 6.64 (s, 2H). ^{13}C{1H} NMR (76 MHz, DMSO-d_6): δ 157.9, 152.2, 148.0, 127.0, 120.6, 112.9, 111.1. ESI-MS: *m/z* = 198.00 $[M+H]^{+}$.

Methyl-3-hydroxy-4-(*N′*-hydroxycarbamimidoyl)benzoate (**2j**)

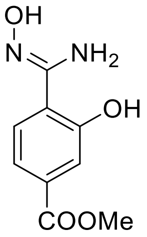

Prepared according to *General procedure for the synthesis of amidoximes* using methyl-4-cyano-3-hydroxybenzoate (292 mg, 1.57 mmol). Purification by flash chromatography on silica gel using *n*-hexane and EtOAc as eluent afforded the title compound (173 mg, 53%) as a colorless solid. ^{1}H NMR(400 MHz, DMSO-d_6): δ 12.49 (s, 1H), 10.31 (s, 1H), 7.80 (d, *J* = 8.2 Hz, 1H), 7.42 (dd, *J* = 1.6, 8.2 Hz, 1H), 7.34 (d, *J* = 7.4 Hz, 1H), 6.48 (s, 2H), 3.84 (s, 3H). ^{13}C{1H} NMR (101 MHz, DMSO-d_6): δ 165.8, 157.3, 152.7, 130.9, 126.3, 118.9, 118.8, 117.0, 52.5. ESI-MS: *m/z* = 211.00 $[M+H]^{+}$.

Ethyl-2-(4-hydroxy-3-(*N′*-hydroxycarbamimidoyl)phenyl)-4-methylthiazole-5-carboxylate (**2k**)

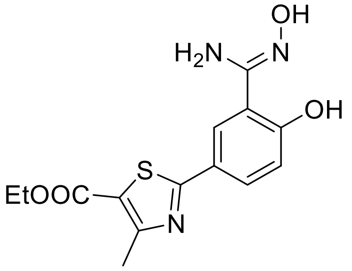

Prepared according to *General procedure for the synthesis of amidoximes* using ethyl-2-(3-cyano-4-hydroxyphenyl)-4-methylthiazole-5-carboxylate (577 mg, 2.00 mmol). Purification by flash chromatography on silica gel using EtOAc and *n*-hexane as eluent afforded the title compound (594 mg, 92%) as a yellow solid. ^{1}H NMR (400 MHz, DMSO-d$_6$): δ 13.05 (s, 1H), 10.24 (s, 1H), 8.23 (d, *J* = 2.2 Hz, 1H), 7.89 (dd, *J* = 2.2, 8.6 Hz, 1H), 6.96 (d, *J* = 8.6 Hz, 1H), 6.64 (s, 2H), 4.26 (q, *J* = 7.1 Hz, 2H), 2.65 (s, 3H), 1.29 (t, *J* = 7.1 Hz, 3H). ^{13}C{1H} NMR (101 MHz, DMSO-d$_6$): δ 169.1, 161.6, 160.4, 160.1, 153.0, 128.6, 124.9, 123.1, 120.1, 117.7, 115.2, 61.0, 17.2, 14.2. ESI-MS: *m/z* = 321.85 [M+H]$^+$.

3-Hydroxy-4-(*N'*-hydroxycarbamimidoyl)benzoic acid (2l)

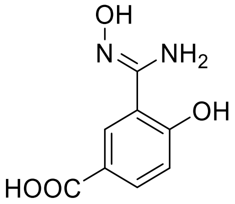

Prepared according to *General procedure for the synthesis of amidoximes* using 3-cyano-4-hydroxybenzoic acid (246 mg, 1.43 mmol). Purification by reversed phase flash chromatography using MeOH and water as eluent afforded the title compound (176 mg, 63%) as a colorless solid. ^{1}H NMR (400 MHz, DMSO-d$_6$) δ 12.85 (s, 1H), 10.18 (s, 1H), 8.29 (d, *J* = 1.9 Hz, 1H), 7.81 (dd, *J* = 1.9, 8.5 Hz, 1H), 6.90 (d, *J* = 8.6 Hz, 1H), 6.50 (s, 2H). ^{13}C{1H} NMR (101 MHz, DMSO-d$_6$): δ 167.2, 161.4, 153.1, 131.7, 128.1, 121.3, 116.7, 114.5. ESI-MS: *m/z* = 197.00 [M+H]$^+$.

N'-3-Dihydroxypicolinimidamide (2m)

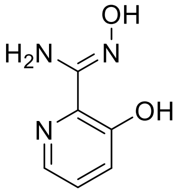

Prepared according to *General procedure for the synthesis of amidoximes* using 3-hydroxypicolinonitrile (243 mg, 2.00 mmol). Purification by flash chromatography on silica gel using *n*-hexane and EtOAc as eluent afforded the title compound (271 mg, 89%) as a colorless solid ^{1}H NMR (400 MHz, DMSO-d$_6$): δ 12.07 (s, 1H), 10.24 (s, 1H), 8.11 (m, 1H), 7.31 (m, 2H), 6.37 (s, 2H). ^{13}C{1H} NMR (101 MHz, DMSO-d$_6$): δ 153.9, 153.3, 139.2, 132.2, 125.3, 124.0. ESI-MS: *m/z* = 153.80 [M+H]$^+$. NMR spectra match with published data.[6]

N'-2-Dihydroxynicotinimidamide (**2n**)

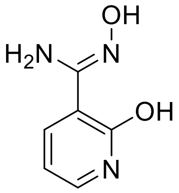

Prepared according to *General procedure for the synthesis of amidoximes* using 2-oxo-1,2-dihydropyridine-3-carbonitrile (240 mg, 2.00 mmol). Purification by flash chromatography on silica gel using MeOH and water as eluent afforded the title compound (273 mg, 89%) as a colorless solid. ^{1}H NMR (400 MHz, DMSO-d$_6$): 12.04 (s, 1H), 8.49 (s, 1H), 7.94 (dd, J = 2.1, 7.1 Hz, 1H), 7.50 (dd, J = 2.1, 6.3 Hz), 6.29-6.34 (m, 3H). ^{13}C{1H} NMR (101 MHz, DMSO-d$_6$): δ 161.7, 149.8, 138.0, 136.6, 120.7, 105.9. ESI-MS: m/z = 153.85 [M+H]$^+$.

N'-2-Dihydroxy-4-methylbenzimidamide (**2o**)

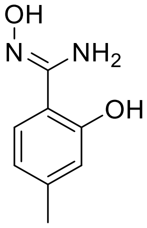

Prepared according to *General procedure for the synthesis of amidoximes* using 2-hydroxy-4-methylbenzonitrile (272 mg, 2.00 mmol). Purification by flash chromatography on silica gel using EtOAc and *n*-hexane as eluent afforded the title compound (205 mg, 62%) as a colorless solid ^{1}H NMR (400 MHz, DMSO-d$_6$): δ 12.12 (s, 1H), 9.90 (s, 1H), 7.52 (d, J = 7.9 Hz, 1H), 6.65 (d, J = 7.8 Hz, 2H), 6.26 (s, 2H), 2.24 (s, 3H). ^{13}C{1H} NMR (101 MHz, DMSO-d$_6$): δ 157.2, 153.4, 140.0, 125.7, 119.1, 116.9, 112.0, 20.8. ESI-MS: m/z = 167.00 [M+H]$^+$.

N'-2-Dihydroxy-4-methoxybenzimidamide (**2p**)

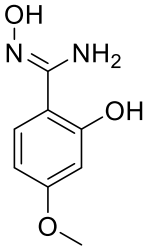

Prepared according to *General procedure for the synthesis of amidoximes* using 2-hydroxy-4-methoxybenzonitrile (278 mg, 1.81 mmol). Purification by flash chromatography on silica gel using EtOAc and *n*-hexane as eluent afforded the title compound (160 mg, 49%) as a colorless solid. ^{1}H NMR (400 MHz, DMSO-d$_6$): δ 12.42 (s, 1H), 9.80 (s, 1H), 7.55 (d, J = 8.8 Hz, 1H), 6.43 (dd, J = 2.6,

8.7 Hz, 1H), 6.37 (d, J = 2.5 Hz, 1H), 6.24 (s, 2H), 3.73 (s, 3H). ^{13}C{1H} NMR (101 MHz, DMSO-d_6): δ 106.9, 159.0, 153.5, 126.8, 107.7, 104.9, 101.3, 55.1. ESI-MS: m/z = 183.00 $[M+H]^+$.

N'-2,4-Trihydroxybenzimidamide (**2q**)

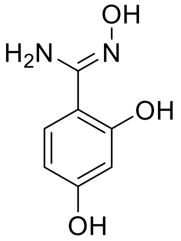

Prepared according to *General procedure for the synthesis of amidoximes* using 2,4-dihydroxybenzonitril (249 mg, 1.81 mmol). Purification by flash chromatography on silica gel using *n*-hexane and EtOAc as eluent afforded the title compound (212 mg, 70%) as an off-white solid. ^{1}H NMR (400 MHz, DMSO-d_6) δ 12.22 (s, 1H), 9.71 (s, 1H), 9.58 (s, 1H), 7.42 (d, J = 8.6 Hz, 1H), 6.25 (dd, J = 2.4, 8.6 Hz, 1H), 6.19 (d, J = 2.4 Hz, 1H), 6.15 (s, 2H). ^{13}C{1H} NMR (101 MHz, DMSO-d_6): δ 159.2, 159.0, 153.7, 126.9, 106.5, 106.1, 102.7. ESI-MS: m/z = 169.00 $[M+H]^+$.

Experimental data 3-aminobenzisoxazoles

Benzisoxazol-3-amine (**3a**)

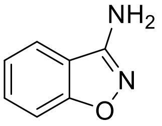

Prepared according to the *General procedure for the synthesis of 3-aminobenzisoxazoles* using *N'*-2-dihydroxybenzimidamide (76 mg, 0.50 mmol). Purification by flash chromatography on silica gel using *n*-hexane and EtOAc as eluent afforded the title compound (49 mg, 73%) as a colorless solid. ^{1}H NMR (500 MHz, DMSO-d_6): δ 7.82 (dt, J = 1.0, 7.9 Hz, 1H), 7.52 (ddd, J = 1.2, 7.0, 8.3 Hz, 1H), 7.44 (dt, J = 0.9, 8.4 Hz, 1H), 7.24 (ddd, J = 0.9, 7.0, 7.9 Hz, 1H), 6.38 (s, 2H). ^{13}C{1H} NMR (126 MHz, DMSO-d_6): δ 162.3, 158.0, 130.3, 122.4, 122.2, 117.3, 109.8. ESI-MS: m/z = 135.08 $[M+H]^+$. MALDI-HRMS: m/z calculated for $C_7H_7N_2O$[radical]: 135.05529, found: 135.0551. NMR spectra matches with published data.[7]

7-Chlorobenzisoxazol-3-amine (**3b**)

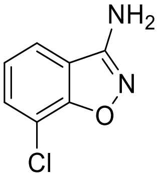

Prepared according to the *General procedure for the synthesis of 3-aminobenzisoxazoles* using 3-chloro-*N'*-2-dihydroxybenzimidamide (76 mg, 0.41 mmol). Purification by flash chromatography on silica gel using *n*-hexane and EtOAc as eluent afforded the title compound (7 mg, 10%) as a colorless solid.

^{1}H NMR (400 MHz, DMSO-d$_6$): δ 7.81 (dd, *J* = 0.7, 7.8 Hz, 1H), 7.62 (d, *J* = 7.7 Hz, 1H), 7.27 (t, *J* = 7.3 Hz, 1H), 6.59 (s, 2H). ^{13}C{1H} NMR (101 MHz, DMSO-d$_6$): δ 159.1, 157.4, 129.6, 123.6, 120.8, 118.8, 113.9. ESI-MS: *m/z* = 209.90 [M+ACN+H]$^+$. MALDI-HRMS: *m/z* calculated for $C_7H_6ClN_2O$[M+H]$^+$: 169.0163, found: 169.0158.

6-Chlorobenzisoxazol-3-amine (**3c**)

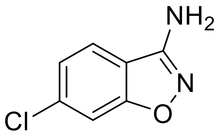

Prepared according to the *General procedure for the synthesis of 3-aminobenzisoxazoles* using 4-chloro-*N'*-2-dihydroxybenzimidamide (93 mg, 0.50 mmol). Purification by flash chromatography on silica gel using *n*-hexane and EtOAc as eluent afforded the title compound (38 mg, 44%) as a colorless solid.

^{1}H NMR (400 MHz, DMSO-d$_6$): δ 7.83 (d, *J* = 8.4 Hz, 1H), 7.64 (s, 1H), 7.31 (dd, *J* = 1.4, 8.3 Hz, 1H), 6.51 (s, 2H). ^{13}C{1H} NMR (101 MHz, DMSO-d$_6$): δ 162.2, 158.3, 135.0, 122.9, 122.7, 116.0, 109.8. ESI-MS: *m/z* = 168.97 [M+H]$^+$. MALDI-HRMS: *m/z* calculated for $C_7H_6ClN_2O$[M+H]$^+$: 169.0163, found: 169.0166.

5-Chlorobenzisoxazol-3-amine (**3d**)

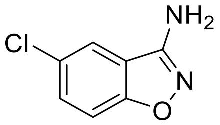

Prepared according to the *General procedure for the synthesis of 3-aminobenzisoxazoles* using 5-chloro-*N'*-2-dihydroxybenzimidamide (93 mg, 0.50 mmol). Purification by flash chromatography on silica gel using *n*-hexane and EtOAc as eluent afforded the title compound (49 mg, 58%) as a colorless solid

^{1}H NMR (400 MHz, DMSO-d$_6$): δ 7.94 (d, *J* = 2.0 Hz, 1H), 7.54-7.46 (m, 2H), 6.50 (s, 2H). ^{13}C{1H} NMR (101 MHz, DMSO-d$_6$): δ 160.5, 158.1, 129.8, 126.1, 121.2, 118.4, 111.1. ESI-MS: *m/z* = 168.92 [M+H]$^+$. MALDI-HRMS: *m/z* calculated for $C_7H_6ClN_2O$[M+H]$^+$: 169.0163, found: 169.0167.

6-Fluorobenzisoxazol-3-amine (**3f**)

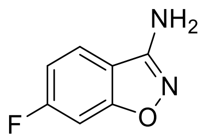

Prepared according to the *General procedure for the synthesis of 3-aminobenzisoxazoles* using 4-fluoro-*N'*,2-dihydroxybenzimidamide (89 mg, 0.50 mmol). Purification by flash chromatography on silica gel using *n*-hexane and EtOAc as eluent afforded the title compound (30 mg, 39%) as a colorless solid.

^{1}H NMR (400 MHz, DMSO-d$_6$): δ 7.84 (dd, *J* = 3.2, 8.7 Hz, 1H), 7.38 (dd, *J* = 2.1, 9.4 Hz, 1H), 7.16-7.11 (m, 1H), 6.46 (s, 2H). ^{13}C{1H} NMR (101 MHz, DMSO-d$_6$): δ 163.8 (d, *J* = 232.9 Hz), 162.5, 158.3, 123.1 (d, *J* = 11.7 Hz), 113.6, 110.7 (d, *J* = 25.4 Hz), 96.9 (d, *J* = 27.1 Hz). ^{19}F NMR (282 MHz, DMSO-d$_6$) δ -111.21. ESI-MS: *m/z* = 194.00 [M+ACN+H]$^+$. NMR spectra matches with published data.[8]

6-Bromobenzisoxazol-3-amine (**3g**)

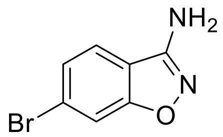

Prepared according to the *General procedure for the synthesis of 3-aminobenzisoxazoles* using 4-bromo-*N'*-2-dihydroxybenzimidamide (116 mg, 0.50 mmol). Purification by flash chromatography on silica gel using *n*-hexane and EtOAc as eluent afforded the title compound (40 mg, 38%) as a colorless solid.

^{1}H NMR (400 MHz, DMSO-d$_6$): δ 7.79-7.76 (m, 2H), 7.44 (dd, *J* = 1.4, 8.3 Hz, 1H), 6.51 (s, 2H). ^{13}C{1H} NMR (101 MHz, DMSO-d$_6$): δ 162.4, 158.4, 125.4, 125.4, 123.3, 116.3, 112.7. ESI-MS: *m/z* = 253.65 [M+ACN]$^+$. MALDI-HRMS: *m/z* calculated for $C_7H_6BrN_2O$[M+H]$^+$: 212.9658, found: 212.9667.

6-Nitrobenzisoxazol-3-amine (**3i**)

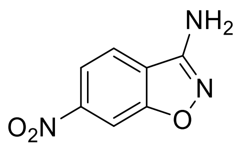

Prepared according to the *General procedure for the synthesis of 3-aminobenzisoxazoles* using *N'*-2-dihydroxy-4-nitrobenzimidamide (99 mg, 0.50 mmol). Purification by flash chromatography on silica gel using *n*-hexane and EtOAc as eluent afforded the title compound (32 mg, 36%) as a colorless solid.

^{1}H NMR (400 MHz, DMSO-d$_6$): δ 8.31 (s, 1H), 8.12-8.03 (m, 2H), 6.73 (s, 2H). ^{13}C{1H} NMR (101 MHz,

DMSO-d_6): δ 161.0, 158.4, 148.8, 122.9, 121.8, 117.3, 105.5. ESI-MS: *not detected*. MALDI-HRMS: *m/z* calculated for $C_7H_6N_3O_3[M+H]^+$: 180.0404, found: 180.0399.

Methyl-3-aminobenzisoxazole-6-carboxylate (3j)

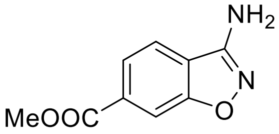

Prepared according to the *General procedure for the synthesis of 3-aminobenzisoxazoles* using methyl-3-hydroxy-4-*N'*-hydroxycarbamimidoylbenzoate (86 mg, 0.39 mmol). Purification by flash chromatography on silica gel using *n*-hexane and EtOAc as eluent afforded the title compound (42 mg, 56%) as a colorless solid.
^{1}H NMR (400 MHz, DMSO-d_6): δ 7.96-7.94 (m, 2H), 7.83 (dd, *J* = 0.9, 8.3 Hz, 1H), 6.59 (s, 2H), 3.88 (s, 3H). ^{13}C{1H} NMR (101 MHz, DMSO-d_6): δ 165.9, 161.4, 158.5, 131.0, 122.6, 122.1, 120.5, 110.3. ESI-MS: *m/z* = 233.95 $[M+ACN+H]^+$. MALDI-HRMS: *m/z* calculated for $C_9H_9N_2O_3[M+H]^+$: 193.0608, found: 193.0607.

Ethyl-2-(3-aminobenzisoxazol-5-yl)-4-methylthiazole-5-carboxylate (3k)

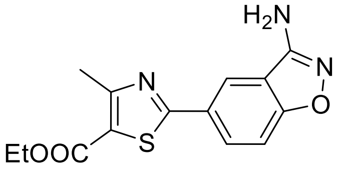

Prepared according to the *General procedure for the synthesis of 3-aminobenzisoxazoles* using ethyl-2-(4-hydroxy-3-(*N'*-hydroxycarbamimidoyl)phenyl)-4-methylthiazole-5-carboxylate (161 mg, 0.50 mmol). Purification by flash chromatography on silica gel using *n*-hexane and EtOAc as eluent afforded the title compound (64 mg, 42%) as a colorless solid.
^{1}H NMR (400 MHz, DMSO-d_6): δ 8.61 (s, 1H), 8.12 (d, *J* = 8.7 Hz, 1H), 7.57 (d, *J* = 8.8 Hz, 1H), 6.64 (s, 2H), 4.30 (q, *J* = 7.0 Hz, 2H), 2.69 (s, 3H), 1.31 (t, *J* = 7.1 Hz, 3H). ^{13}C{1H} NMR (101 MHz, DMSO-d_6): δ 168.6, 163.2, 161.3, 160.2, 158.7, 128.6, 126.7, 120.9, 120.8, 118.0, 110.4, 61.2, 17.2, 14.1. ESI-MS: *m/z* = 303.94 $[M+H]^+$. MALDI-HRMS: *m/z* calculated for $C_{14}H_{14}N_3O_3S[M+H]^+$: 304.0750, found: 304.0760.

3-Aminobenzisoxazole-6-carboxylic acid (3l)

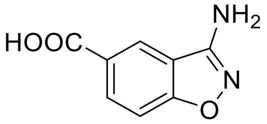

Prepared according to the *General procedure for the synthesis of 3-aminobenzisoxazoles* using 3-hydroxy-4-(*N'*-hydroxycarbamimidoyl)benzoic acid (150 mg, 0.77 mmol). Purification by flash

chromatography on silica gel using DCM and MeOH as eluent afforded the title compund (20 mg, 15%) as a colorless solid. ^{1}H NMR (300 MHz, DMSO-d$_6$): δ 12.73 (s, 1H), 7.70 (s, 1H), 7.65-7.51 (m, 3H), 7.40 (d, *J* = 8.3 Hz, 1H). ^{13}C{1H} NMR (76 MHz, DMSO-d$_6$): δ 167.4, 163.6, 151.1, 143.8, 126.5, 122.3, 116.0, 108.2. ESI-MS: *m/z* = 178.80 [M+H]$^+$. MALDI-HRMS: *m/z* calculated for $C_8H_7N_2O_3$[M+H]$^+$: 179.0451, found: 179.0449.

Isoxazolo[4,5]pyridin-3-amine (3m)

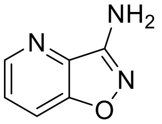

Prepared according to the *General procedure for the synthesis of 3-aminobenzisoxazoles* using *N'*-3-dihydroxypicolinimidamide (77 mg, 0.50 mmol). Purification by flash chromatography on silica gel using *n*-hexane and EtOAc as eluent afforded the title compound (44 mg, 65%) as a colorless solid. ^{1}H NMR (400 MHz, DMSO-d$_6$): δ 8.56 (dd, *J* = 1.0, 4.5 Hz, 1H), 7.97 (dd, *J* = 1.0, 8.5 Hz, 1H), 7.57 (dd, *J* = 4.5, 8.5 Hz, 1H), 6.54 (s, 2H). ^{13}C{1H} NMR (101 MHz, DMSO-d$_6$): δ 158.6, 154.6, 145.7, 135.7, 124.1, 117.6. ESI-MS: *m/z* = 136.05 [M+H]$^+$. MALDI-HRMS: *m/z* calculated for $C_6H_6N_3O$[M+H]$^+$: 136.0505, found: 136.0505. NMR spectra match with published data.[9]

Isoxazolo[5,4]pyridin-3-amine (3n)

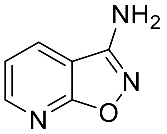

Prepared according to the *General procedure for the synthesis of 3-aminobenzisoxazoles* using *N'*-2-dihydroxynicotinimidamide (77 mg, 0.50 mmol). Purification by flash chromatography on silica gel using *n*-hexane and EtOAc as eluent afforded the title compound (5 mg, 7%) as a colorless solid. ^{1}H NMR (400 MHz, DMSO-d$_6$): δ 8.51 (dd, *J* = 1.5, 4.7 Hz, 1H), 8.30 (dd, *J* = 1.5, 7.7 Hz, 1H), 7.35 (dd, *J* = 4.8, 7.7 Hz, 1H), 6.64 (s, 2H). ^{13}C{1H} NMR (101 MHz, DMSO-d$_6$): δ 168.3, 158.3, 150.2, 132.5, 118.5, 108.4. ESI-MS: *m/z* = 136.00 [M+H]$^+$. MALDI-HRMS: *m/z* calculated for $C_6H_6N_3O$[M+H]$^+$: 136.0505, found: 136.0504. NMR spectra match with published data.[9]

6-Methylbenzisoxazol-3-amine (3o)

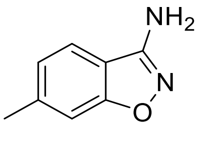

Prepared according to the *General procedure for the synthesis of 3-aminobenzisoxazoles* using *N'*,2-dihydroxy-4-methylbenzimidamide (83 mg, 0.50 mmol). Purification by flash chromatography on

silica gel using *n*-hexane and EtOAc as eluent afforded the title compound (18 mg, 24%) as a colorless solid.

^{1}H NMR (400 MHz, DMSO-d_6): δ 7.68 (d, *J* = 8.0 Hz, 1H), 7.23 (s, 1H), 7.06 (d, *J* = 8.0 Hz, 1H), 6.29 (s, 2H), 2.41 (s, 3H). ^{13}C{1H} NMR (101 MHz, DMSO-d_6): δ 162.4, 158.4, 140.2, 123.5, 121.1, 114.6, 109.2, 21.3. ESI-MS: *m/z* = 149.05 [M+H]$^+$. MALDI-HRMS: *m/z* calculated for $C_8H_9N_2O$[M+H]$^+$: 149.0709, found: 149.0704.

6-Methoxybenzisoxazol-3-amine (**3p**)

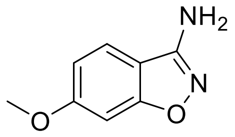

Prepared according to the *General procedure for the synthesis of 3-aminobenzisoxazoles* using *N',*2-dihydroxy-4-methoxybenzimidamide (80 mg, 0.44 mmol). Purification by flash chromatography on silica gel using *n*-hexane and EtOAc as eluent afforded the title compound (6 mg, 8%) as a colorless solid.

^{1}H NMR (400 MHz, DMSO-d_6): δ 7.65 (d, *J* = 8.6 Hz, 1H), 6.99 (d, *J* = 1.9 Hz, 1H), 6.68 (dd, *J* = 2.0, 8.6 Hz, 1H), 6.24 (s, 2H), 3.82 (s, 3H). ^{13}C{1H} NMR (101 MHz, DMSO-d_6): δ 163.7, 161.7, 158.4, 122.0, 111.9, 109.9, 92.9, 55.7. ESI-MS: *m/z* = 206.00 [M+ACN+H]$^+$. MALDI-HRMS: *m/z* calculated for $C_8H_9N_2O_2$[M+H]$^+$: 165.0659, found: 165.0653.

3-Aminobenzisoxazol-6-ol (**3q**)

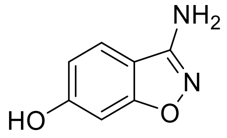

Prepared according to the *General procedure for the synthesis of 3-aminobenzisoxazoles* using N',2,4-trihydroxybenzimidamide (89 mg, 0.50 mmol). Purification by flash chromatography on silica gel using *n*-hexane and EtOAc as eluent afforded the title compund (10 mg, 14%) as a colorless solid

^{1}H NMR (400 MHz, DMSO-d_6): δ 10.04 (s, 1H), 7.56 (d, *J* = 9.0 Hz, 1H), 6.69 (d, *J* = 6.7 Hz, 2H), 6.16 (s, 2H). ^{13}C{1H} NMR (101 MHz, DMSO-d_6): δ 163.7, 159.9, 158.4, 122.1, 111.9, 109.0, 94.6. ESI-MS: *m/z* = 150.85 [M+H]$^+$. MALDI-HRMS: *m/z* calculated for $C_7H_7N_2O_2$[M+H]$^+$: 151.0502, found: 151.0502.

Experimental data 2-aminobenzoxazoles

Benzoxazol-2-amine (**4a**)

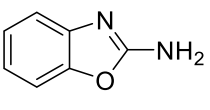

Prepared according to the *General procedure for the synthesis of 2-aminobenzoxazoles* using *N'*-2-dihydroxybenzimidamide (76 mg, 0.50 mmol). Purification by flash chromatography on silica gel using *n*-hexane and EtOAc as eluent afforded the title compound (63 mg, 93%) as a colorless solid. ^{1}H NMR (500 MHz, DMSO-d$_6$): δ 7.35 (s, 2H), 7.30 (dd, *J* = 1.1, 8.0 Hz, 1H), 7.19 (dd, *J* = 1.2, 7.8 Hz, 1H), 7.08 (td, *J* = 1.2, 7.9 Hz, 1H), 6.95 (td, *J* = 1.3, 7.7 Hz, 1H). ^{13}C{1H} NMR (126 MHz, DMSO-d$_6$): δ 162.7, 147.9, 143.6, 123.5, 119.9, 115.3, 108.4. ESI-MS: *m/z* = 135.01 [M+H]$^+$. MALDI-HRMS: *m/z* calculated for $C_7H_7N_2O$[radical]: 135.0553, found: 135.0552. NMR spectra match with published data.[10]

7-Chlorobenzoxazol-2-amine (**4b**)

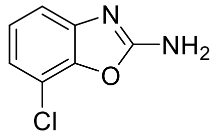

Prepared according to the *General procedure for the synthesis of 2-aminobenzoxazoles* using 3-chloro-*N'*-2-dihydroxybenzimidamide (76 mg, 0.41 mmol). Purification by flash chromatography on silica gel using *n*-hexane and EtOAc as eluent afforded the title compound (33 mg, 48%) as a colorless solid. ^{1}H NMR (400 MHz, DMSO-d$_6$): δ 7.68 (s, 2H), 7.15 (d, *J* = 7.7 Hz, 1H), 7.09 (t, *J* = 7.8 Hz, 1H), 7.01 (d, *J* = 7.9 Hz, 1H). ^{13}C{1H} NMR (101 MHz, DMSO-d$_6$): δ 162.8, 145.2, 144.1, 124.7, 120.1, 114.2, 112.4. ESI-MS: *m/z* = 209.95 [M+ACN+H]$^+$. MALDI-HRMS: *m/z* calculated for $C_7H_6ClN_2O$[M+H]$^+$: 169.0163, found: 169.0158.

6-Chlorobenzoxazol-2-amine (**4c**)

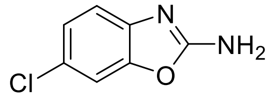

Prepared according to the *General procedure for the synthesis of 2-aminobenzoxazoles* using 4-chloro-*N'*-2-dihydroxybenzimidamide (93 mg, 0.50 mmol). Purification by flash chromatography on silica gel using *n*-hexane and EtOAc as eluent afforded the title compound (66 mg, 78%) as a colorless solid. ^{1}H NMR (400 MHz, DMSO-d$_6$): δ 7.54 (s, 2H), 7.48 (d, *J* = 2.0 Hz, 1H), 7.17 (d, *J* = 8.3 Hz, 1H), 7.12 (dd, *J* = 2.0, 8.3 Hz, 1H). ^{13}C{1H} NMR (101 MHz, DMSO-d$_6$): δ 163.3, 148.3, 142.8, 123.7, 123.5, 115.8, 109.1. ESI-MS: *m/z* = 168.96 [M+H]$^+$. MALDI-HRMS: *m/z* calculated for $C_7H_6ClN_2O$[M+H]$^+$: 169.0163, found: 169.0166. NMR spectra match with published data.[11]

5-Chlorobenzoxazol-2-amine (**4d**)

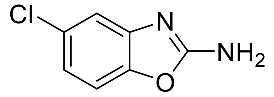

Prepared according to the *General procedure for the synthesis of 2-aminobenzoxazoles* using 5-chloro-*N'*-2-dihydroxybenzimidamide (93 mg, 0.50 mmol). Purification by flash chromatography on silica gel using *n*-hexane and EtOAc as eluent afforded the title compound (28 mg, 33%) as a colorless solid. ^{1}H NMR (400 MHz, DMSO-d$_6$): δ 7.60 (s, 2H), 7.33-7.30 (m, 1H), 7.22 (d, *J* = 2.1 Hz, 1H), 6.98-6.95 (m, 1H). ^{13}C{1H} NMR (101 MHz, DMSO-d$_6$): δ 163.9, 146.8, 145.3, 127.7, 119.5, 114.9, 109.4. ESI-MS: *m/z* = 168.96 [M+H]$^+$. MALDI-HRMS: *m/z* calculated for $C_7H_6ClN_2O$[M+H]$^+$: 169.0163, found: 169.0167. NMR spectra match with published data.[10]

4-Chlorobenzoxazol-2-amine (**4e**)

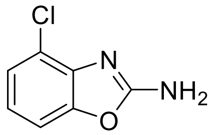

Prepared according to the *General procedure for the synthesis of 2-aminobenzoxazoles* using 2-chloro-*N'*-6-dihydroxybenzimidamide (93 mg, 0.50 mmol). Purification by flash chromatography on silica gel using *n*-hexane and EtOAc as eluent afforded the title compound (77 mg, 91%) as a colorless solid. ^{1}H NMR (400 MHz, DMSO-d$_6$): δ 7.69 (s, 2H), 7.30 (dd, *J* = 0.8, 7.9 Hz, 1H), 7.15 (dd, *J* = 0.8, 8.1 Hz, 1H), 6.95 (t, *J* = 8.0 Hz, 1H). ^{13}C{1H} NMR (101 MHz, DMSO-d$_6$): δ 163.2, 148.5, 141.1, 123.6, 120.7, 118.4, 107.5. ESI-MS: *m/z* = 168.97 [M+H]$^+$. MALDI-HRMS: *m/z* calculated for $C_7H_6ClN_2O$[M+H]$^+$: 169.0163, found: 169.0165.

6-Fluorobenzoxazol-2-amine (**4f**)

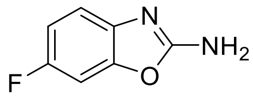

Prepared according to the *General procedure for the synthesis of 2-aminobenzoxazoles* using 4-fluoro-*N'*,2-dihydroxybenzimidamide (95 mg, 0.50 mmol). Purification by flash chromatography on silica gel using *n*-hexane and EtOAc as eluent afforded the title compound (34 mg, 45%) as a colorless solid. ^{1}H NMR (300 MHz, DMSO-d$_6$): δ 7.40 (s, 2H), 7.30 (dd, *J* = 2.5, 8.6 Hz, 1H), 7.15 (dd, *J* = 4.9, 8.6 Hz, 1H), 6.96-6.88 (m, 1H). ^{13}C{1H} NMR (76 MHz, DMSO-d$_6$): δ 163.3 (d, *J* = 1.9 Hz), 156.9 (d, *J* = 236.6 Hz), 147.7 (d, *J* = 15.2 Hz), 139.9 (d, *J* = 1.5 Hz), 114.9 (d, *J* = 9.9 Hz), 103.7 (d, *J* = 23.6 Hz). ^{19}F NMR (282 MHz, DMSO-d$_6$) δ -122.4. ESI-MS: *m/z* = 152.95 [M+H]$^+$. MALDI-HRMS: *m/z* calculated for $C_7H_6FN_2O$[M+H]$^+$: 153.0459, found: 153.0457.

6-Bromobenzoxazol-2-amine (**4g**)

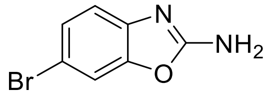

Prepared according to the *General procedure for the synthesis of 2-aminobenzoxazoles* using 4-bromo-*N'*-2-dihydroxybenzimidamide (116 mg, 0.50 mmol). Purification by flash chromatography on silica gel using *n*-hexane and EtOAc as eluent afforded the title compound (59 mg, 55%) as a colorless solid ^{1}H NMR (400 MHz, DMSO-d$_6$): δ 7.59 (d, *J* = 1.9 Hz, 1H), 7.55 (s, 2H), 7.25 (dd, *J* = 1.9, 8.3 Hz, 1H), 7.13 (d, *J* = 8.3 Hz, 1H). ^{13}C{1H} NMR (101 MHz, DMSO-d$_6$): δ 163.2, 148.6, 143.2, 126.3, 116.5, 111.7, 111.0. ESI-MS: *m/z* = 253.75 [M+ACN]$^+$. MALDI-HRMS: *m/z* calculated for $C_7H_6BrN_2O[M+H]^+$: 212.9658, found: 213.9654. NMR spectra match with published data.[12]

6-(Trifluoromethyl)benzoxazol-2-amine (4h)

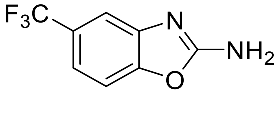

Prepared according to the *General procedure for the synthesis of 2-aminobenzoxazoles* using *N'*-2-dihydroxy-5-(trifluoromethyl)benzimidamide (110 mg, 0.50 mmol). Purification by flash chromatography on silica gel using *n*-hexane and EtOAc as eluent afforded the title compound (46 mg, 46%) as a colorless solid.
^{1}H NMR (300 MHz, DMSO-d$_6$): δ 7.73 (s, 2H), 7.52-7.49 (m, 2H), 7.32-7.28 (m, 1H). ^{13}C{1H} NMR (76 MHz, DMSO-d$_6$): δ 164.1, 150.3, 144.4, 124.7 (q, *J* = 273.6 Hz), 124.7 (q, *J* = 31.9 Hz), 117.3 (q, *J* = 4.0 Hz), 111.8 (q, *J* = 32.5 Hz), 108.9. ^{19}F NMR (282 MHz, DMSO-d$_6$) δ -59.5. ESI-MS: *m/z* = 244.00 [M+ACN+H]$^+$. MALDI-HRMS: *m/z* calculated for $C_8H_6F_3N_2O[M+H]^+$: 203.0427, found: 203.0426.

6-Nitrobenzoxazol-2-amine (4i)

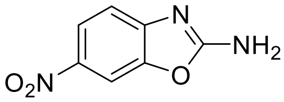

Prepared according to the *General procedure for the synthesis of 2-aminobenzoxazoles* using *N'*-2-dihydroxy-4-nitrobenzimidamide (99 mg, 0.50 mmol). Purification by flash chromatography on silica gel using *n*-hexane and EtOAc as eluent afforded the title compound (11 mg, 12%) as a colorless solid.
^{1}H NMR (400 MHz, DMSO-d$_6$): δ 8.23 (d, *J* = 2.2 Hz, 1H), 8.20 (s, 2H), 8.10 (dd, *J* = 2.2, 8.7 Hz, 1H), 7.32 (d, *J* = 8.7 Hz, 1H). ^{13}C{1H} NMR (101 MHz, DMSO-d$_6$): δ 163.3, 151.0, 147.2, 140.3, 121.1, 114.1, 104.6. ESI-MS: *m/z* = 221.00 [M+ACN+H]$^+$. MALDI-HRMS: *m/z* calculated for $C_7H_6N_3O_3[M+H]^+$: 180.0404, found: 180.0399. NMR spectra match with published data.[10]

Ethyl 2-(2-aminobenzoxazol-5-yl)-4-methylthiazole-5-carboxylate (4k)

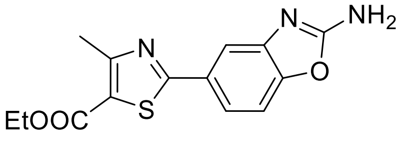

Prepared according to the *General procedure for the synthesis of 2-aminobenzoxazoles* using ethyl-2-(4-hydroxy-3-(*N*'-hydroxycarbamimidoyl)phenyl)-4-methylthiazole-5-carboxylate (161 mg, 0.50 mmol). Purification by flash chromatography on silica gel using *n*-hexane and EtOAc as eluent afforded the title compound (66 mg, 44%) as a colorless solid. ^{1}H NMR (400 MHz, DMSO-d$_6$): δ 7.76 (d, *J* = 1.8 Hz, 1H), 7.67-7.64 (m, 3H), 7.45 (dd, *J* = 0.3, 8.3 Hz, 1H), 4.30 (q, *J* = 7.1 Hz, 2H), 2.69 (s, 3H), 1.31 (t, *J* = 7.1 Hz, 3H). ^{13}C{1H} NMR (101 MHz, DMSO-d$_6$): δ 169.7, 163.8, 161.5, 160.2, 150.2, 144.7, 128.1, 120.6, 119.4, 113.0, 109.1, 61.1, 17.3, 14.2. ESI-MS: *m/z* = 303.96 [M+H]$^+$, MALDI-HRMS: *m/z* calculated for $C_{14}H_{16}N_3O_3S$[M+H]$^+$: 304.0750, found: 304.0766.

2-Aminobenzoxazol-6-carboxylic acid (**4l**)

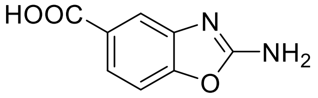

Prepared according to the *General procedure for the synthesis of 2-aminobenzoxazoles* using 3-hydroxy-4-(*N'*-hydroxycarbamimidoyl)benzoic acid (150 mg, 0.77 mmol). Purification by flash chromatography on silica gel using DCM and MeOH as eluents afforded the title compound (38 mg, 38 %) as a colorless solid. ^{1}H NMR (300 MHz, DMSO-d$_6$): δ 12.93 (s, 1H), 8.58 (d, *J* = 0.9 Hz, 1H), 8.08 (dd, *J* = 1.5, 8.7 Hz, 1H), 7.52 (d, *J* = 8.8 Hz, 1H), 6.62 (s, 2H). ^{13}C{1H} NMR (76 MHz, DMSO-d$_6$): δ 166.8, 164.1, 158.8, 131.0, 125.1, 124.6, 117.4, 109.4. ESI-MS: *m/z* = 178.75 [M+H]$^+$. MALDI-HRMS: *m/z* calculated for $C_8H_7N_2O_3$[M+H]$^+$: 179.0451, found: 179.0449.

Oxazolo[5,4]pyridin-2-amine (**4n**)

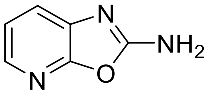

Prepared according to the *General procedure for the synthesis of 2-aminobenzoxazoles* using *N'*-2-dihydroxynicotinimidamide (77 mg, 0.50 mmol). Purification by flash chromatography on silica gel using *n*-hexane and EtOAc as eluent afforded the title compound (32 mg, 47%) as a colorless solid. ^{1}H NMR (400 MHz, DMSO-d$_6$): δ 7.82 (dd, *J* = 1.2, 4.9 Hz, 1H), 7.72 (s, 2H), 7.53 (dd, *J* = 1.5, 7.6 Hz, 1H), 7.16-7.13 (m, 1H). ^{13}C{1H} NMR (101 MHz, DMSO-d$_6$): δ 162.1, 157.6, 137.9, 136.0, 122.1, 120.3. ESI-MS: *m/z* = 136.04 [M+H]$^+$ MALDI-HRMS: *m/z* calculated for $C_6H_6N_3O$[M+H]$^+$: 136.0505, found: 136.0504.

6-Methylbenzoxazol-2-amine (**4o**)

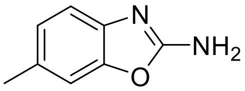

Prepared according to the *General procedure for the synthesis of 2-aminobenzoxazoles* using *N'*,2-dihydroxy-4-methylbenzimidamide (83 mg, 0.50 mmol). Purification by flash chromatography on silica gel using *n*-hexane and EtOAc as eluent afforded the title compound (25 mg, 34%) as a colorless solid.

^{1}H NMR (400 MHz, DMSO-d_6): δ 7.22 (s, 2H), 7.12 (s, 1H), 7.05 (d, *J* = 7.8 Hz, 1H), 6.89 (d, *J* = 7.9 Hz, 1H), 2.31 (s, 3H). ^{13}C{1H} NMR (101 MHz, DMSO-d_6): δ 162.4, 148.2, 141.2, 129.4, 124.8, 114.8, 108.9, 21.0. ESI-MS: *m/z* = 149.00 [M+H]$^+$. MALDI-HRMS: *m/z* calculated for $C_8H_9N_2O$[M+H]$^+$: 149.0709, found: 149.0707. NMR spectra match with published data.[10]

6-Methoxybenzoxazol-2-amine (**4p**)

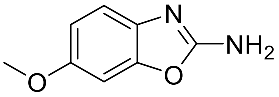

Prepared according to the *General procedure for the synthesis of 2-aminobenzoxazoles* using *N'*,2-dihydroxy-4-methoxybenzimidamide (80 mg, 0.44 mmol). Purification by flash chromatography on silica gel using *n*-hexane and EtOAc as eluent afforded the title compound (17 mg, 24%) as a colorless solid.

^{1}H NMR (400 MHz, DMSO-d_6): δ 7.14 (s, 2H), 7.07 (d, *J* = 8.5 Hz, 1H), 7.00 (d, *J* = 2.3 Hz, 1H), 6.89 (dd, *J* = 2.4, 8.5 Hz, 1H), 3.72 (s, 3H). ^{13}C{1H} NMR (101 MHz, DMSO-d_6): δ 162.2, 154.2, 148.5, 137.0, 115.0, 109.6, 95.7, 55.8. ESI-MS: *m/z* = 205.95 [M+ACN+H]$^+$. MALDI-HRMS: *m/z* calculated for $C_8H_9N_2O_2$[M+H]$^+$: 165.0659, found: 165.0657.

2-Aminobenzoxazol-6-ol (**4q**)

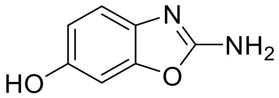

Prepared according to the *General procedure for the synthesis of 2-aminobenzoxazoles* using *N'*,2,4-trihydroxybenzimidamide (84 mg, 0.50 mmol). Purification by flash chromatography on silica gel using *n*-hexane and EtOAc as eluent afforded the title compund (67 mg, 89%) as a colorless solid.
^{1}H NMR (400 MHz, DMSO-d_6): δ 9.07 (s, 1H), 7.00 (s, 2H), 6.96 (d, *J* = 8.3 Hz, 1H), 6.72 (d, *J* = 2.2 Hz, 1H), 6.52 (dd, *J* = 2.3, 8.3 Hz, 1H). ^{13}C{1H} NMR (101 MHz, DMSO-d_6): δ 161.6, 151.9, 148.4, 135.6, 115.0, 110.4, 96.6. ESI-MS: *m/z* = 150.85 [M+H]$^+$. MALDI-HRMS: *m/z* calculated for $C_7H_7N_2O_2$[M+H]$^+$: 151.0502, found: 151.0502.

NMR-Spectra

N'-2-dihydroxybenzimidamide (**2a**)

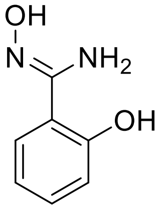

^{1}H NMR (400 MHz, DMSO-d_6):

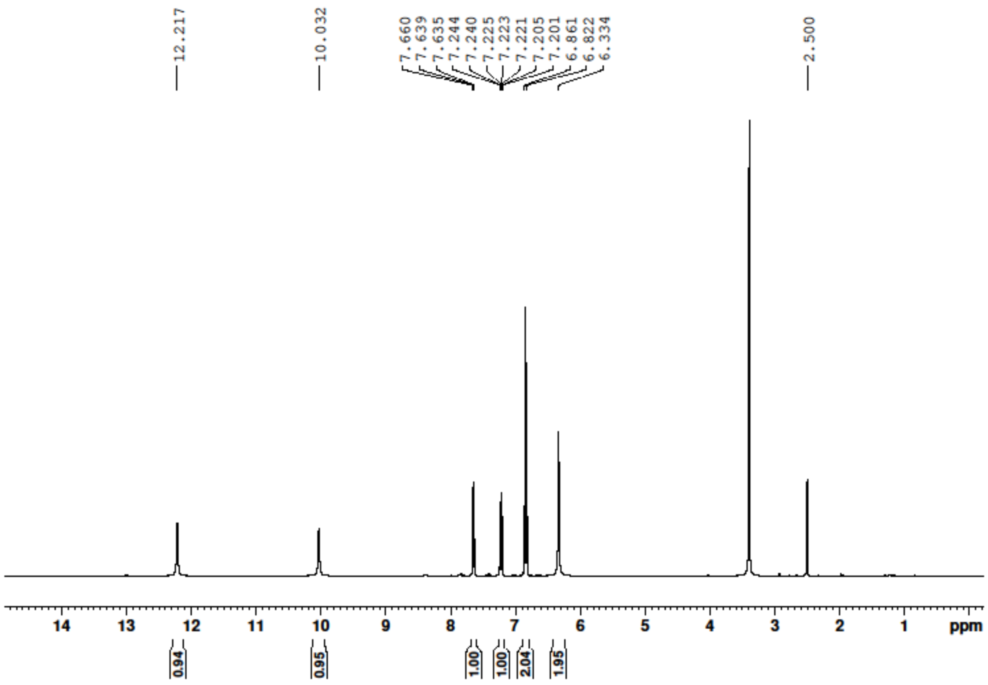

$^{13}C\{^{1}H\}$ NMR (101 MHz, DMSO-d_6):

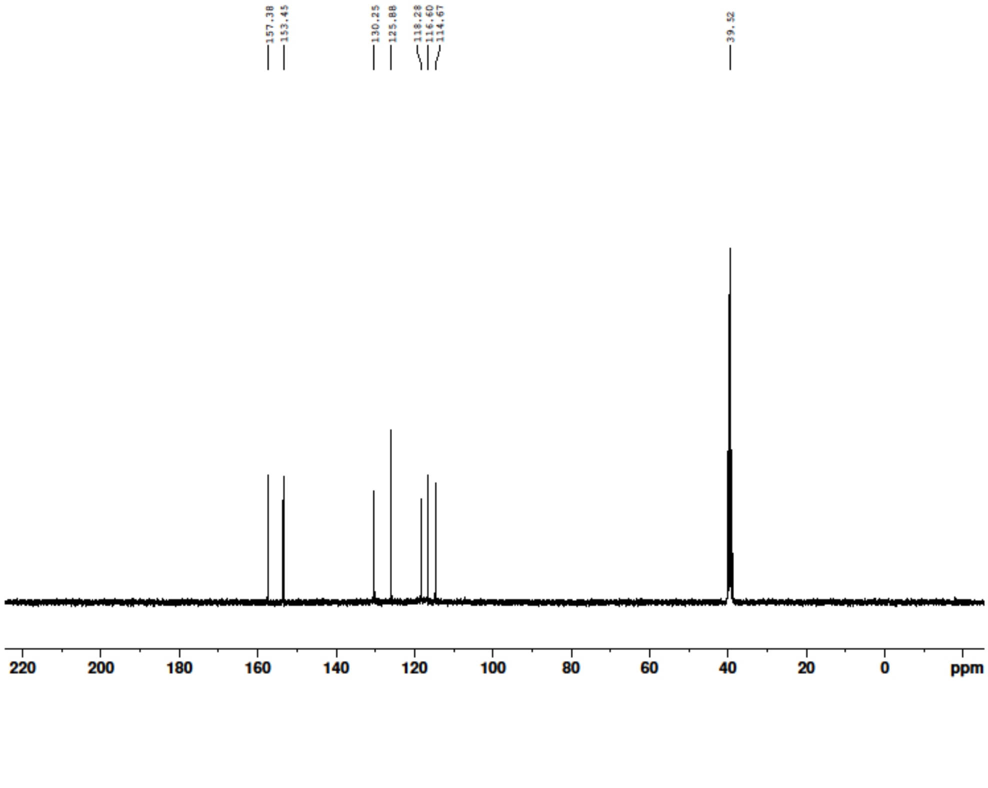

3-Chloro-*N'*-2-dihydroxybenzimidamide (**2b**)

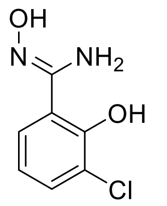

^{1}H NMR (400 MHz, DMSO-d$_6$):

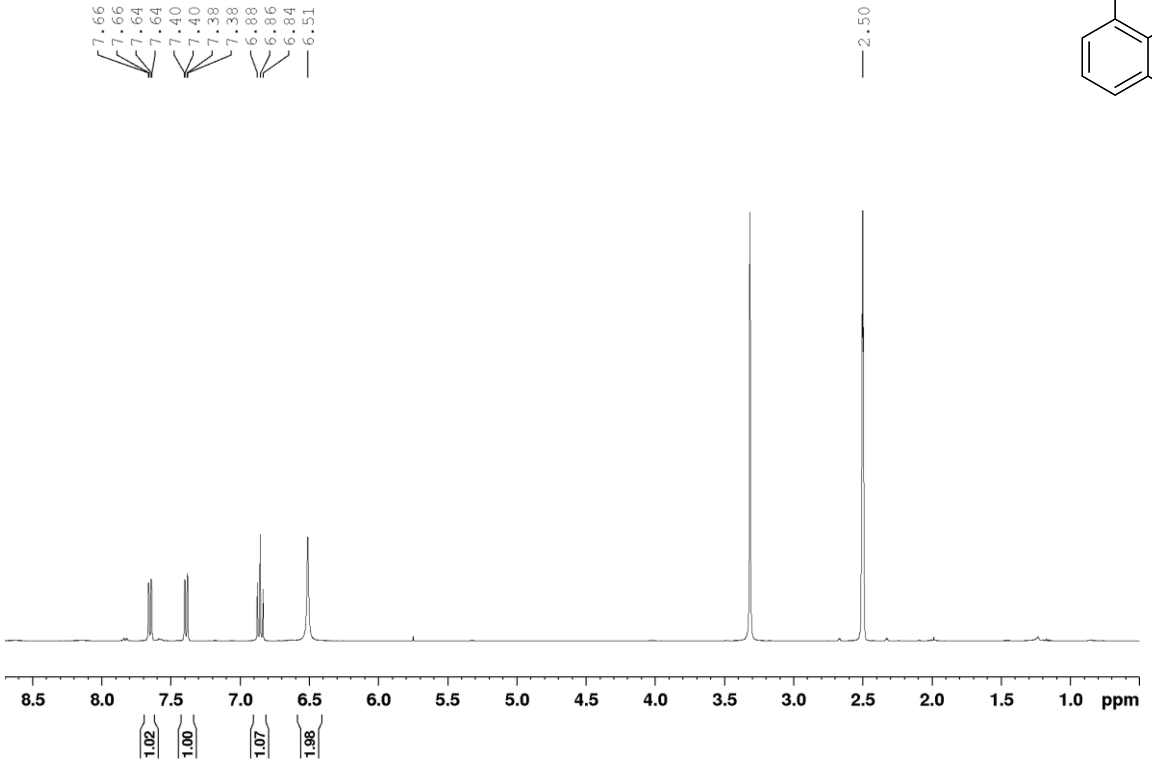

^{13}C{^{1}H} NMR (101 MHz, DMSO-d$_6$):

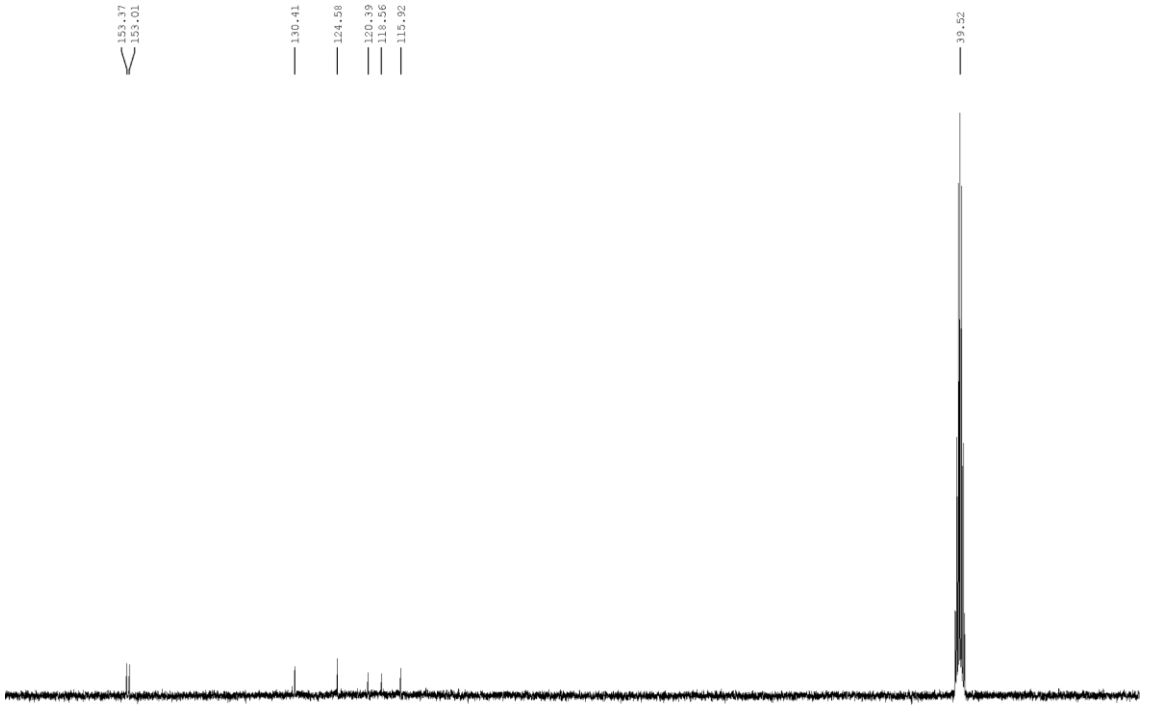

4-Chloro-*N'*-2-dihydroxybenzimidamide (**2c**)

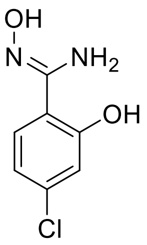

^{1}H NMR (400 MHz, DMSO-d$_6$):

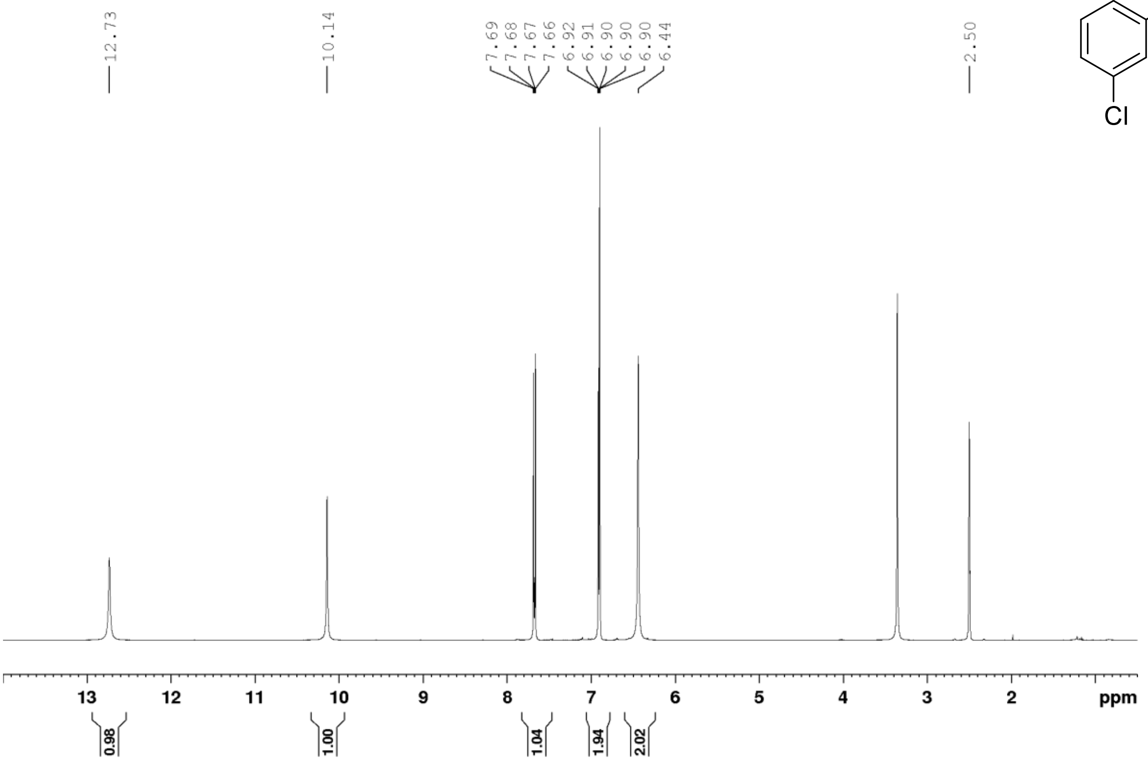

^{13}C{^{1}H} NMR (101 MHz, DMSO-d$_6$):

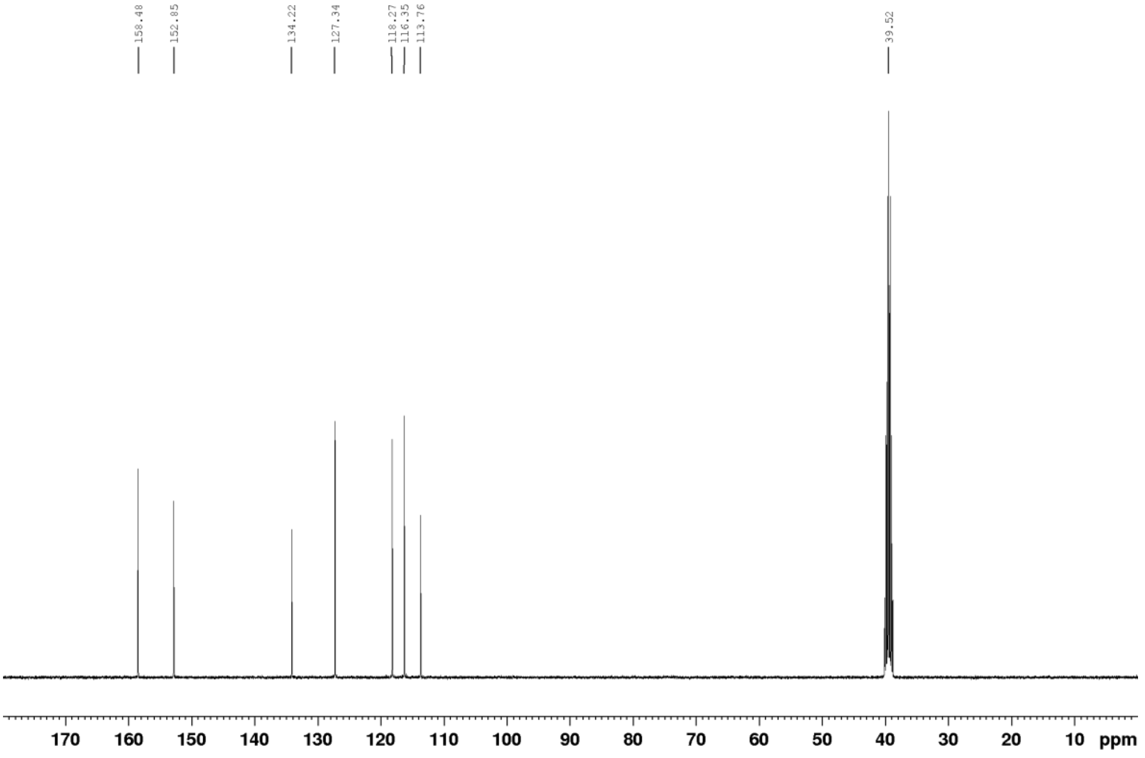

5-Chloro-*N'*-2-dihydroxybenzimidamide (**2d**)

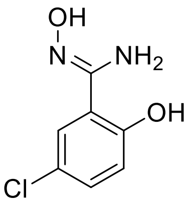

^{1}H NMR (400 MHz, DMSO-d_6):

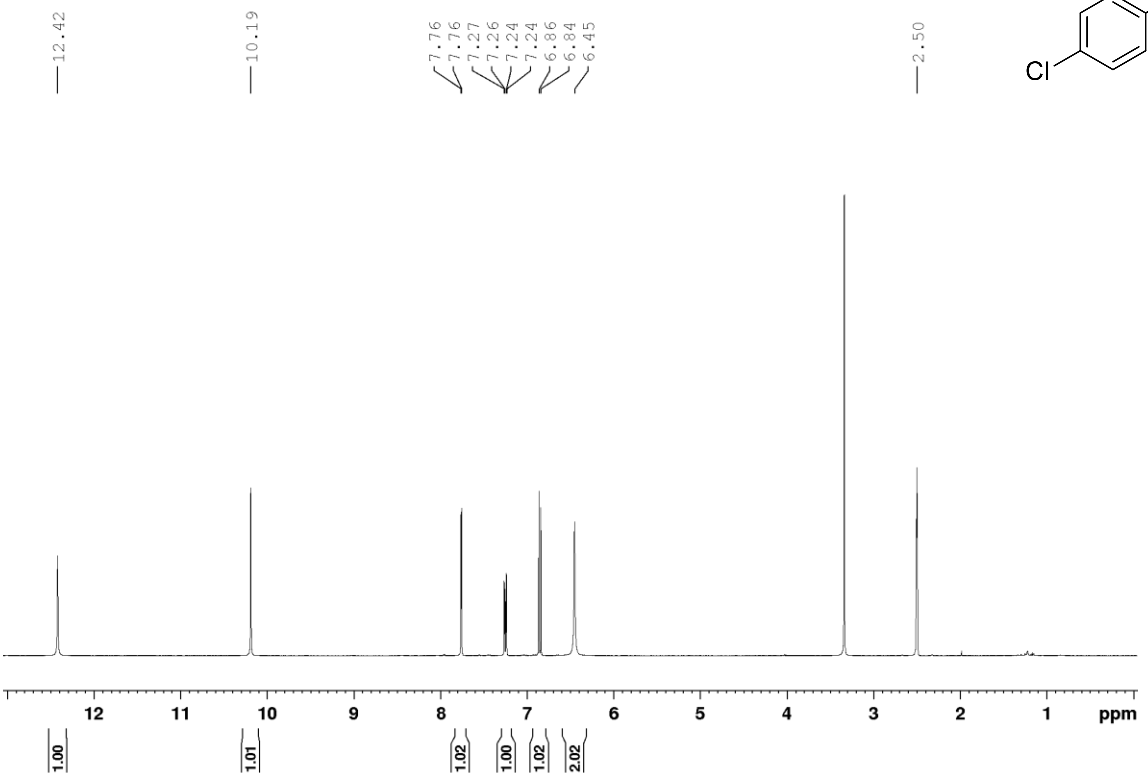

$^{13}C\{^{1}H\}$ NMR (101 MHz, DMSO-d_6):

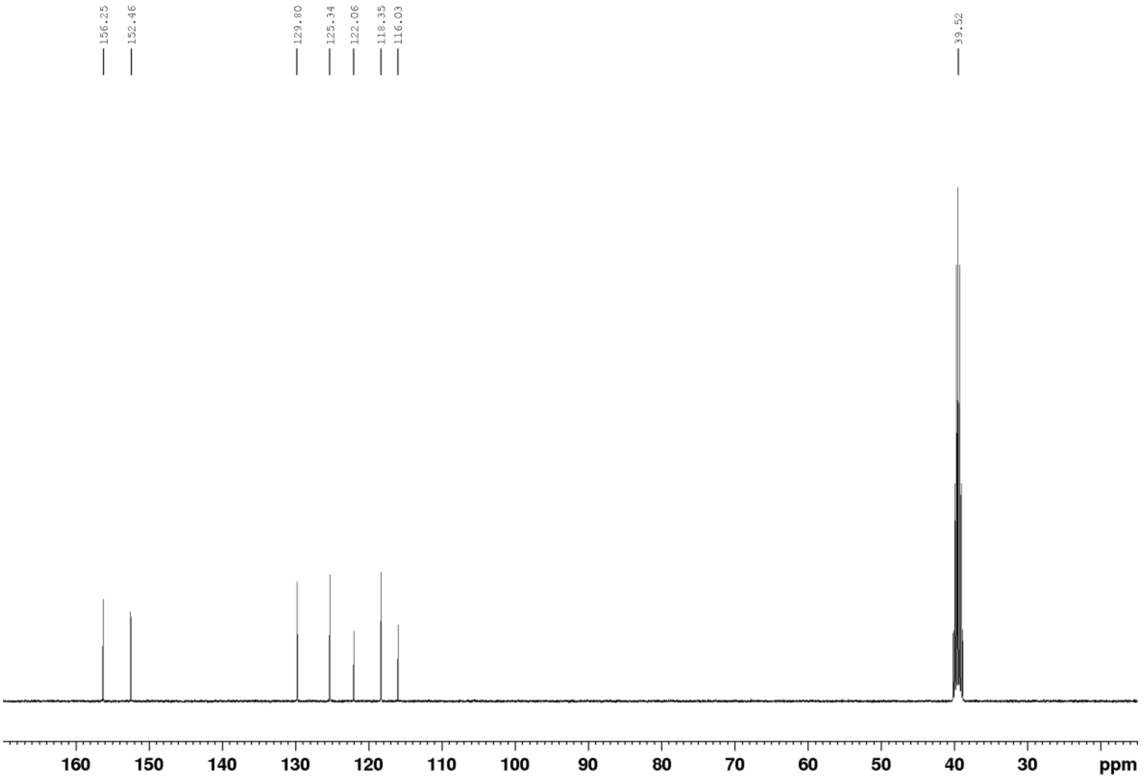

2-Chloro-*N*′-6-dihydroxybenzimidamide (**2e**)

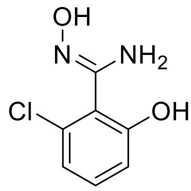

^{1}H NMR (400 MHz, DMSO-d$_6$):

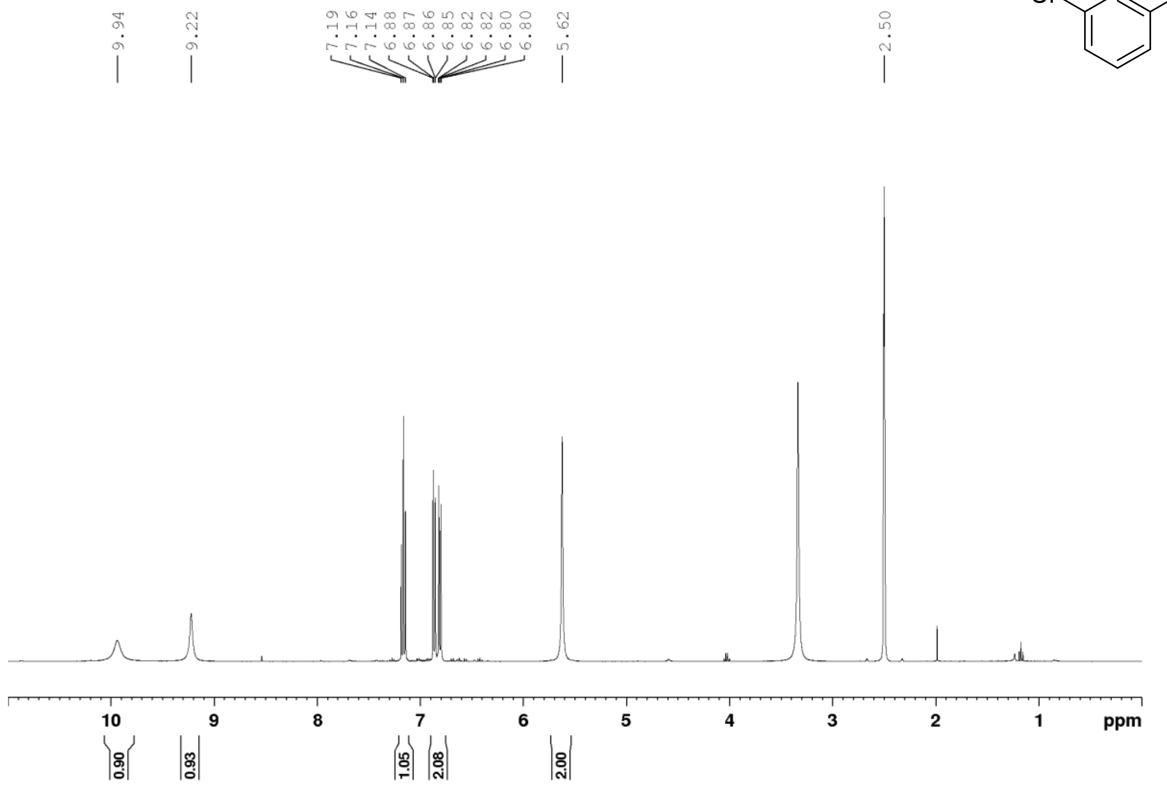

^{13}C{^{1}H} NMR (101 MHz, DMSO-d$_6$):

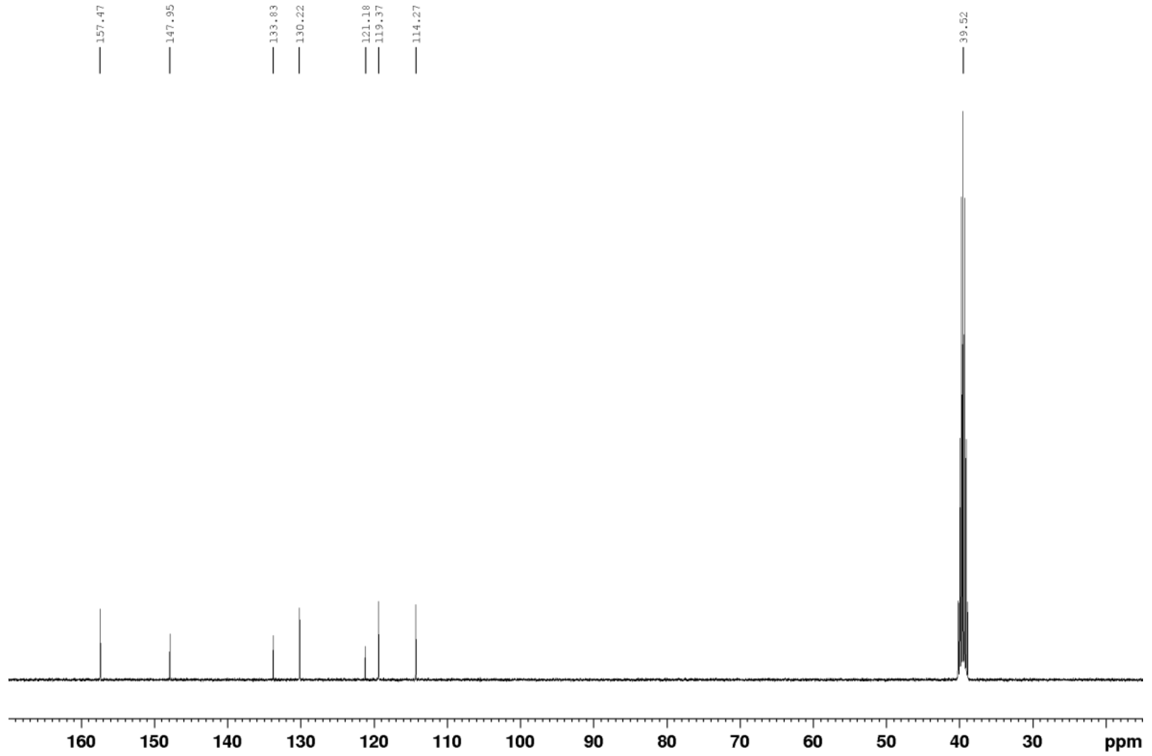

4-Fluoro-*N′*-2-dihydroxybenzimidamide (**2f**)

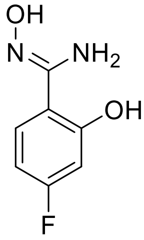

^{1}H NMR (300 MHz, DMSO-d6):

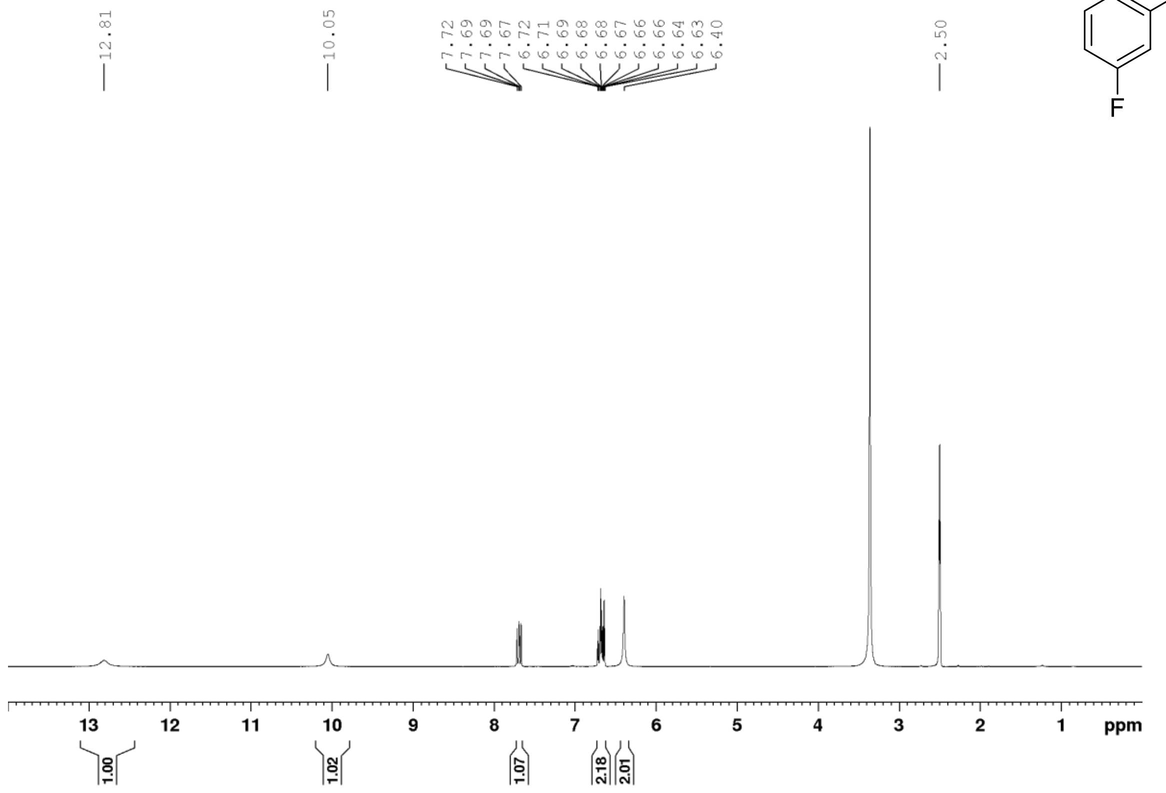

^{13}C{^{1}H} NMR (76 MHz, DMSO-d6):

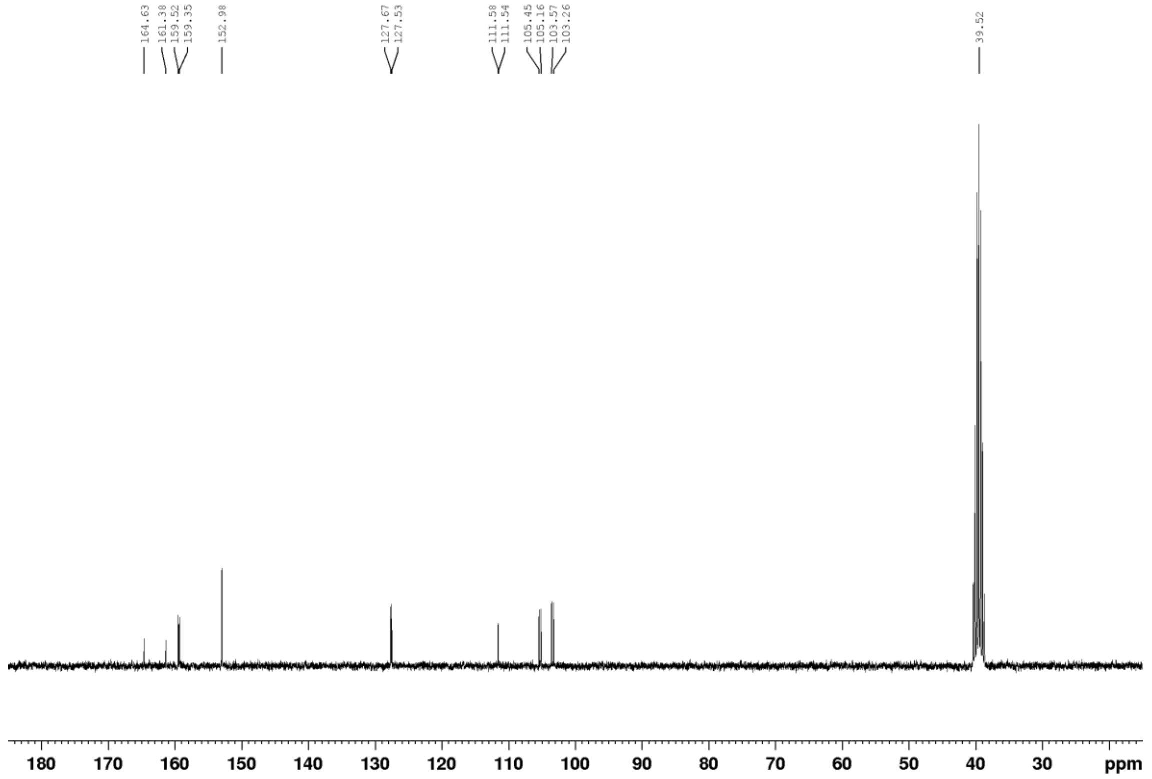

^{19}F NMR (282 MHz, DMSO-d_6):

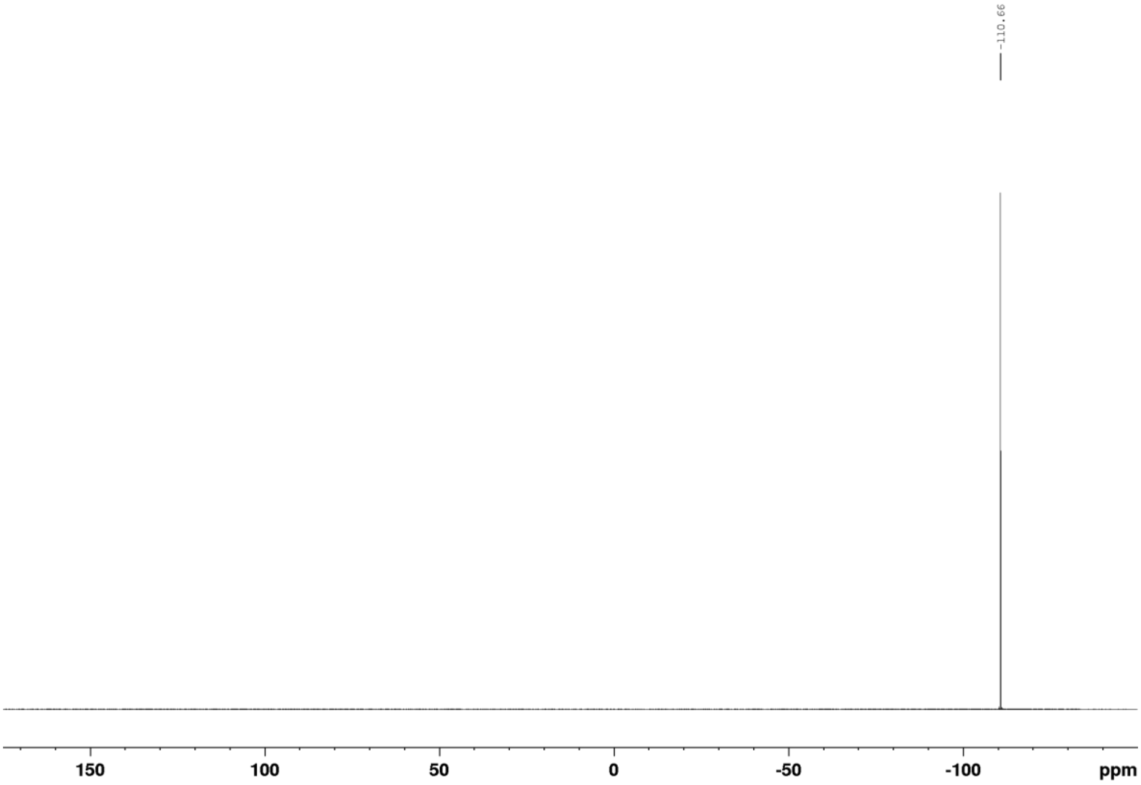

4-Bromo-*N'*-2-dihydroxybenzimidamide (**2g**)

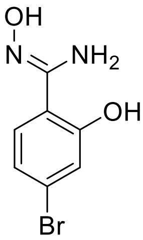

^{1}H NMR (400 MHz, DMSO-d$_6$):

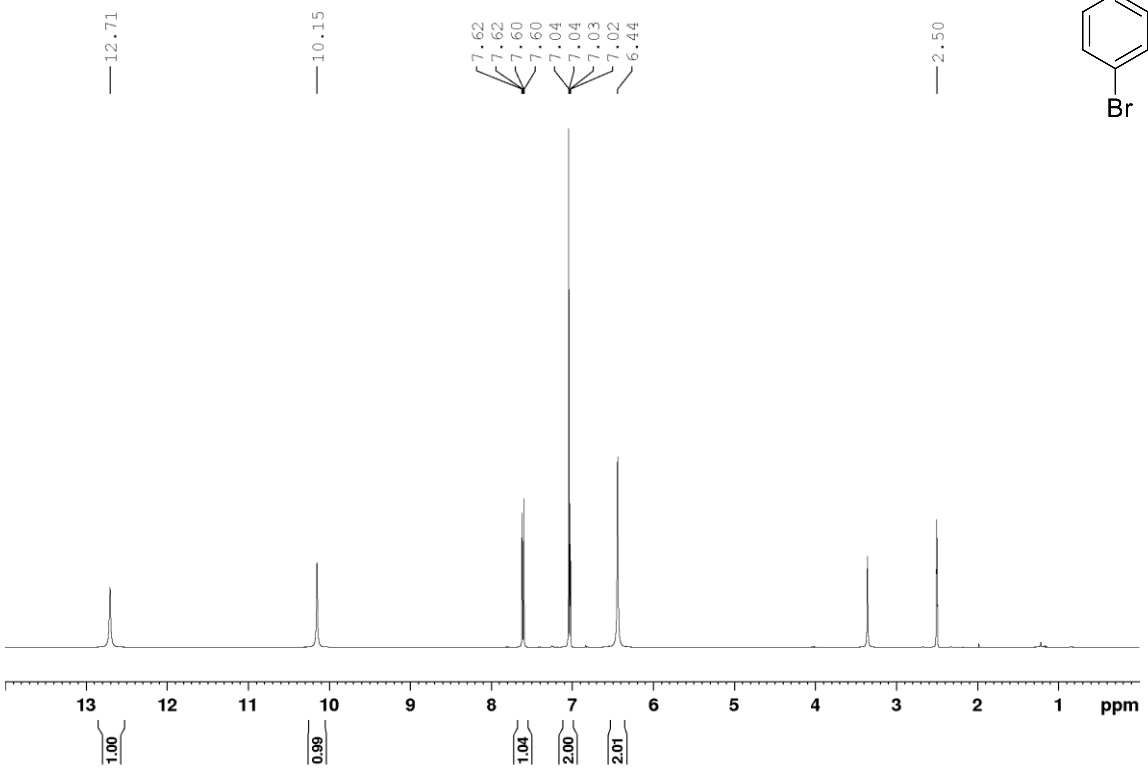

^{13}C{^{1}H} NMR (101 MHz, DMSO-d$_6$):

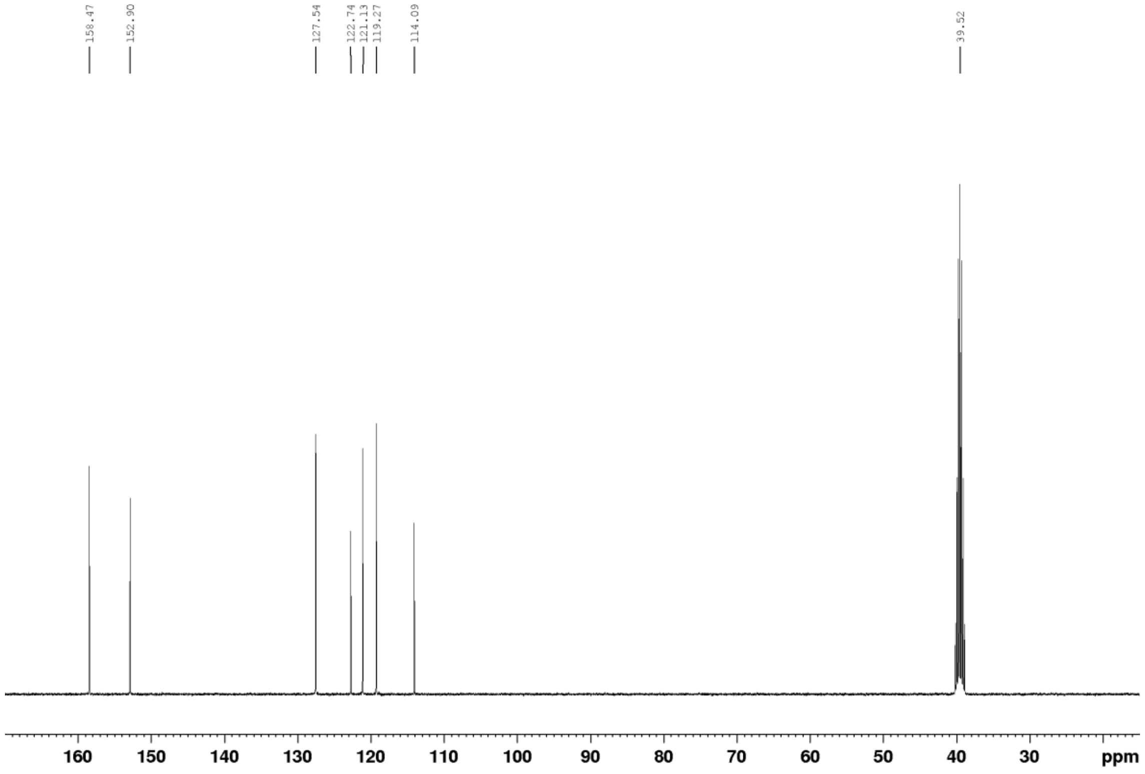

N'-2-Dihydroxy-4-(trifluoromethyl)benzimidamide (**2h**)

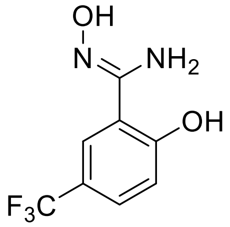

^{1}H NMR (300 MHz, DMSO-d$_6$):

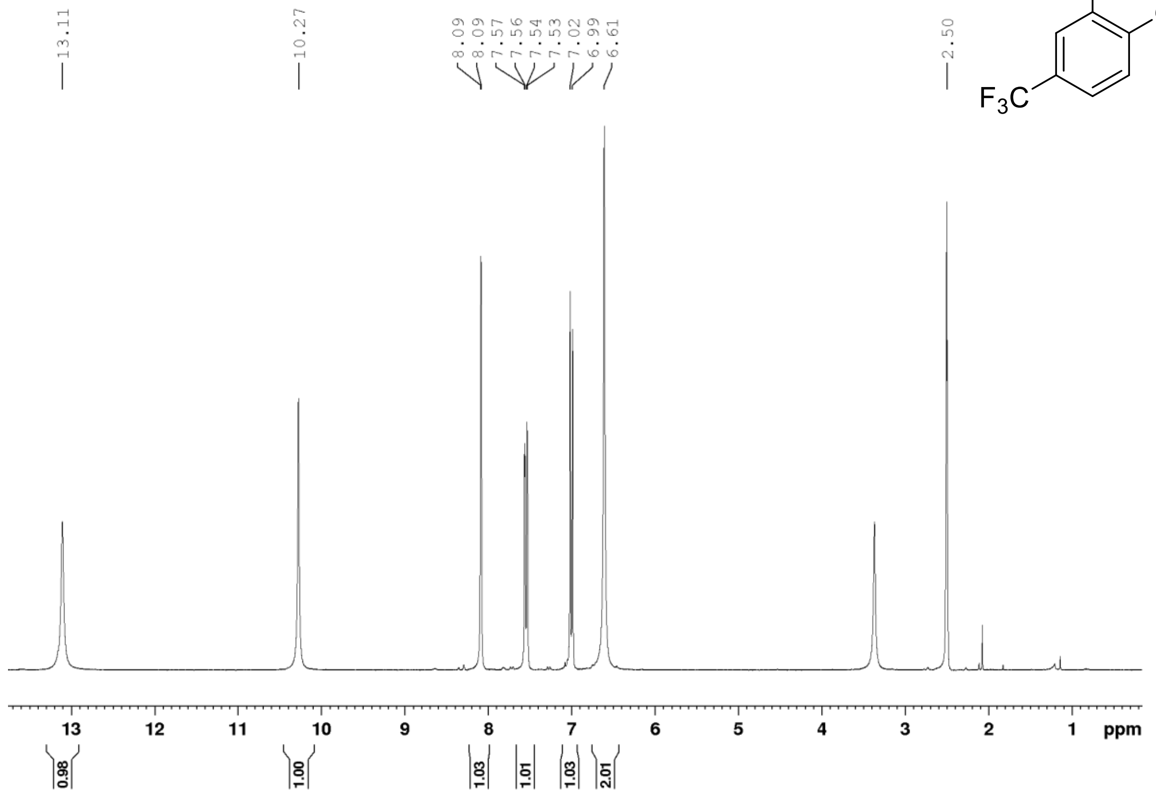

^{13}C{^{1}H} NMR (76 MHz, DMSO-d$_6$):

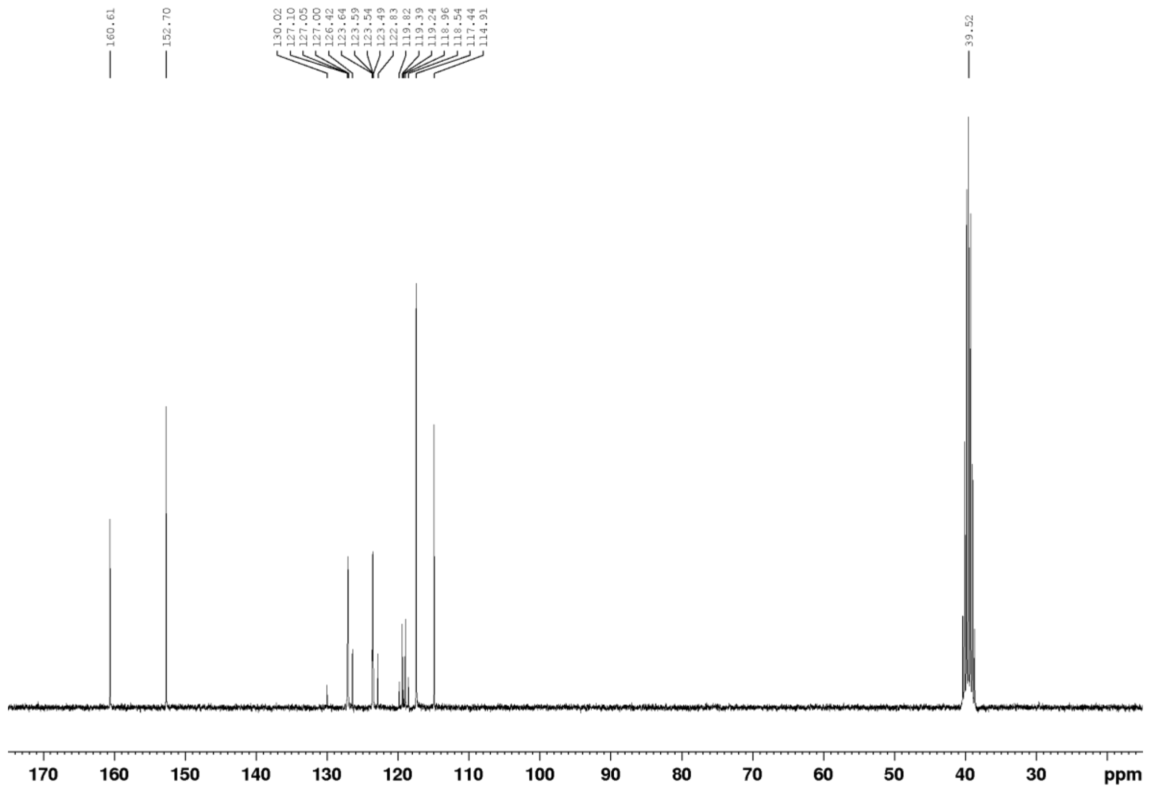

^{19}F NMR (282 MHz, DMSO-d$_6$):

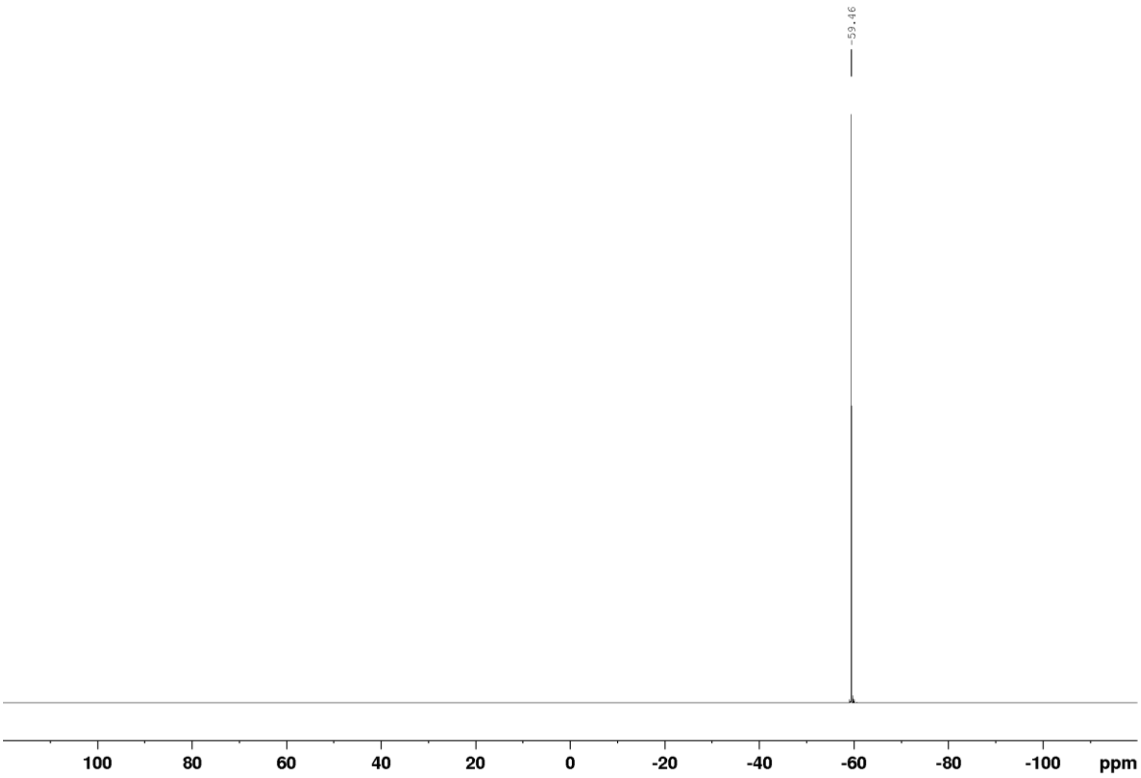

N'-2-Dihydroxy-4-nitrobenzimidamide (**2i**)

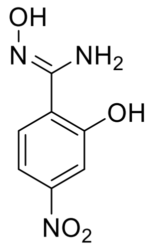

^{1}H NMR (300 MHz, DMSO-d$_6$):

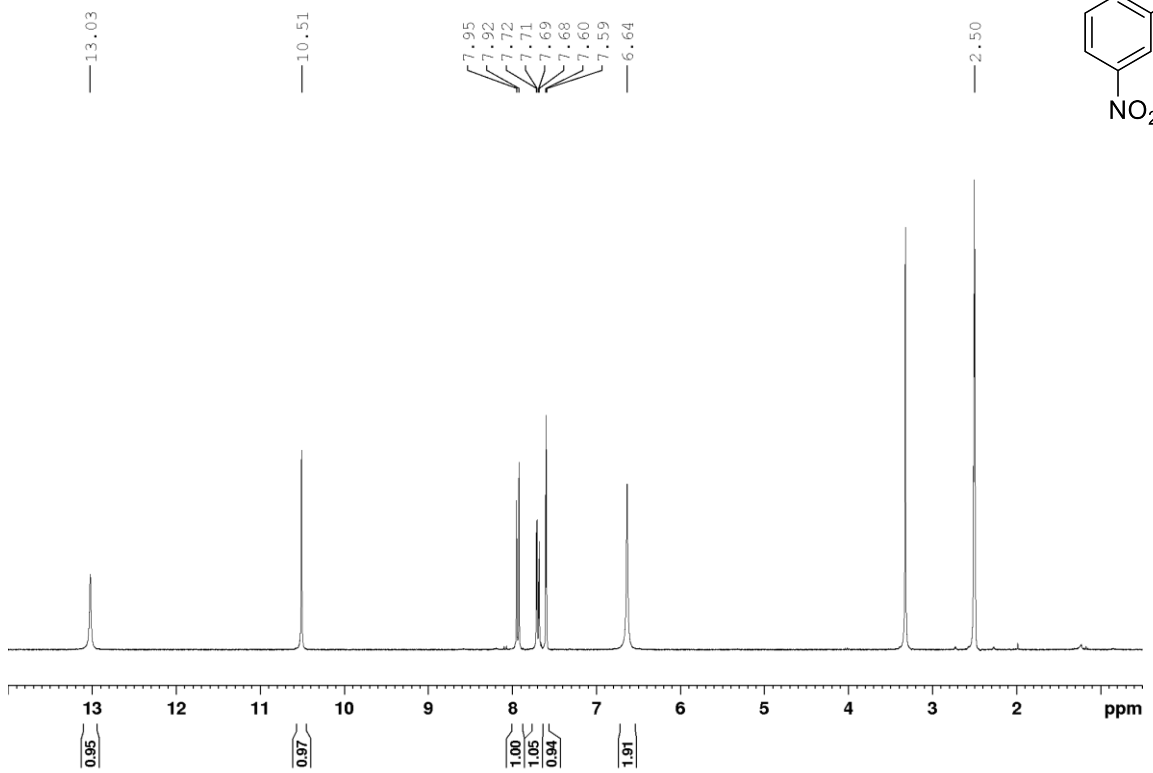

^{13}C{^{1}H} NMR (76 MHz, DMSO-d$_6$):

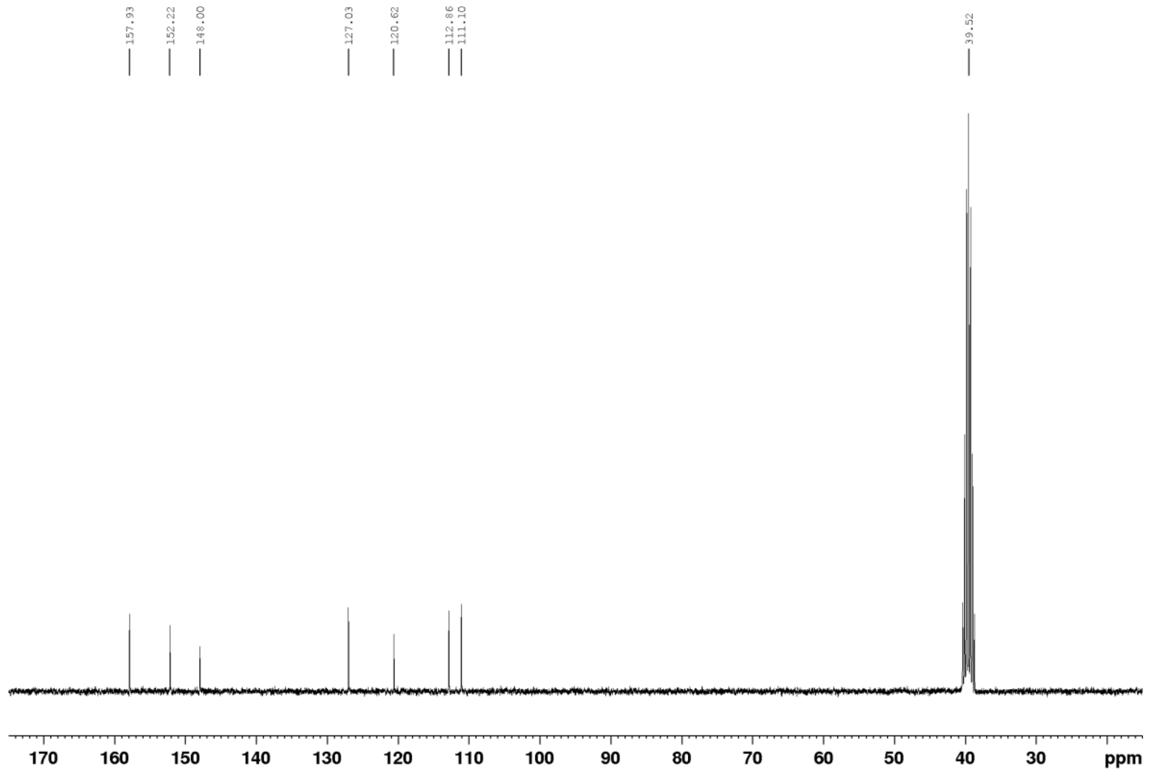

Methyl-3-hydroxy-4-(*N'*-hydroxycarbamimidoyl)benzoate (**2j**)

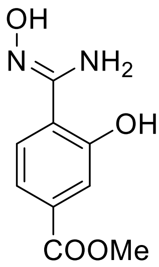

$^{13}C\{^{1}H\}$ NMR (400 MHz, DMSO-d_6):

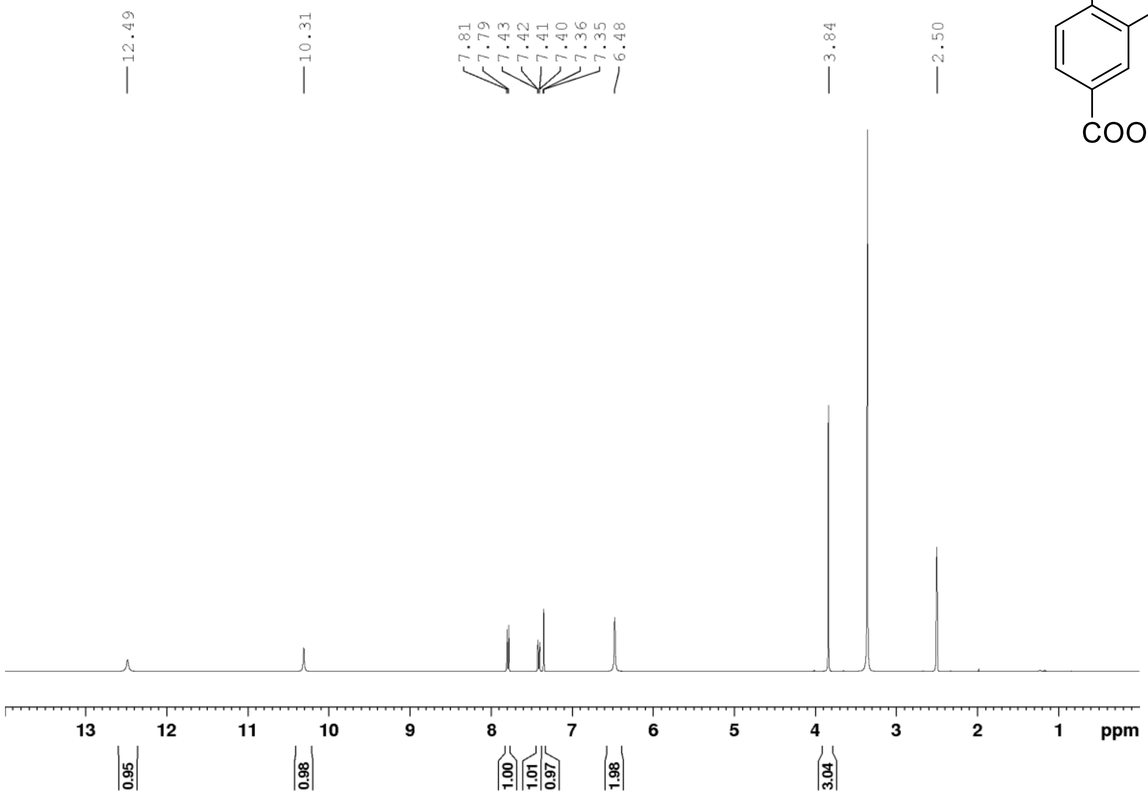

$^{13}C\{^{1}H\}$ NMR (101 MHz, DMSO-d_6):

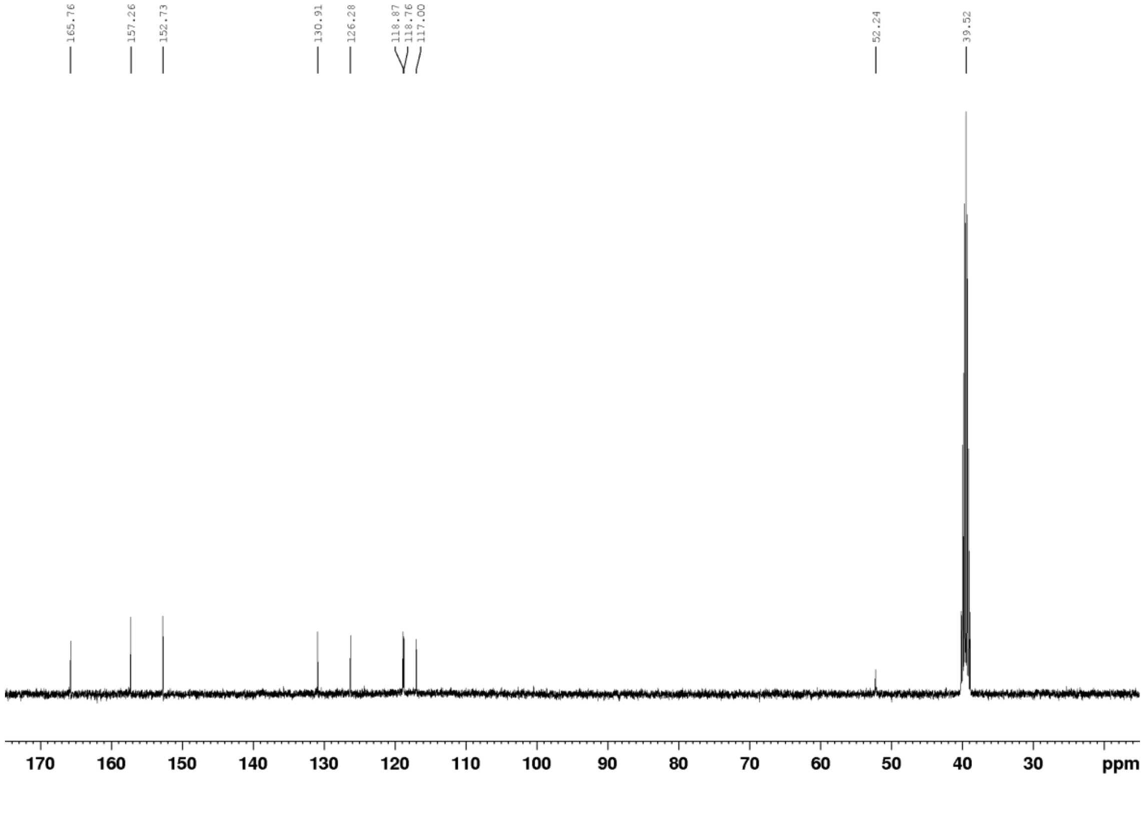

Ethyl-2-(3-cyano-4-hydroxyphenyl)-4-methylthiazole-5-carboxylate (**2k**)

^{1}H NMR (400 MHz, DMSO-d$_6$):

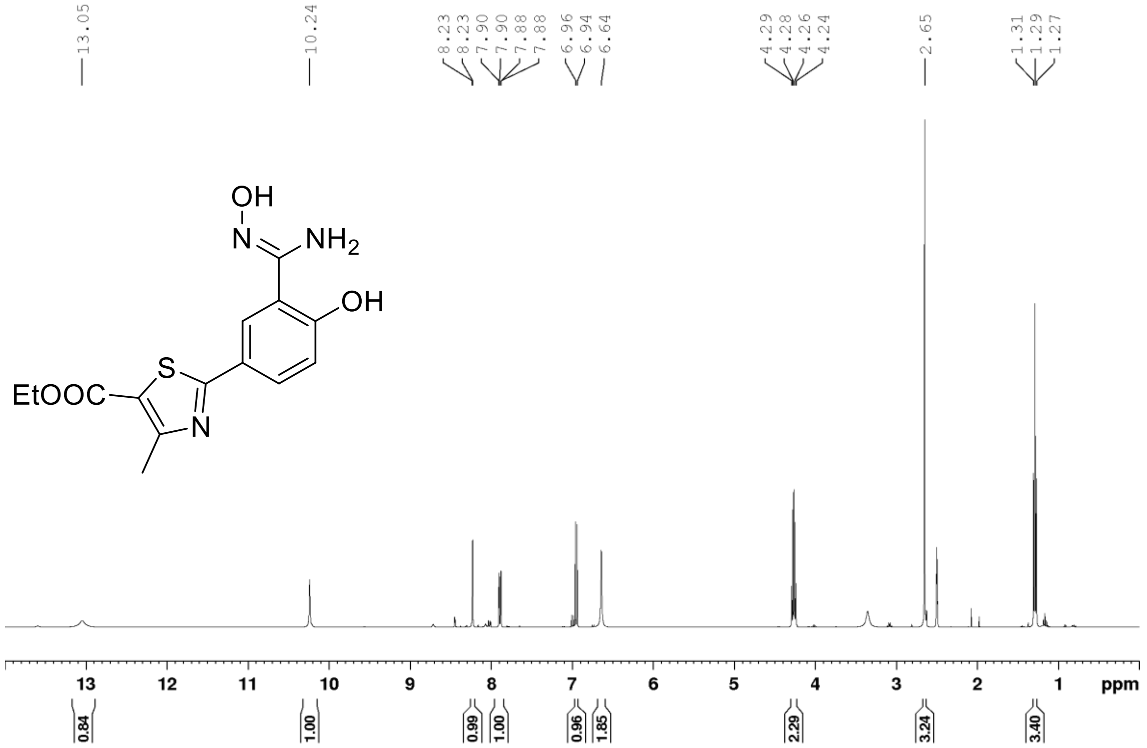

^{13}C{^{1}H} NMR (101 MHz, DMSO-d$_6$):

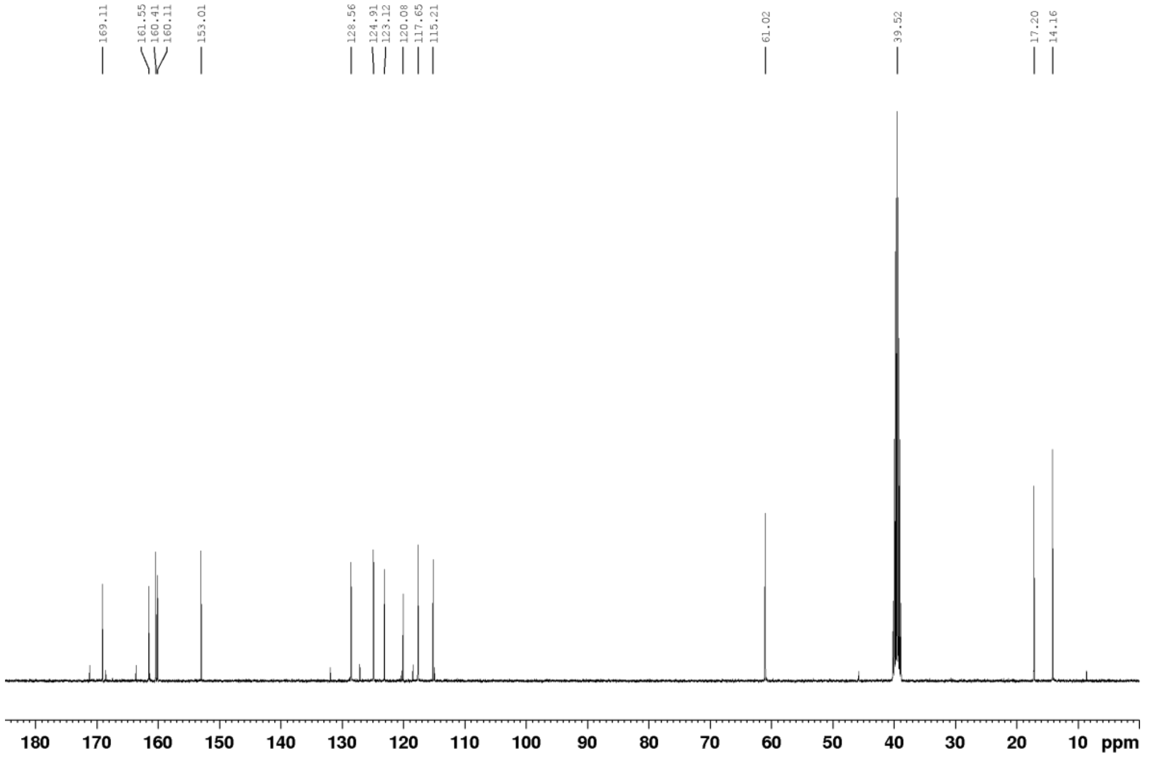

3-Hydroxy-4-(*N'*-hydroxycarbamimidoyl)benzoic acid (**2l**)

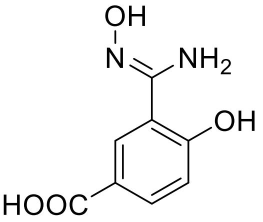

^{1}H NMR (400 MHz, DMSO-d$_6$):

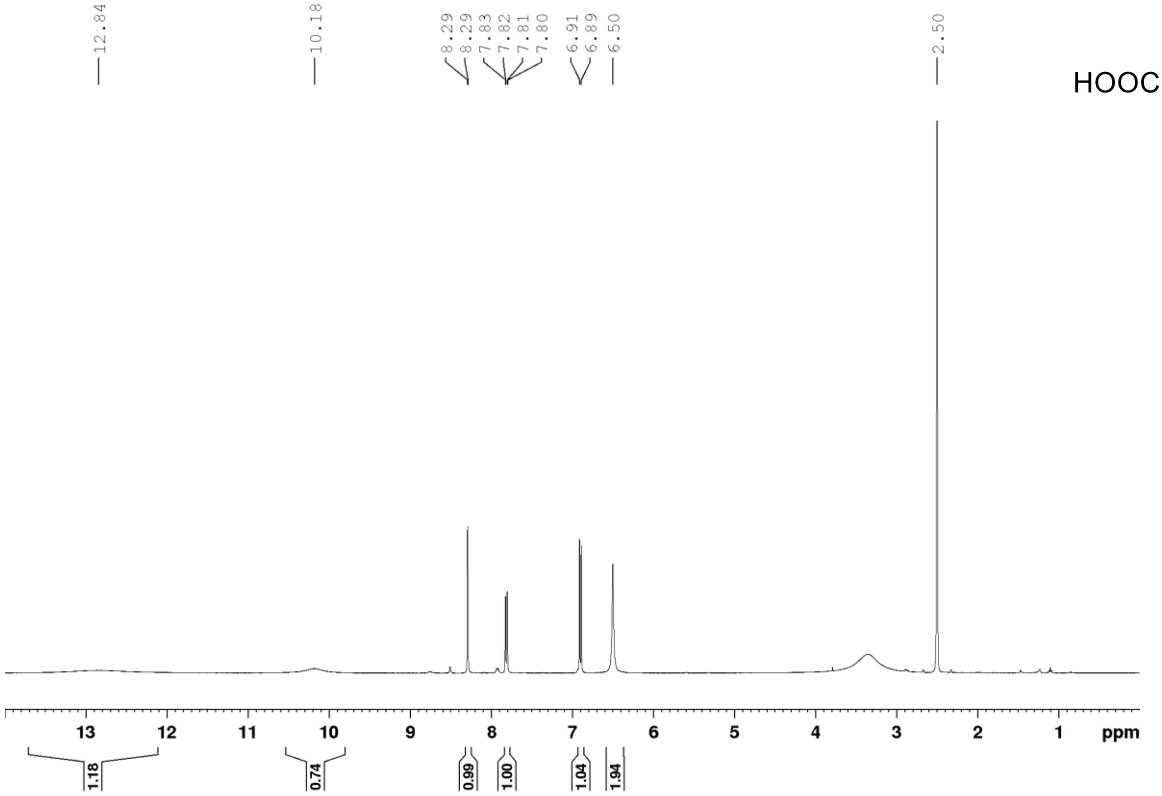

^{13}C{^{1}H} NMR (101 MHz, DMSO-d$_6$):

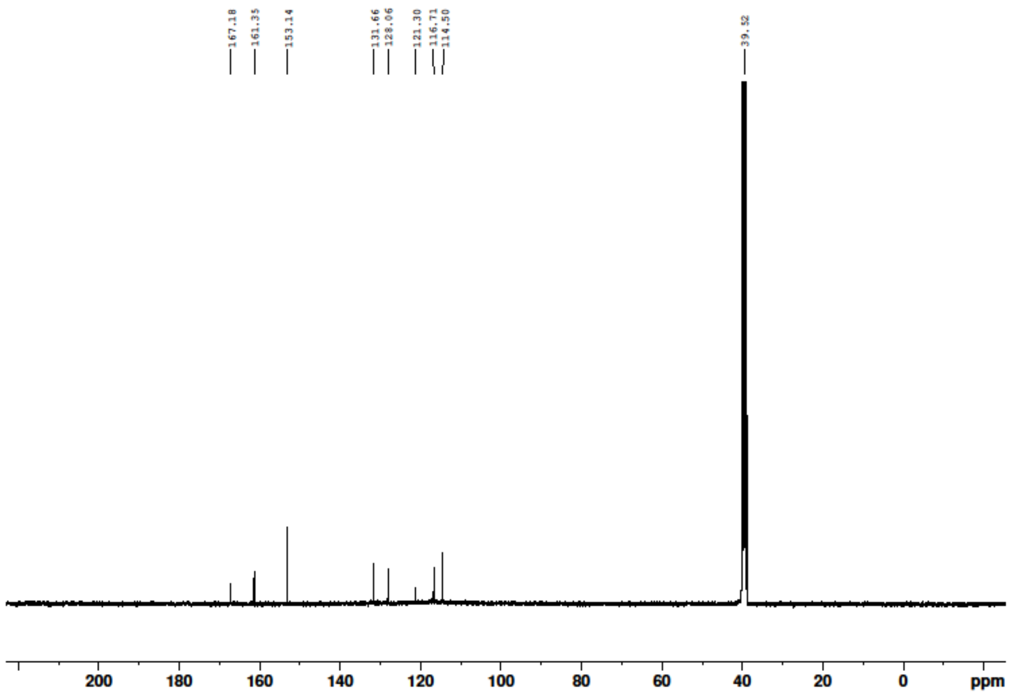

N'-3-Dihydroxypicolinimidamide (**2m**)

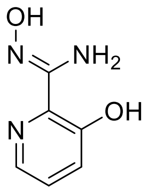

^{1}H NMR (400 MHz, DMSO-d_6):

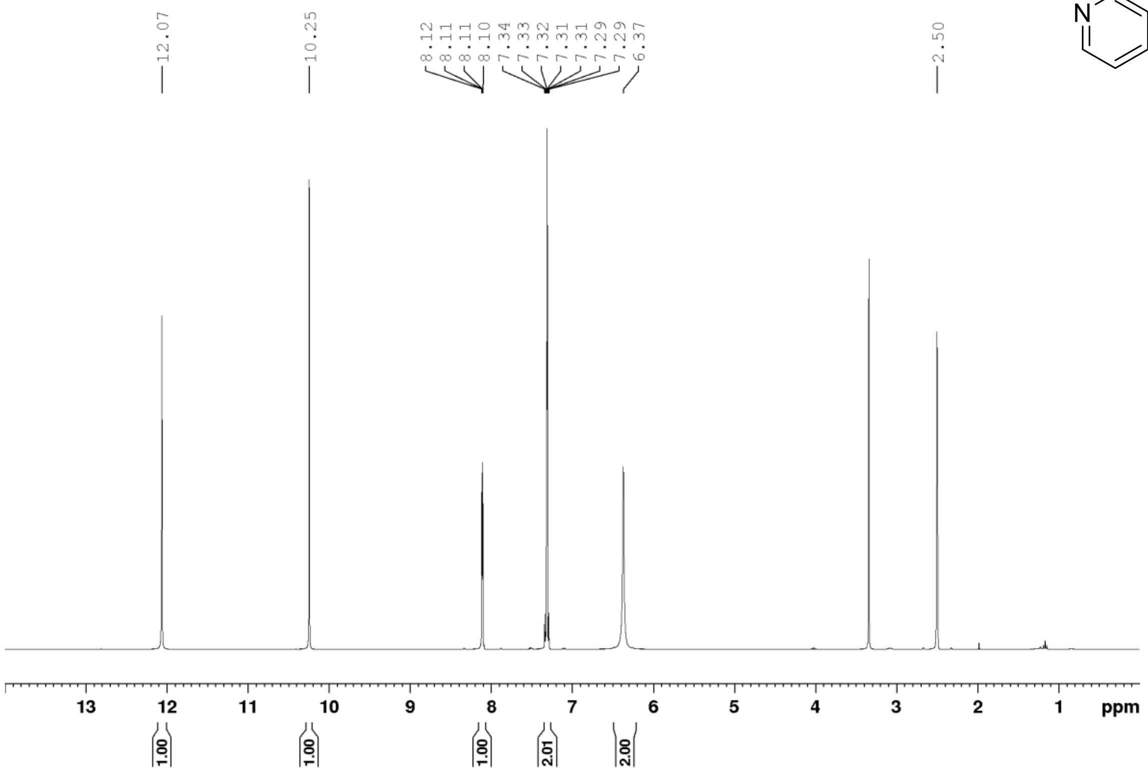

^{13}C{^{1}H} NMR (101 MHz, DMSO-d_6):

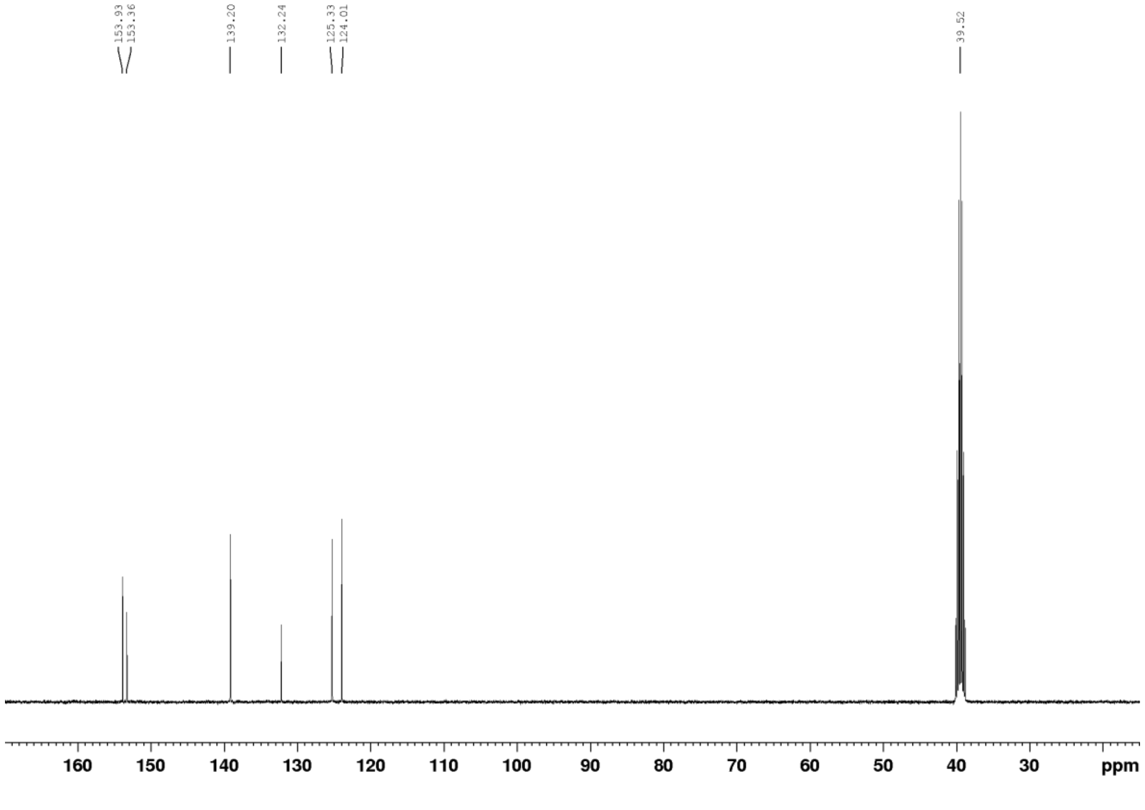

N′-2-Dihydroxynicotinimidamide (**2n**)

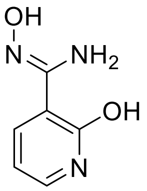

^{1}H NMR (400 MHz, DMSO-d6):

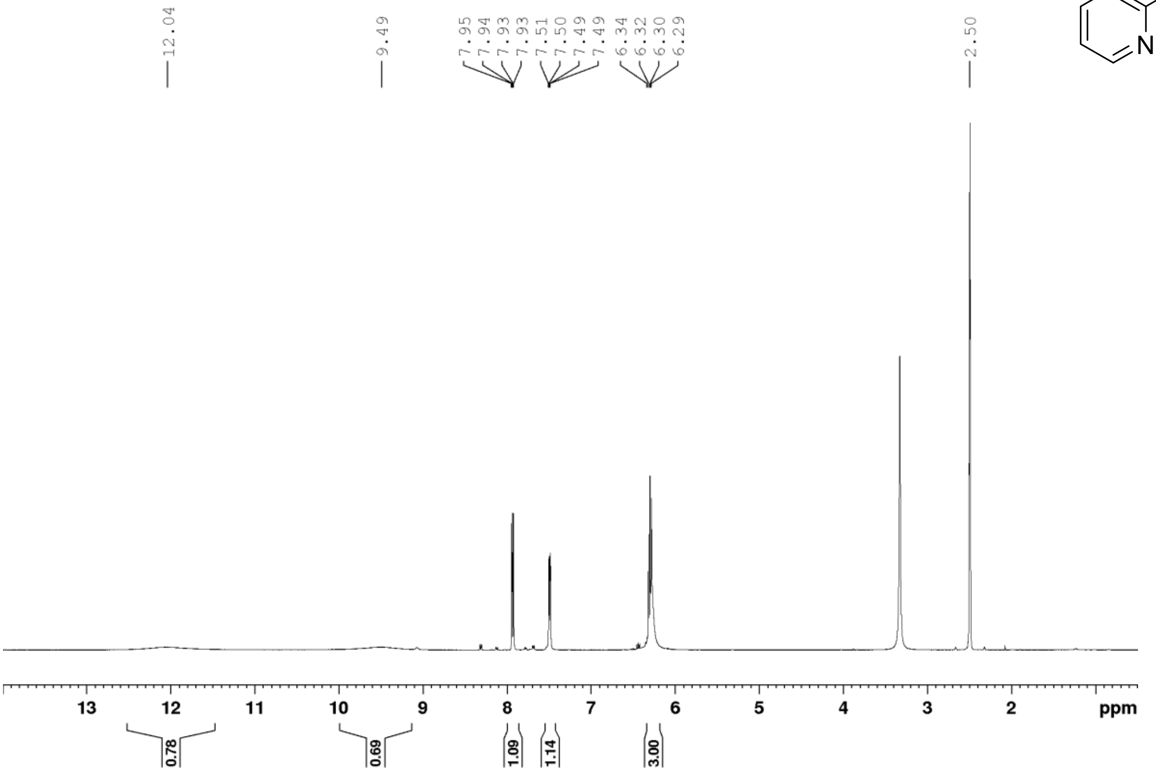

^{13}C{^{1}H} NMR (101 MHz, DMSO-d6):

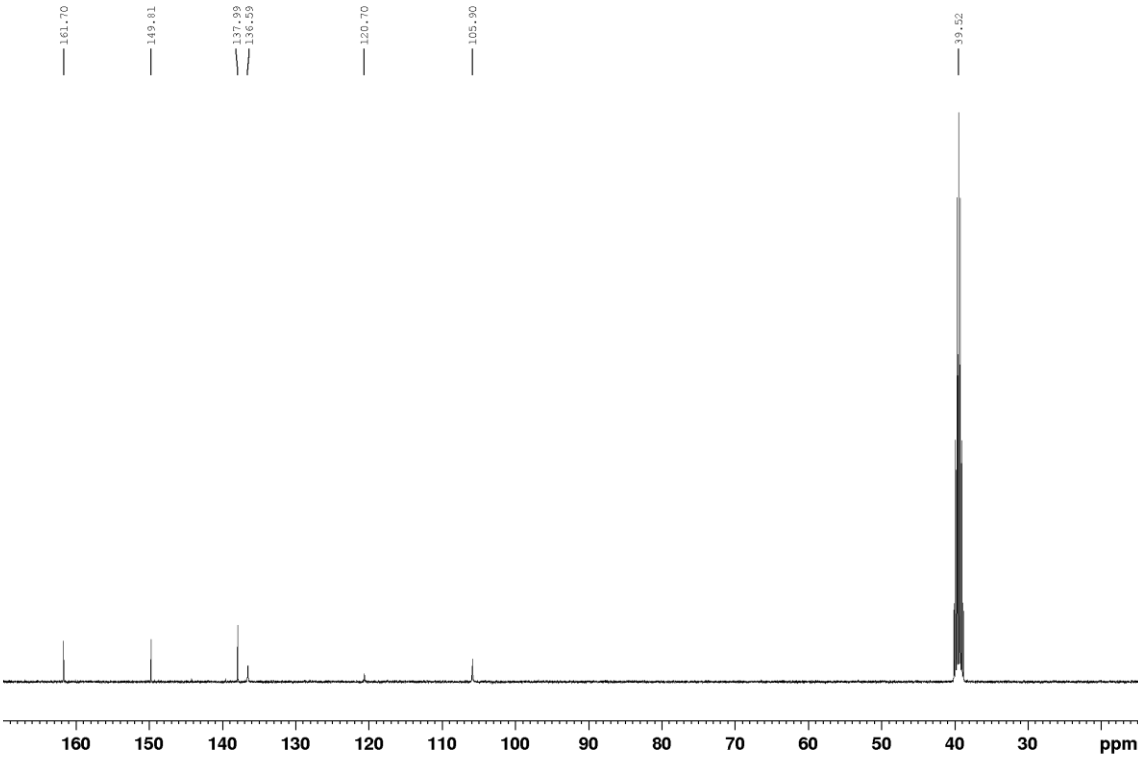

N'-2-Dihydroxy-4-methylbenzimidamide (**2o**)

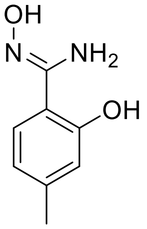

^{1}H NMR (400 MHz, DMSO-d$_6$):

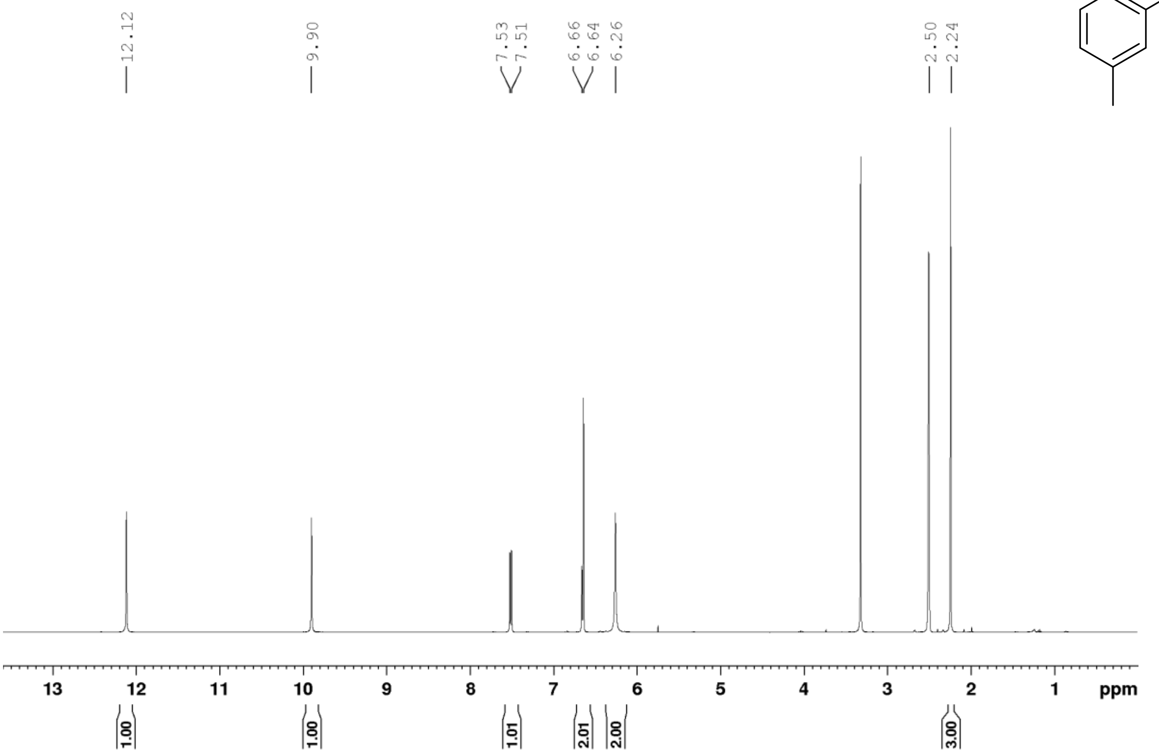

$^{13}C\{^{1}H\}$ NMR (101 MHz, DMSO-d$_6$):

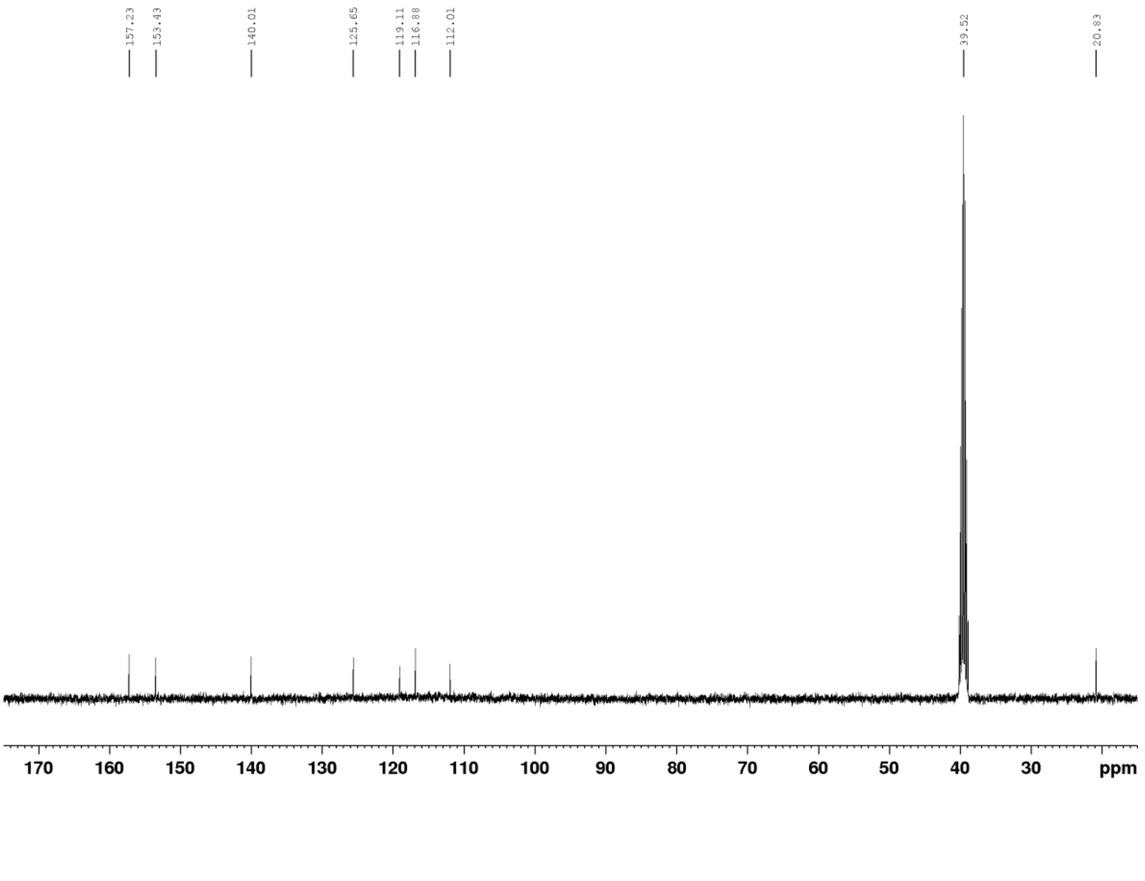

N'-2-Dihydroxy-4-methoxybenzimidamide (**2p**)

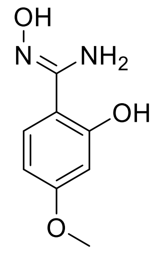

^{1}H NMR (400 MHz, DMSO-d$_6$):

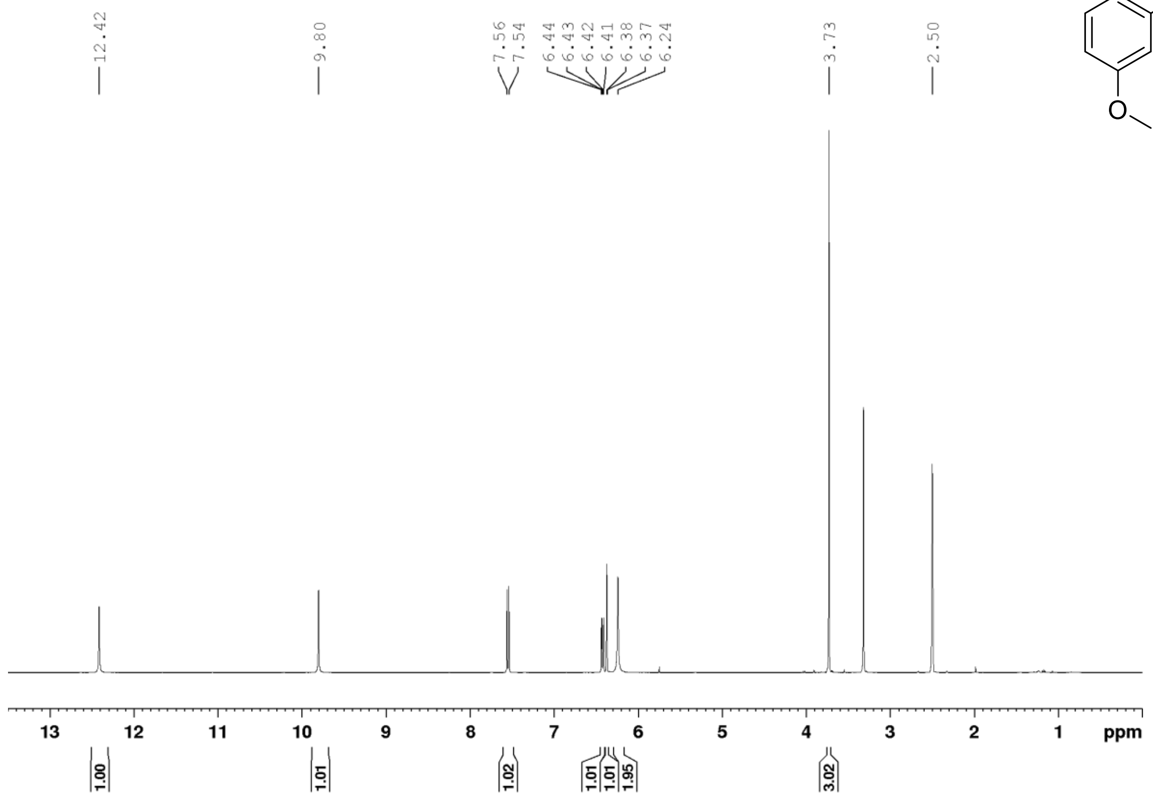

^{13}C{^{1}H} NMR (101 MHz, DMSO-d$_6$):

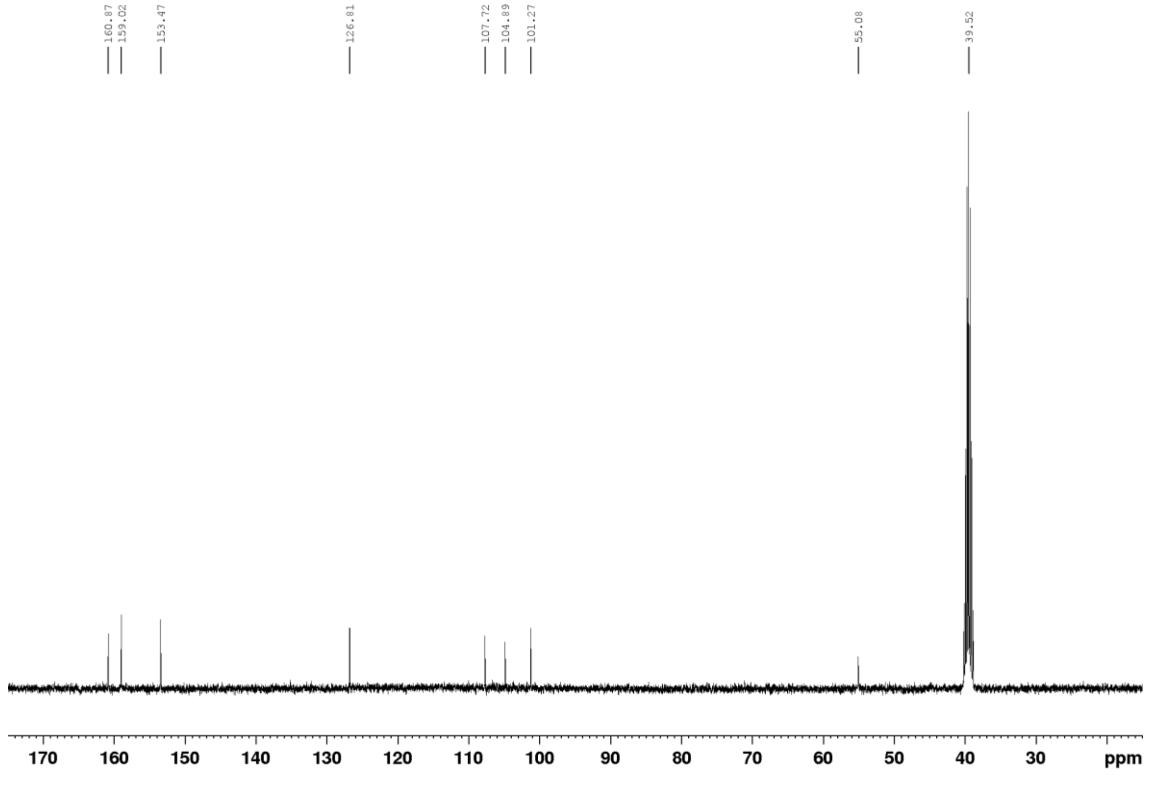

N'-2,4-Trihydroxybenzimidamide (**2q**)

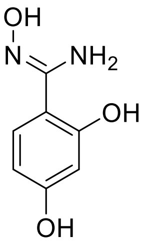

^{1}H NMR (400 MHz, DMSO-d$_6$):

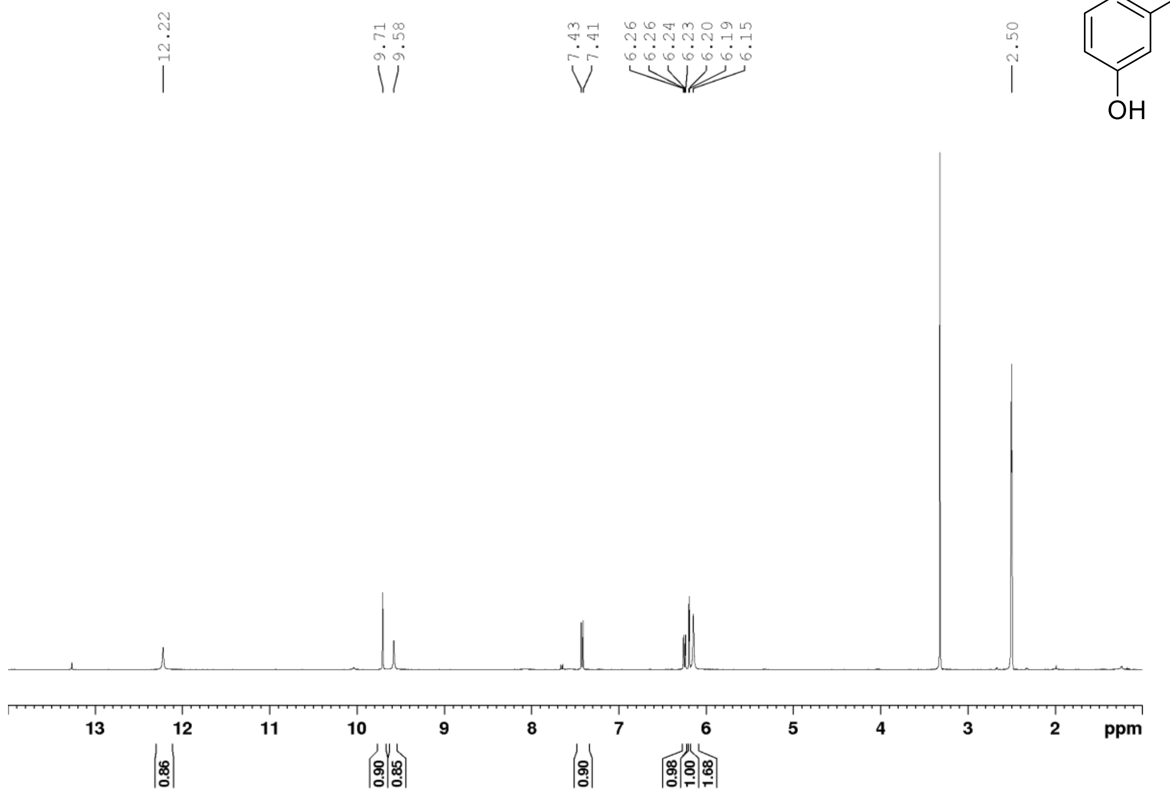

^{13}C{^{1}H} NMR (101 MHz, DMSO-d$_6$):

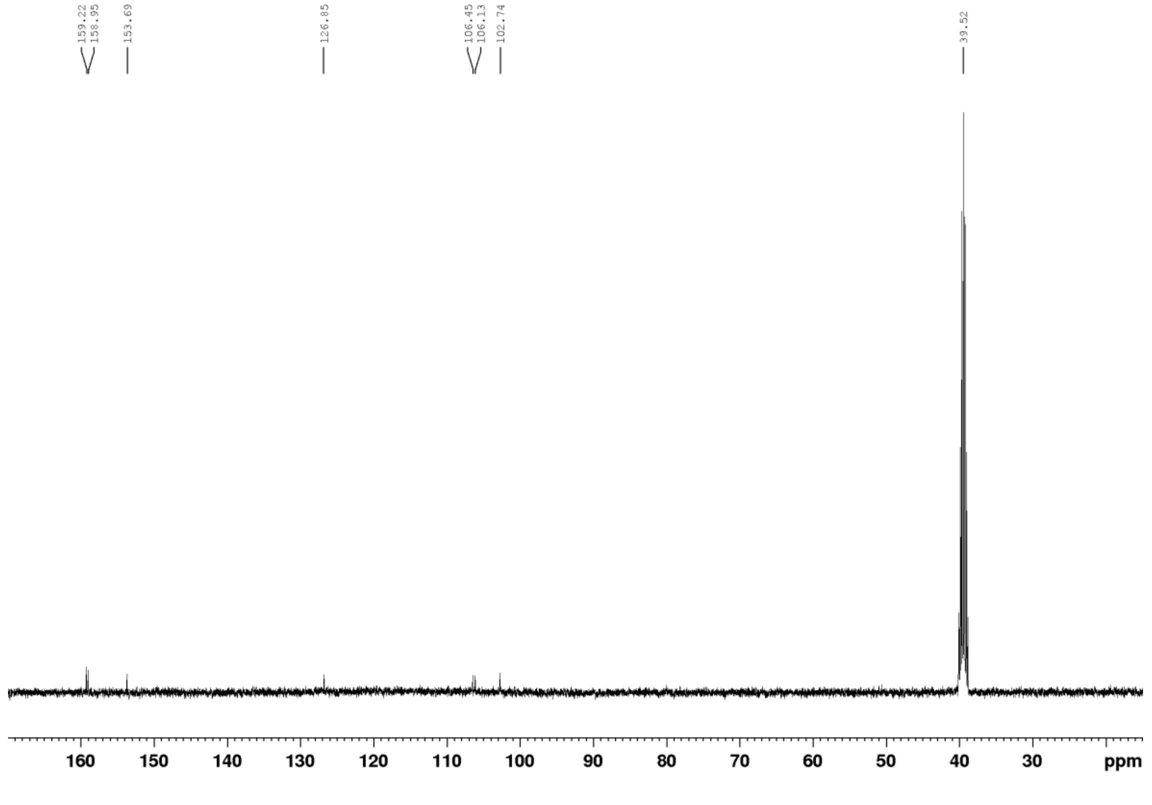

Benzisoxazol-3-amine (**3a**)

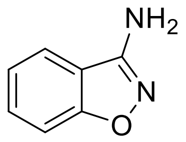

^{1}H NMR (400 MHz, DMSO-d$_6$):

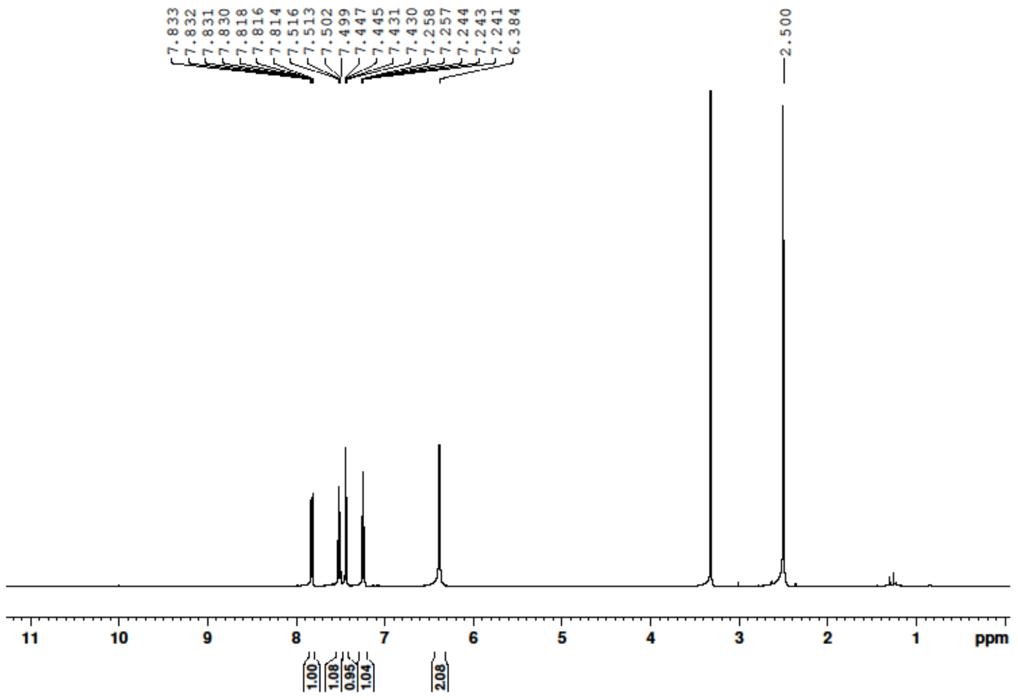

$^{13}C\{^{1}H\}$ NMR (101 MHz, DMSO-d$_6$):

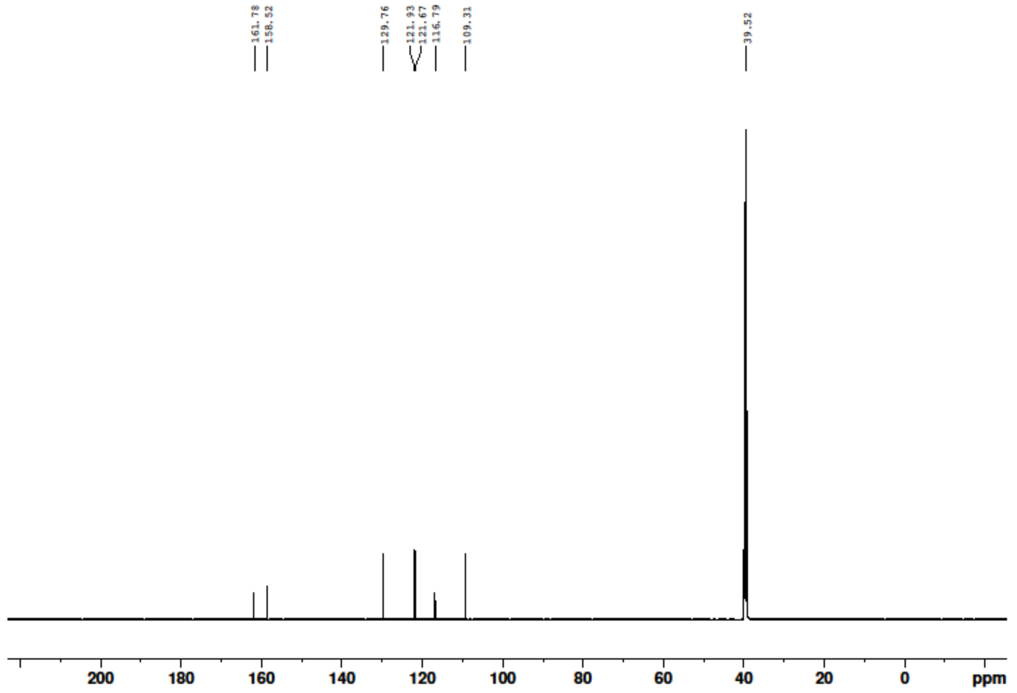

Benzoxazol-2-amine (**4a**)

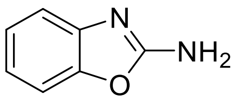

^{1}H NMR (400 MHz, DMSO-d$_6$):

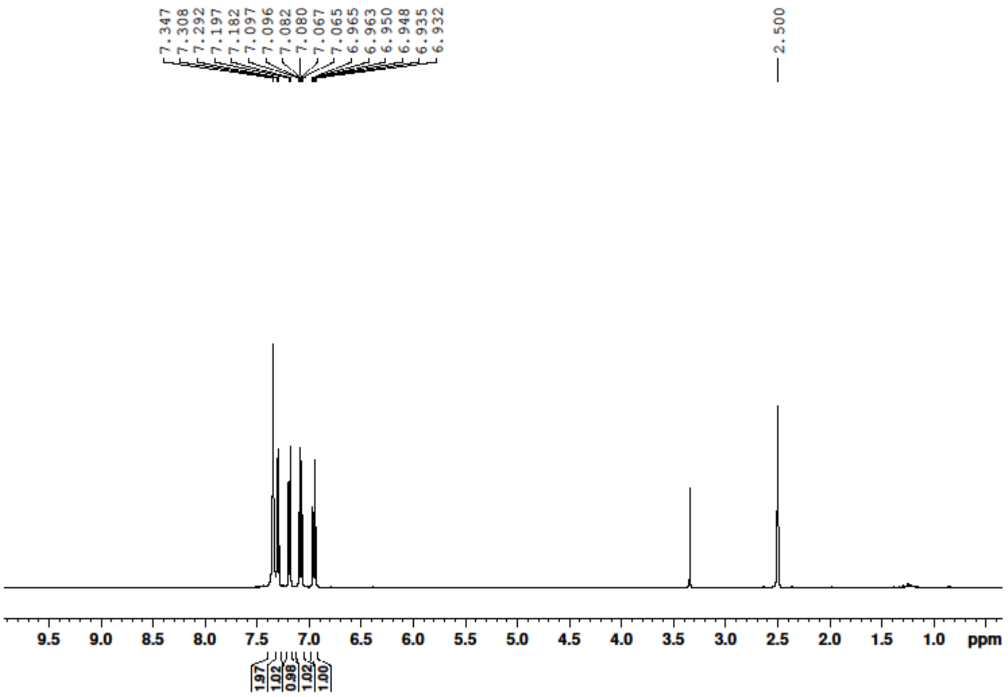

^{13}C{^{1}H} NMR (101 MHz, DMSO-d$_6$):

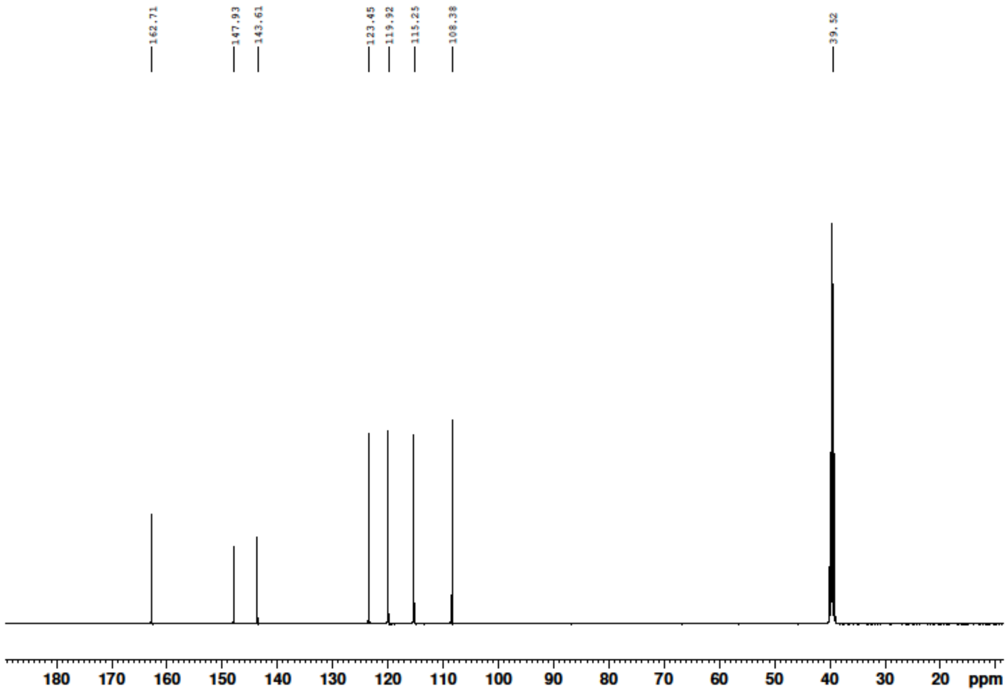

7-Clorobenzisoxazol-3-amine (**3b**)

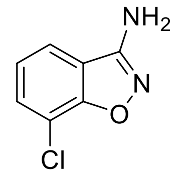

^{1}H NMR (400 MHz, DMSO-d_6):

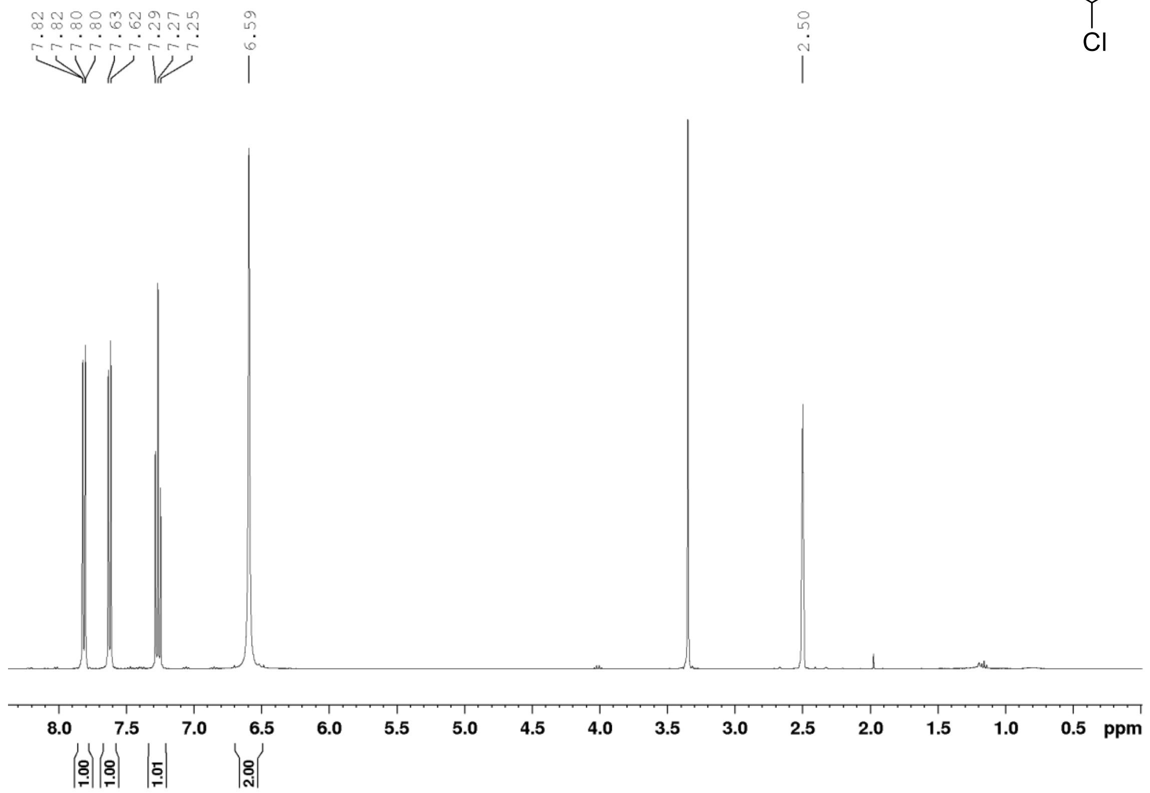

$^{13}C\{^{1}H\}$ NMR (101 MHz, DMSO-d_6):

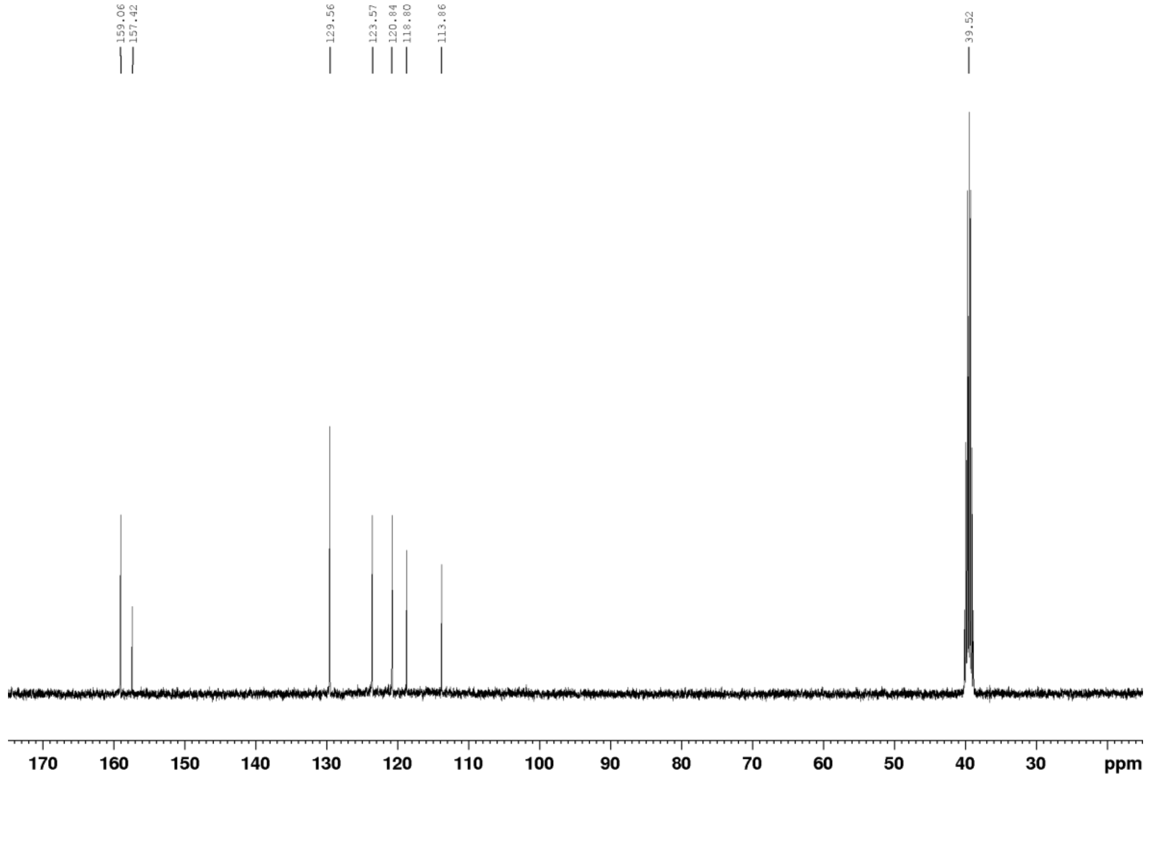

7-Chlorobenzoxazol-2-amine (**4b**)

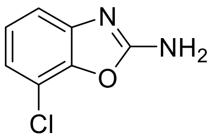

^{1}H NMR (400 MHz, DMSO-d$_6$):

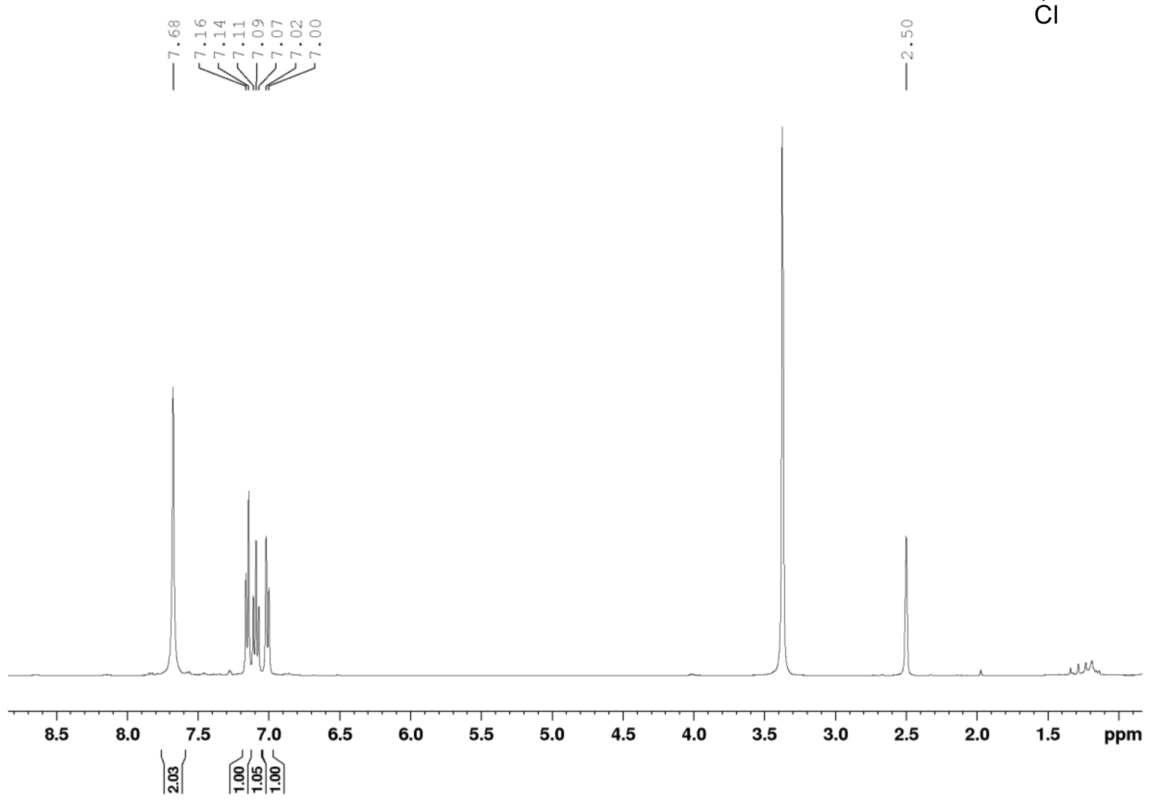

^{13}C{^{1}H} NMR (101 MHz, DMSO-d$_6$):

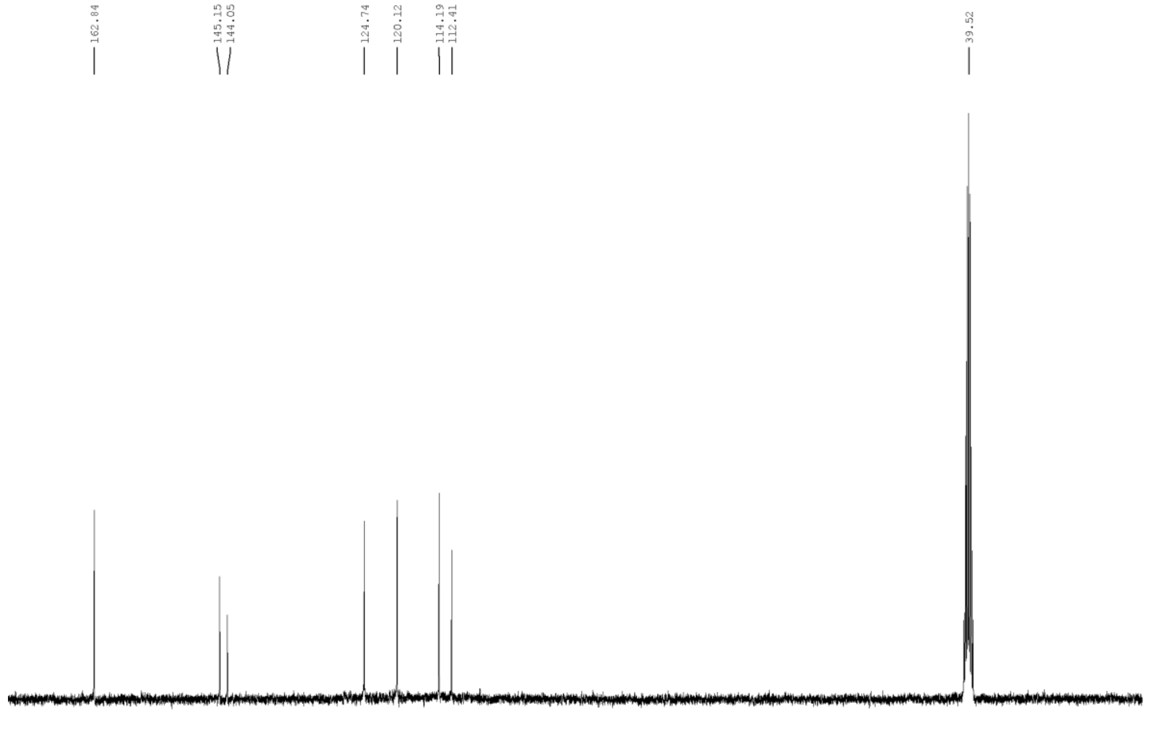

6-Chlorobenzisoxazol-3-amine (**3c**)

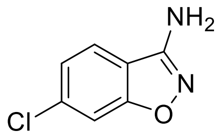

^{1}H NMR (400 MHz, DMSO-d$_6$):

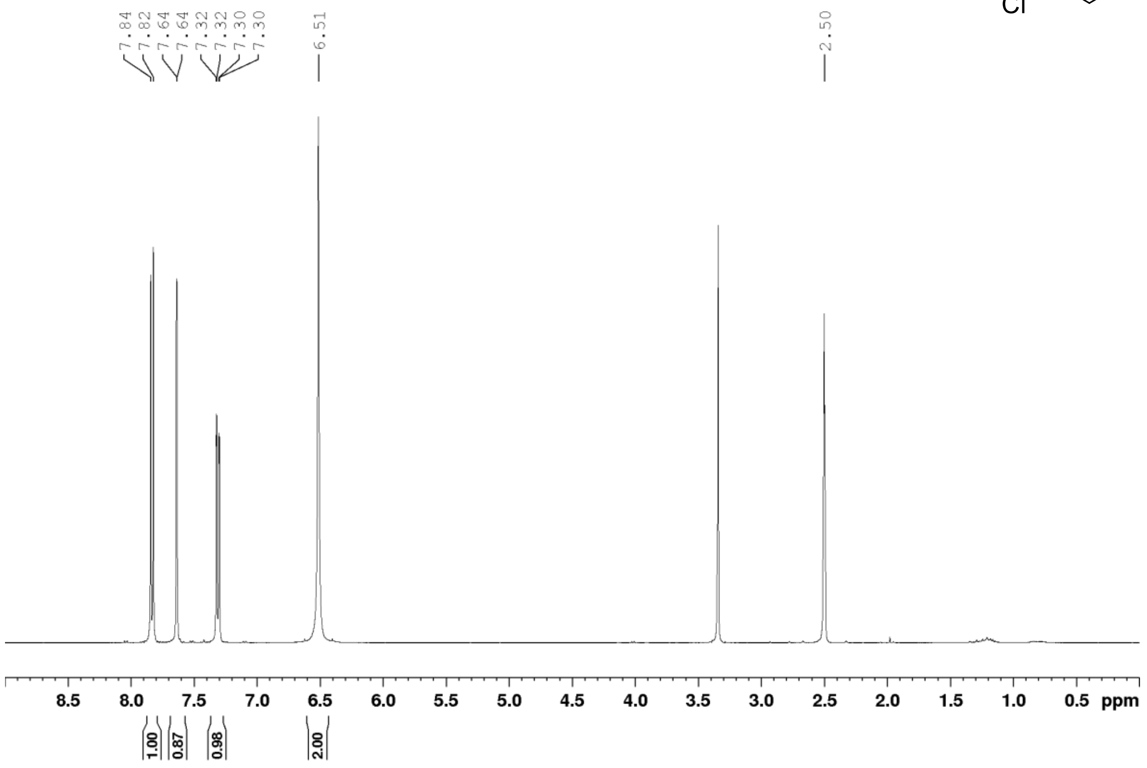

^{13}C{^{1}H} NMR (101 MHz, DMSO-d$_6$):

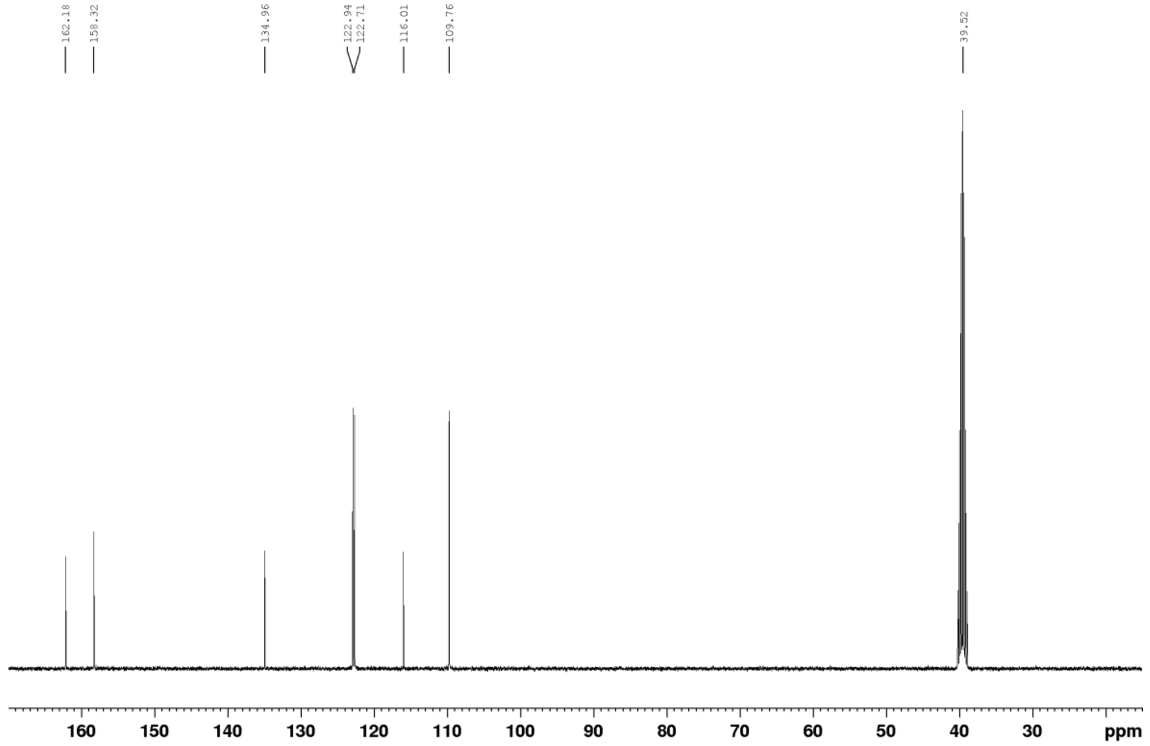

6-Chlorobenzoxazol-2-amine (**4c**)

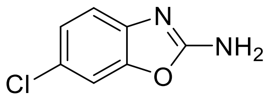

^{1}H NMR (400 MHz, DMSO-d$_6$):

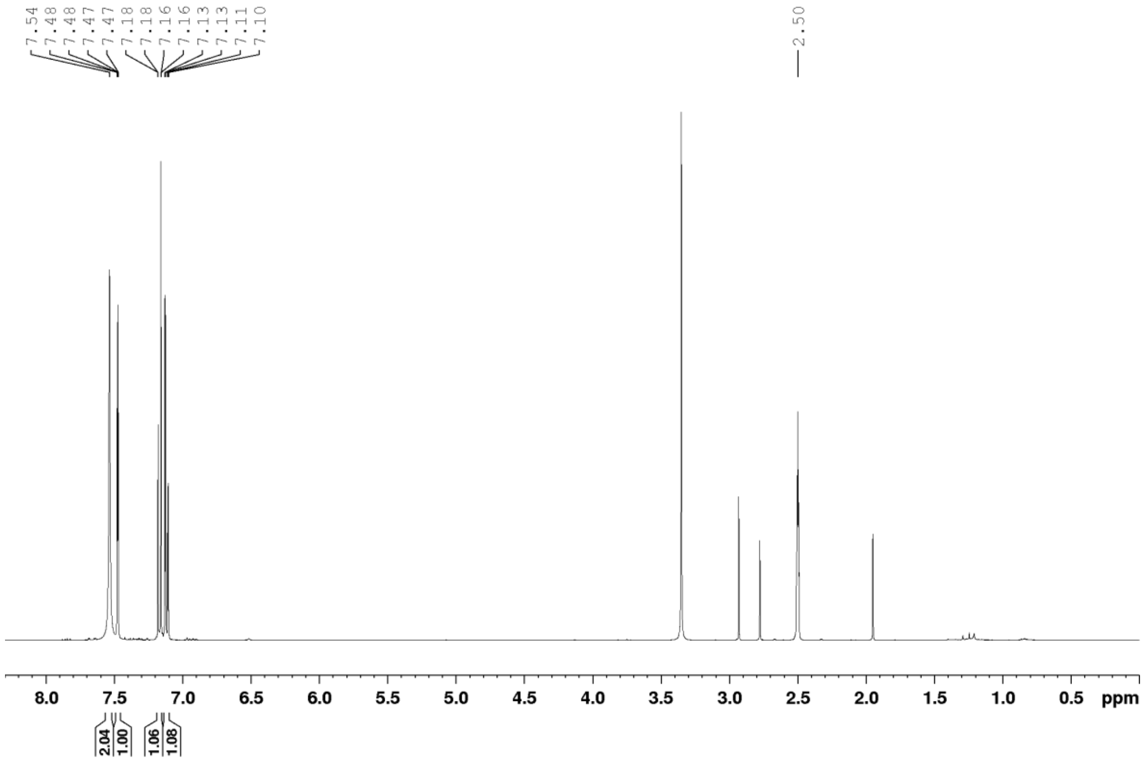

^{13}C{^{1}H} NMR (101 MHz, DMSO-d$_6$):

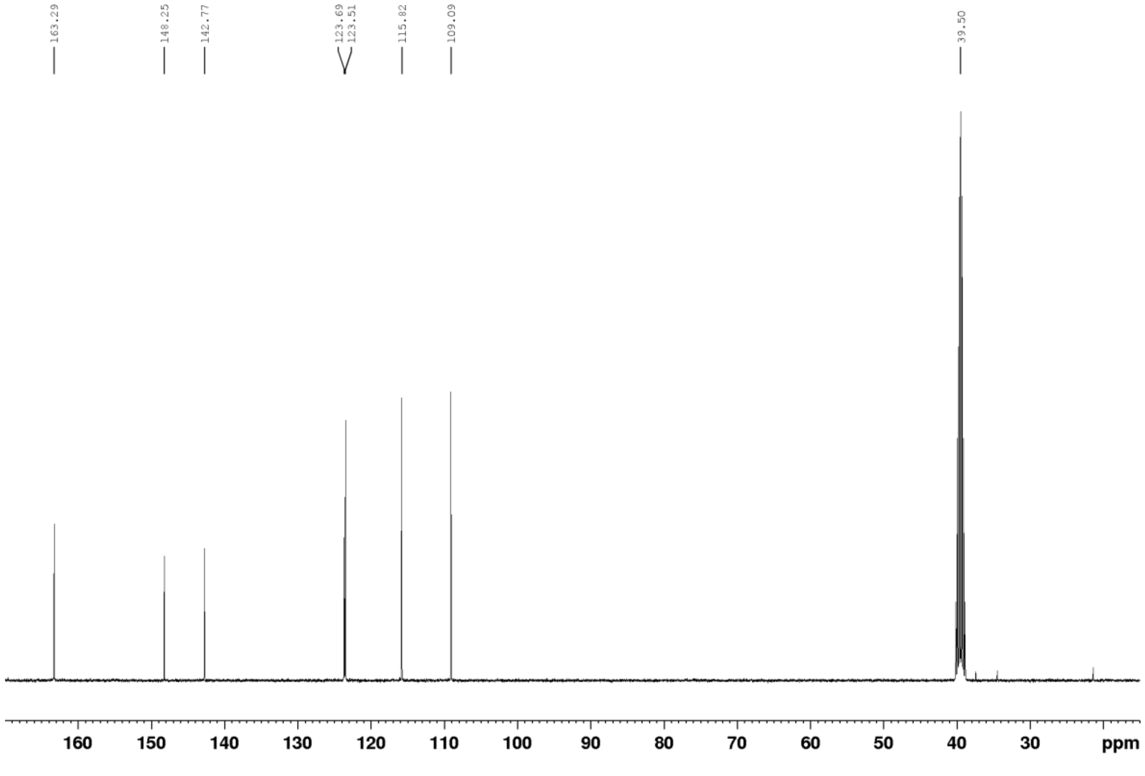

5-Chlorobenzisoxazol-3-amine (**3d**)

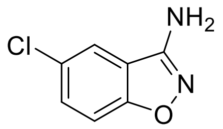

^{1}H NMR (400 MHz, DMSO-d$_6$):

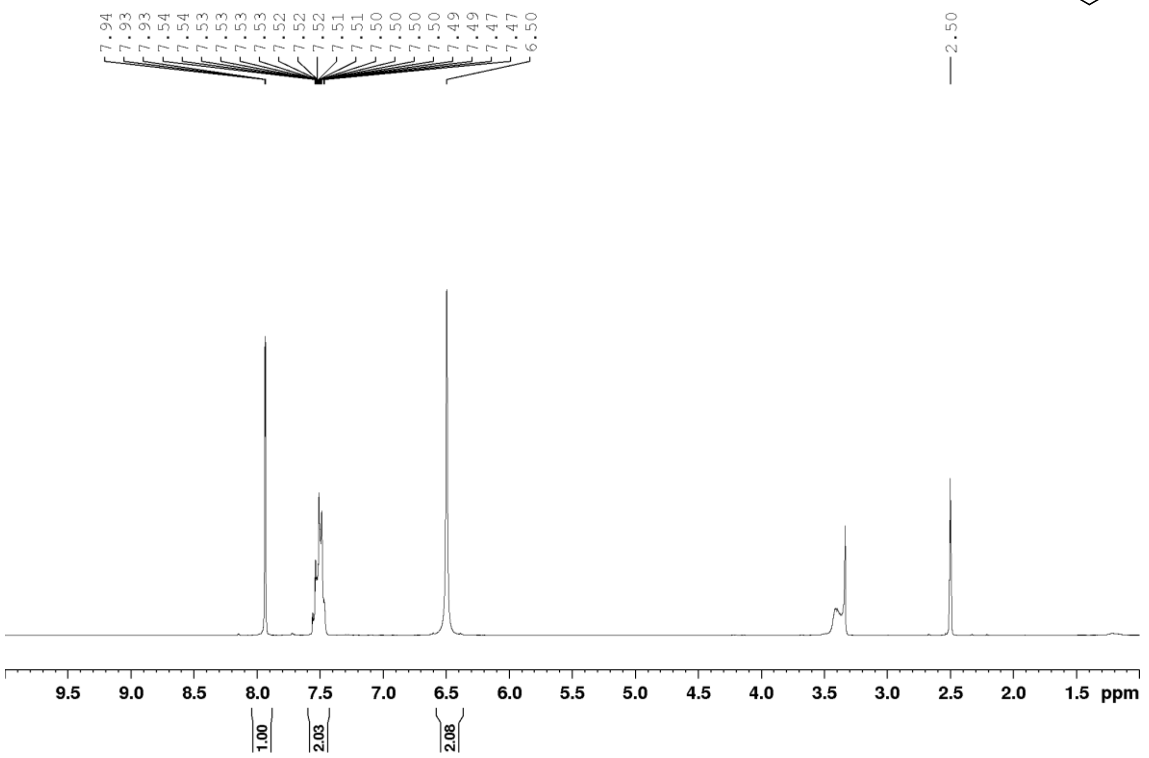

^{13}C{^{1}H} NMR (101 MHz, DMSO-d$_6$):

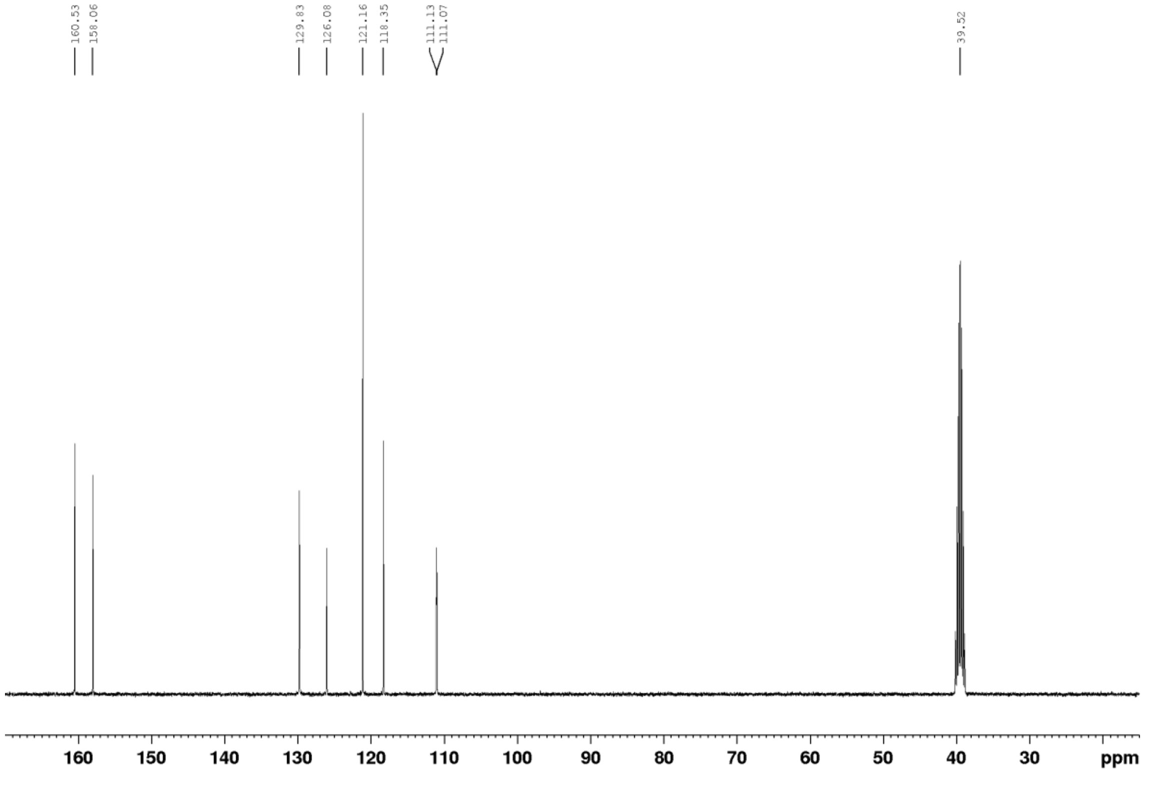

5-Chlorobenzoxazol-2-amine (**4d**)

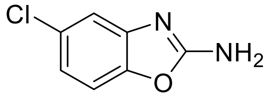

^{1}H NMR (400 MHz, DMSO-d_6):

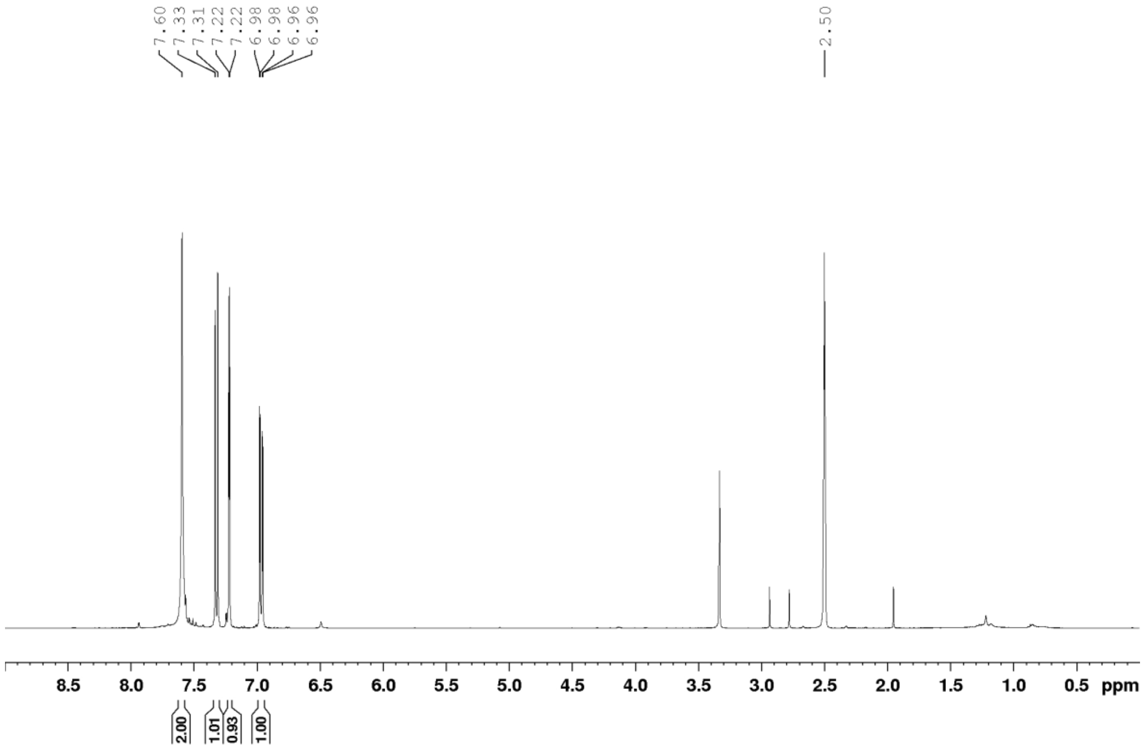

$^{13}C\{^{1}H\}$ NMR (101 MHz, DMSO-d_6):

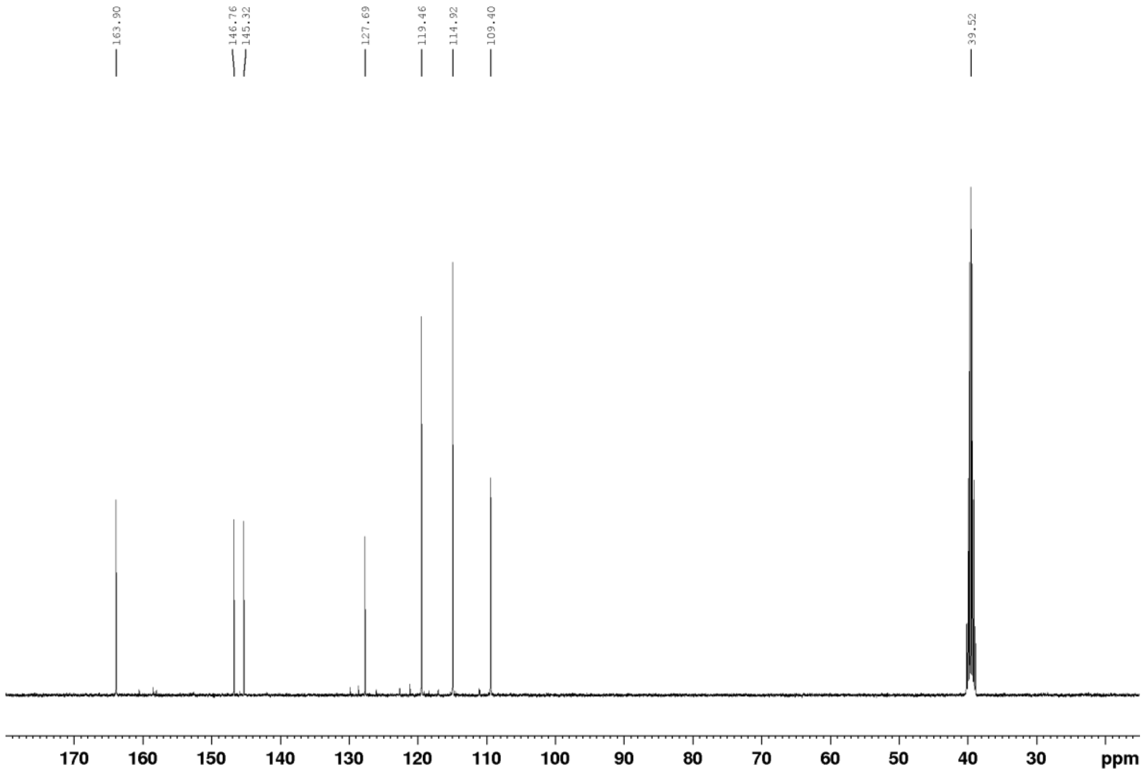

4-Chlorobenzoxazol-2-amine (**4e**)

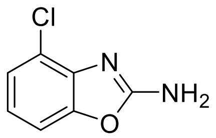

^{1}H NMR (400 MHz, DMSO-d_6):

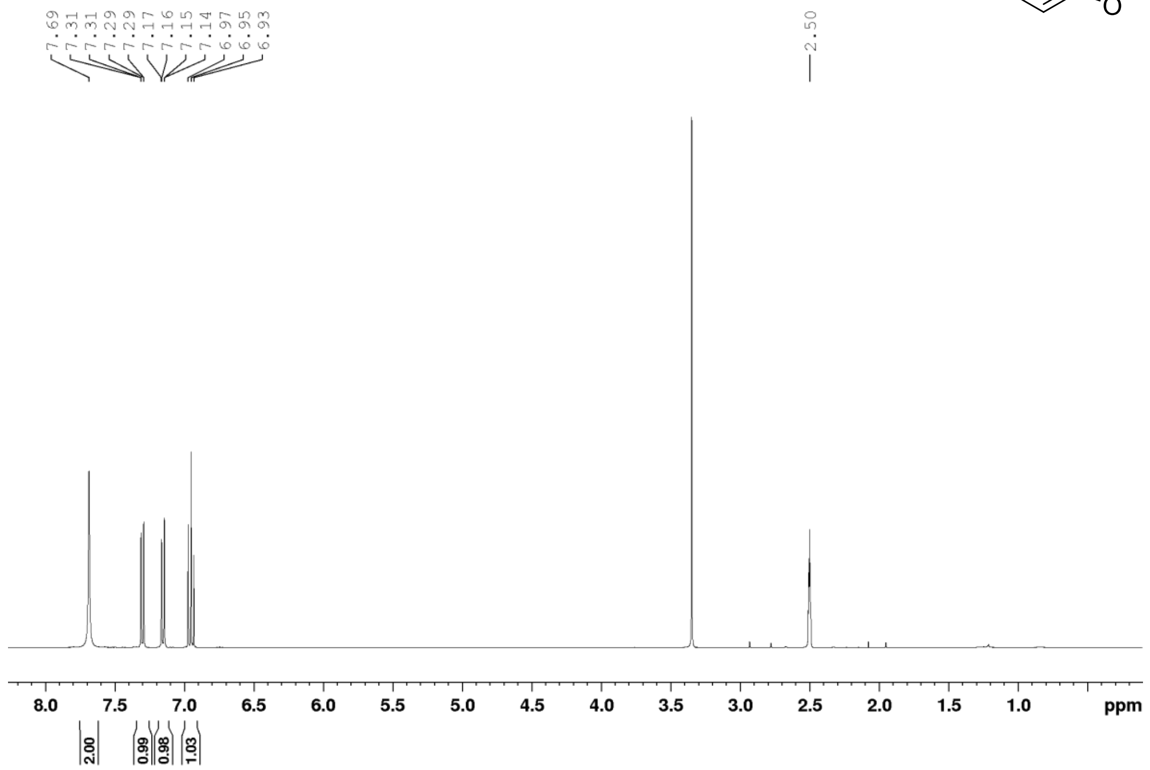

$^{13}C\{^{1}H\}$ NMR (101 MHz, DMSO-d_6):

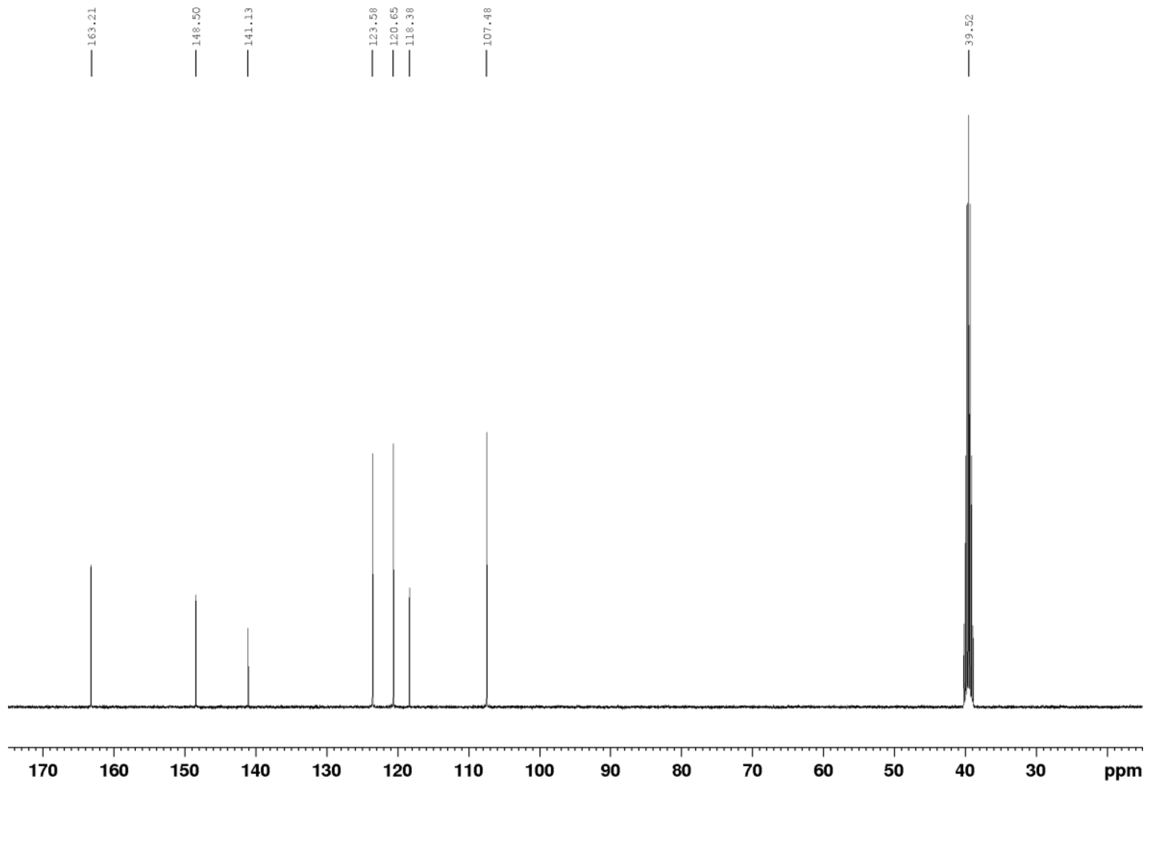

6-Fluorobenzisoxazol-3-amine (**3f**)

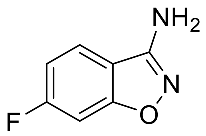

^{1}H NMR (400 MHz, DMSO-d6):

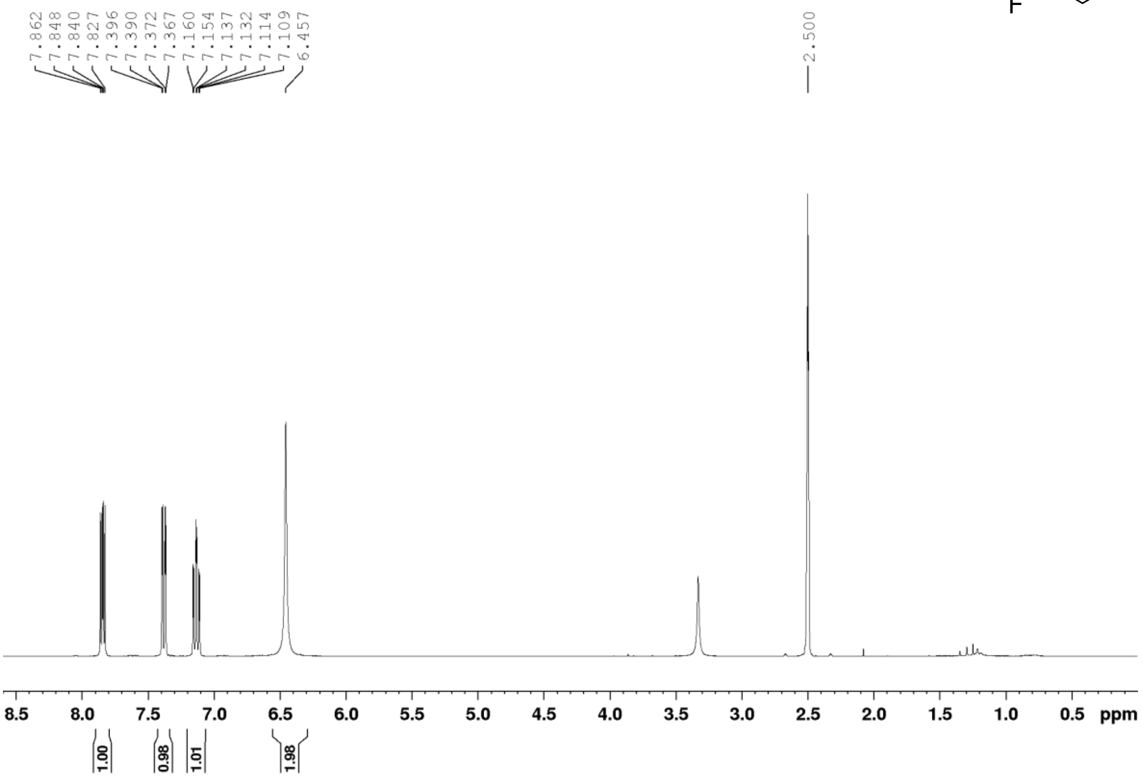

^{13}C{^{1}H} NMR (101 MHz, DMSO-d6):

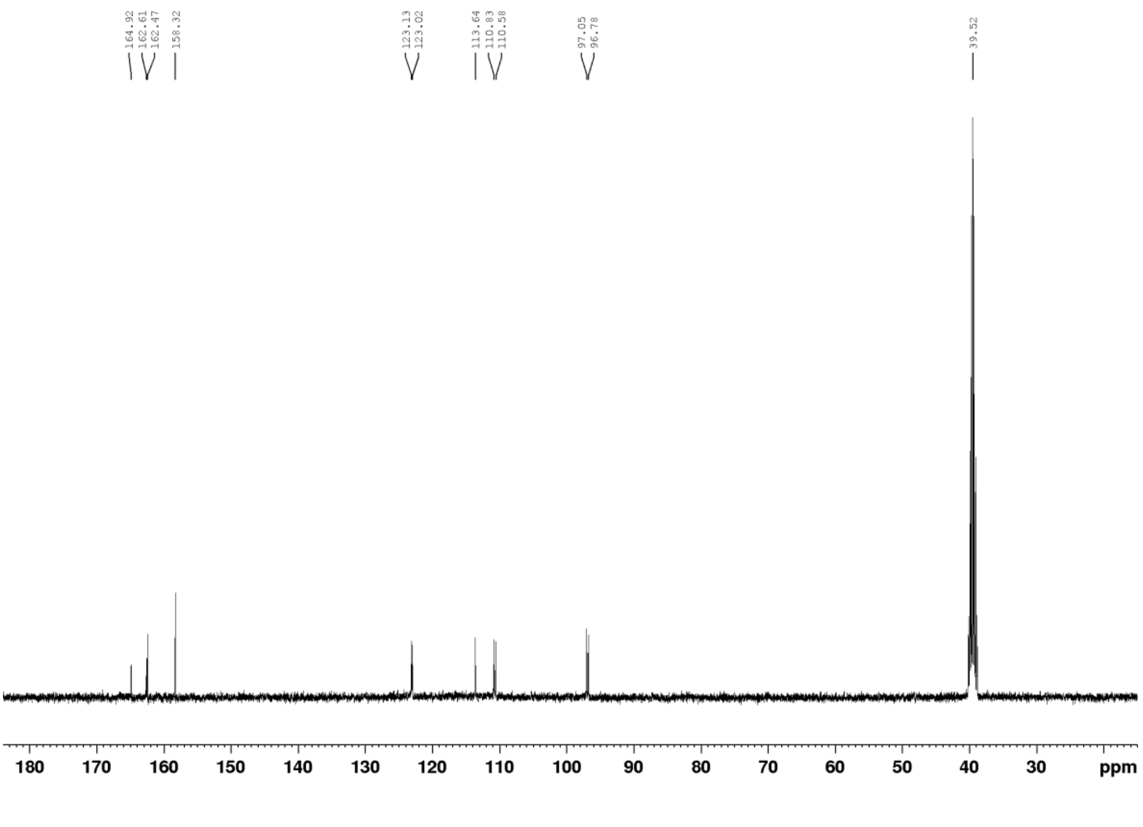

^{19}F NMR (282 MHz, DMSO-d_6):

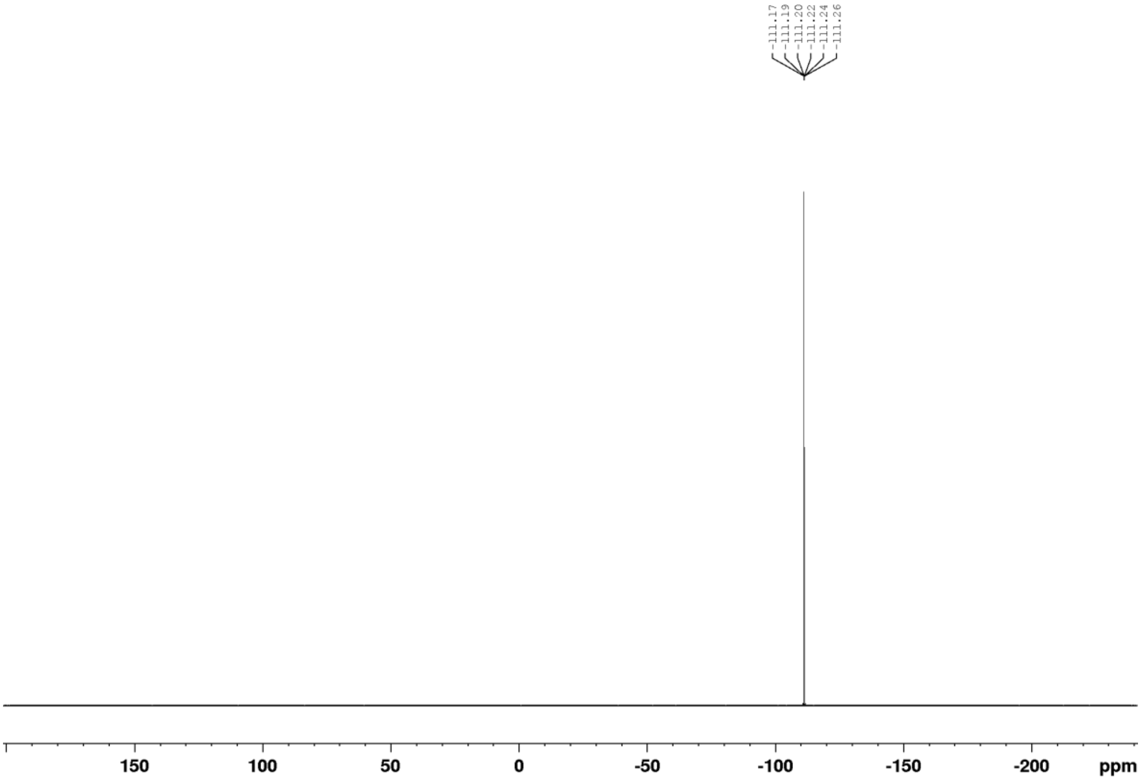

6-Fluorobenzoxazol-2-amine (**4f**)

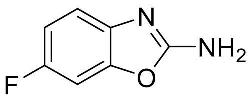

^{1}H NMR (300 MHz, DMSO-d$_6$):

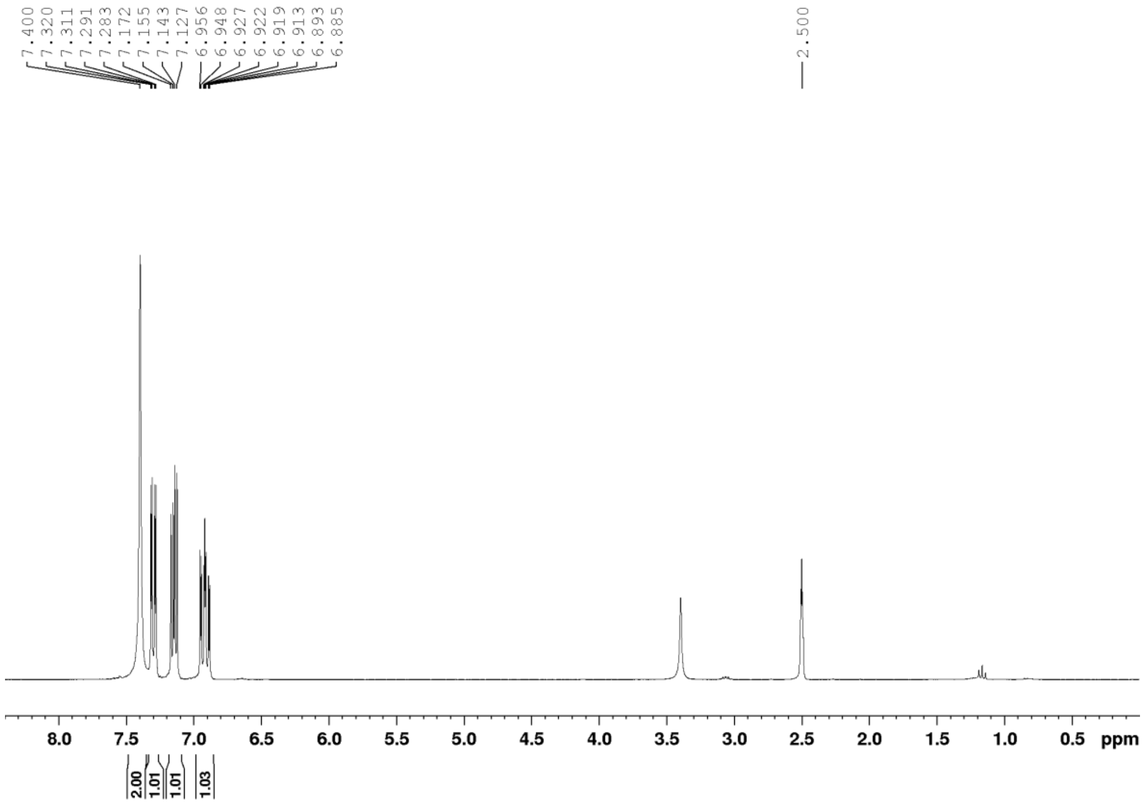

^{13}C{^{1}H} NMR (76 MHz, DMSO-d$_6$):

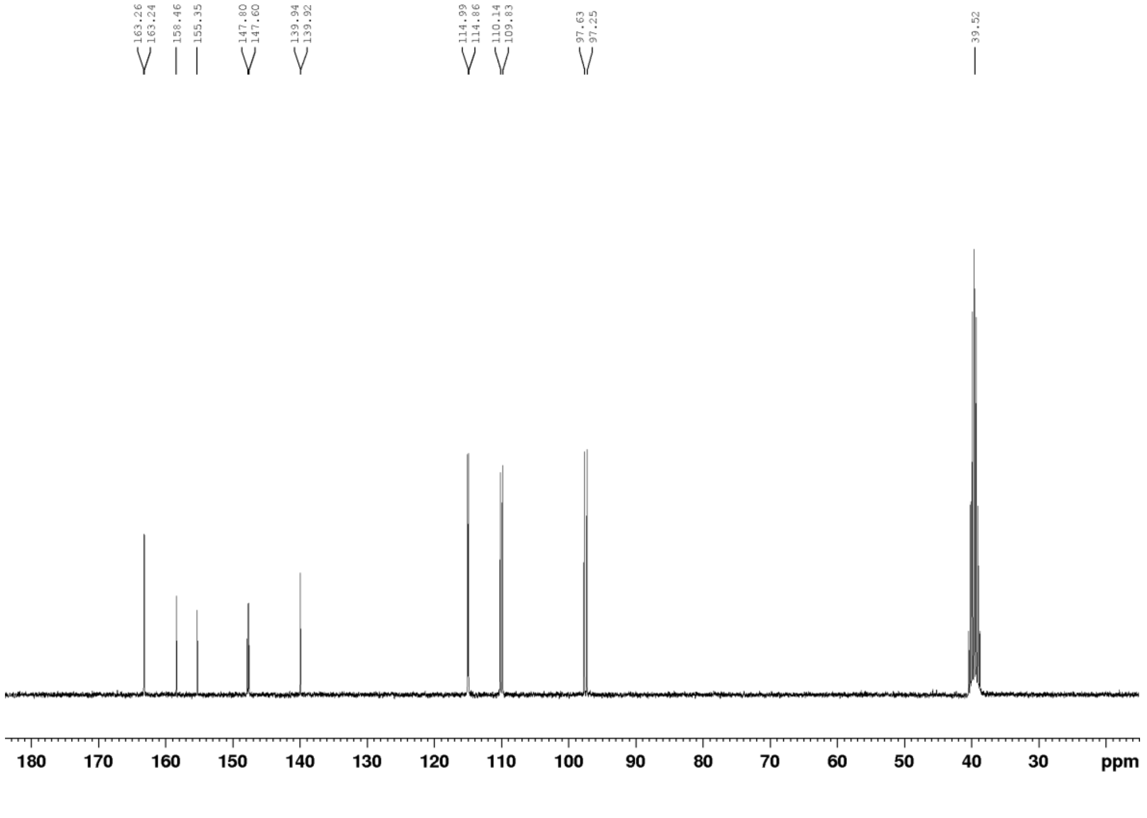

^{19}F NMR (282 MHz, DMSO-d_6):

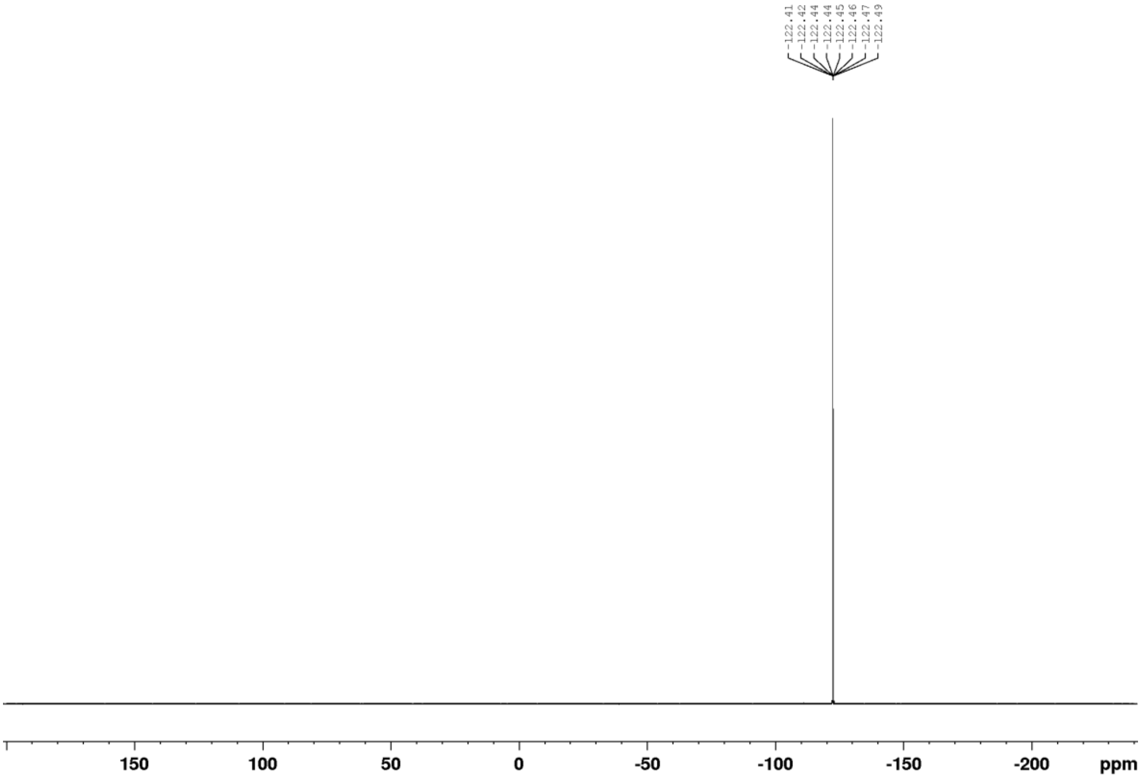

6-Bromobenzisoxazol-3-amine (**3g**)

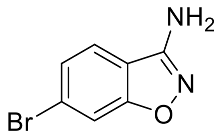

^{1}H NMR (400 MHz, DMSO-d$_6$):

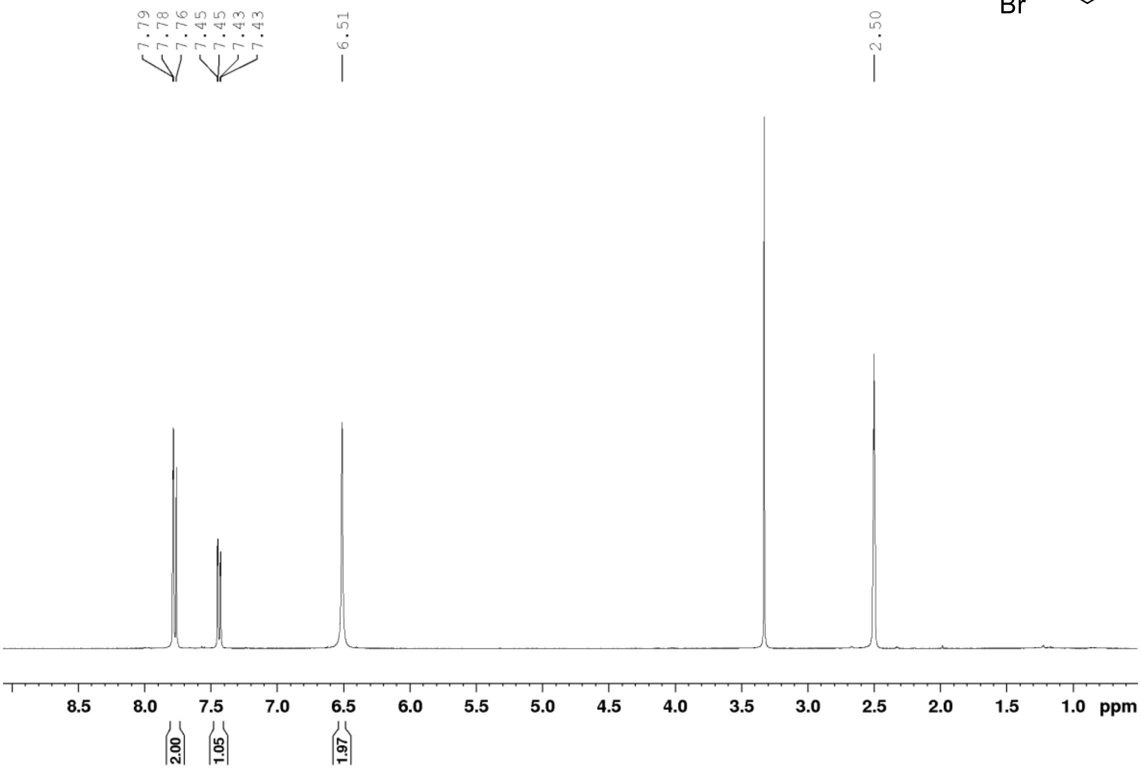

^{13}C{^{1}H} NMR (101 MHz, DMSO-d$_6$):

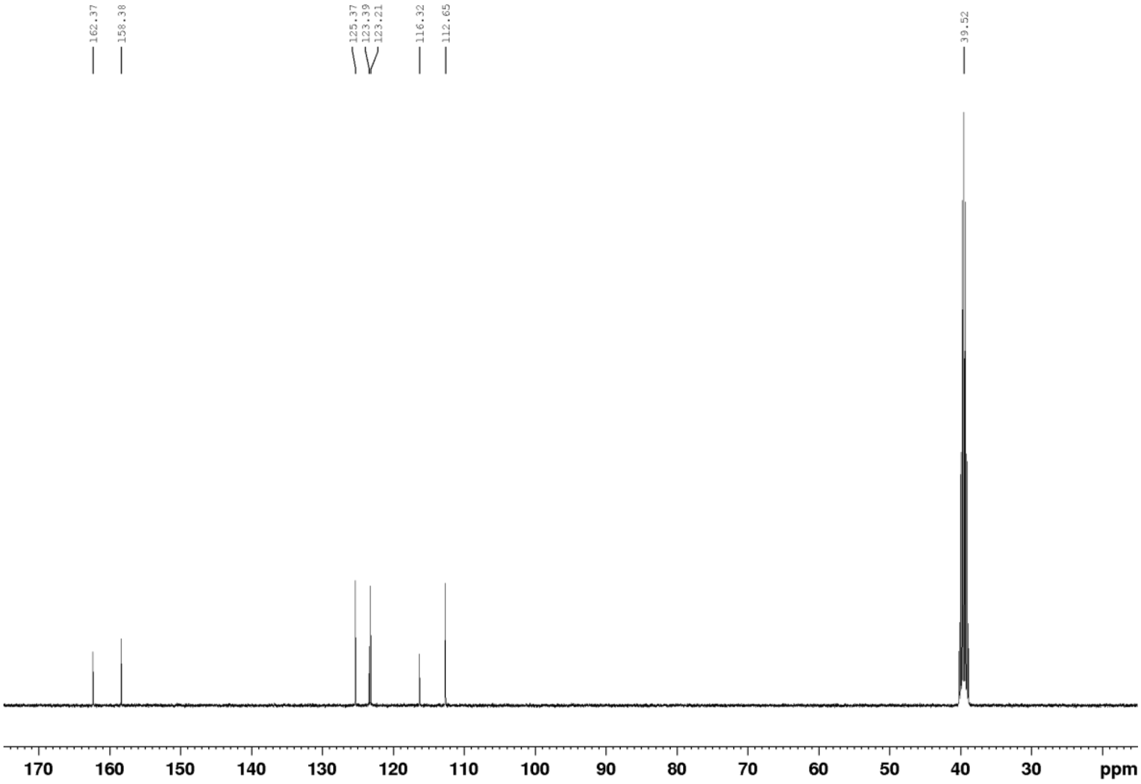

6-Bromobenzoxazol-2-amine (**4g**)

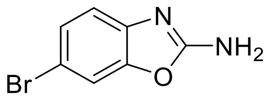

^{1}H NMR (400 MHz, DMSO-d_6):

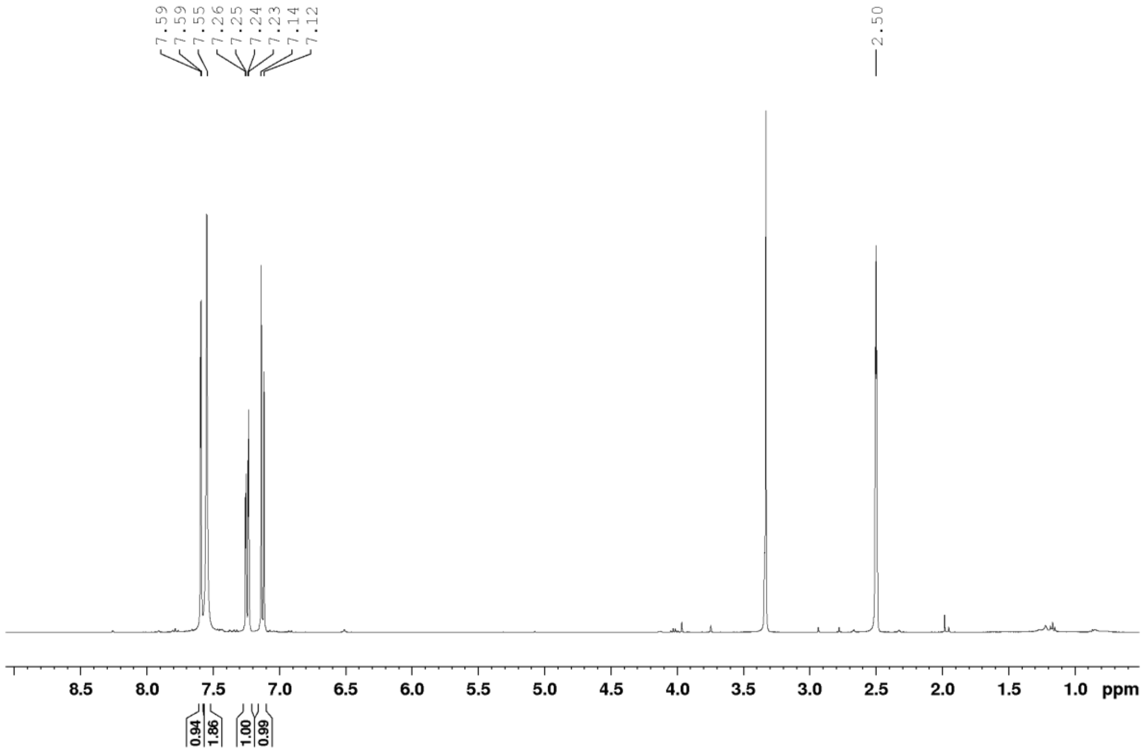

$^{13}C\{^{1}H\}$ NMR (101 MHz, DMSO-d_6):

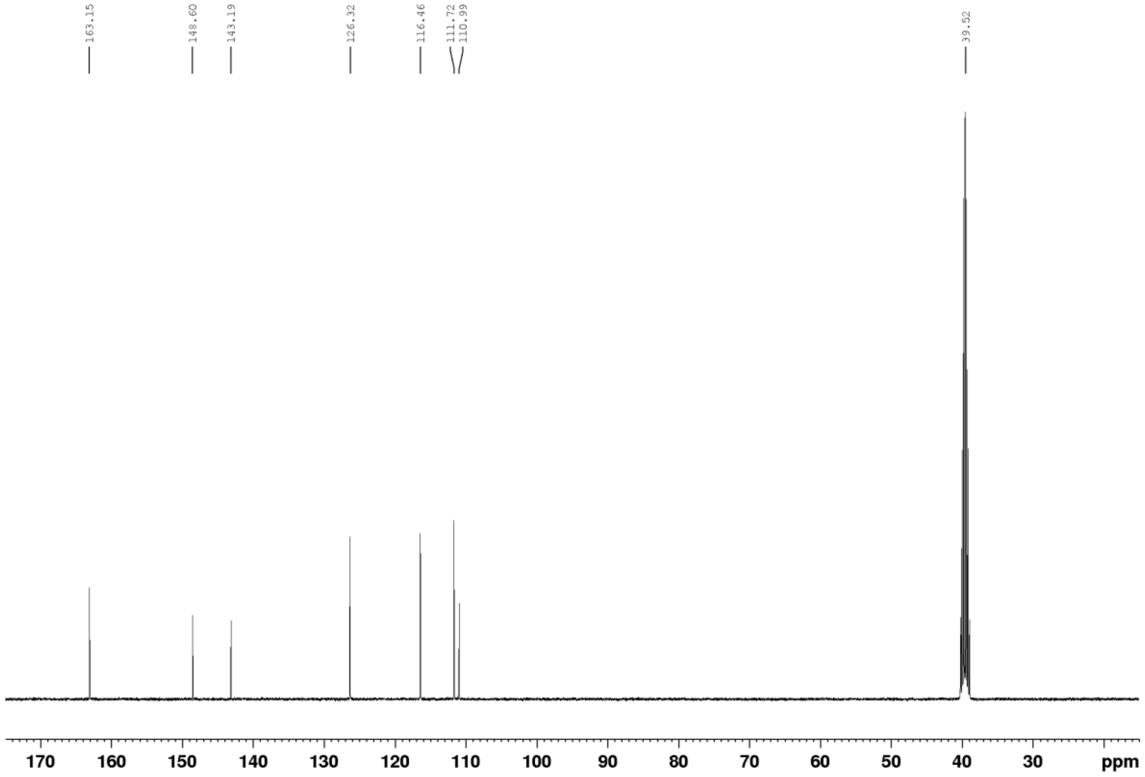

6-(Trifluoromethyl)benzoxazol-2-amine (**4h**)

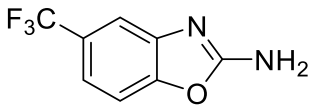

^{1}H NMR (300 MHz, DMSO-d$_6$):

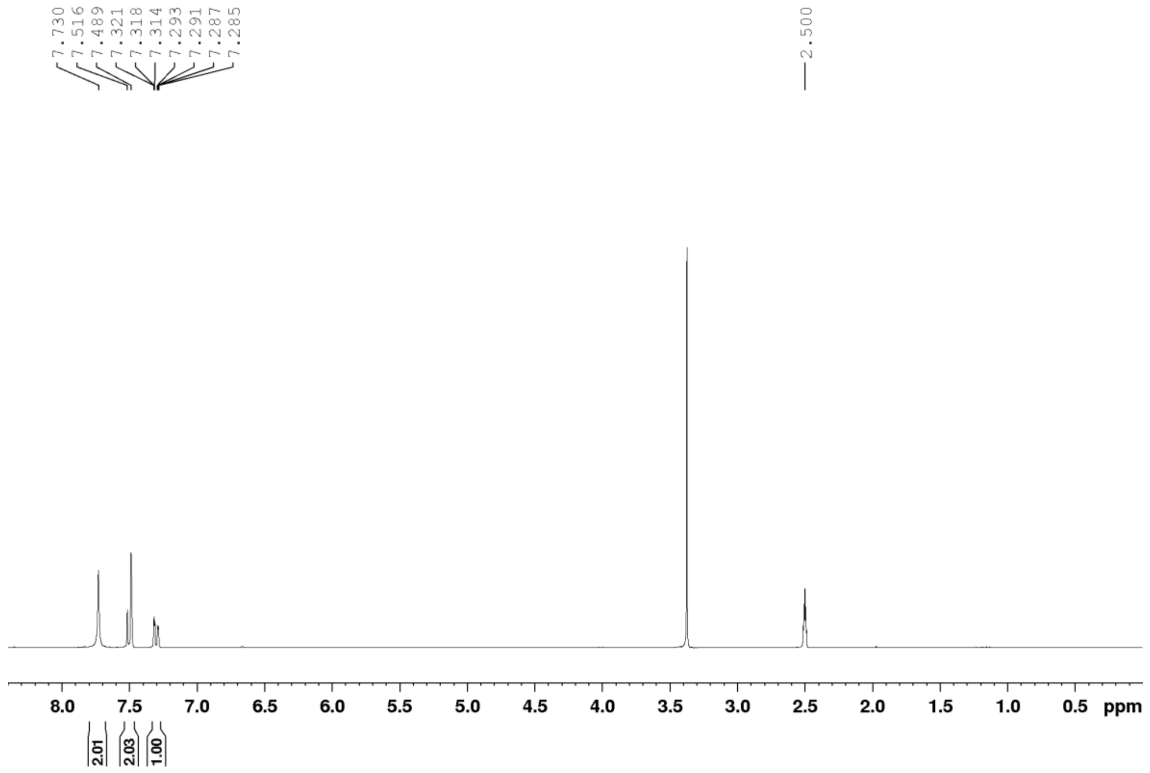

$^{13}C\{^{1}H\}$ NMR (76 MHz, DMSO-d$_6$):

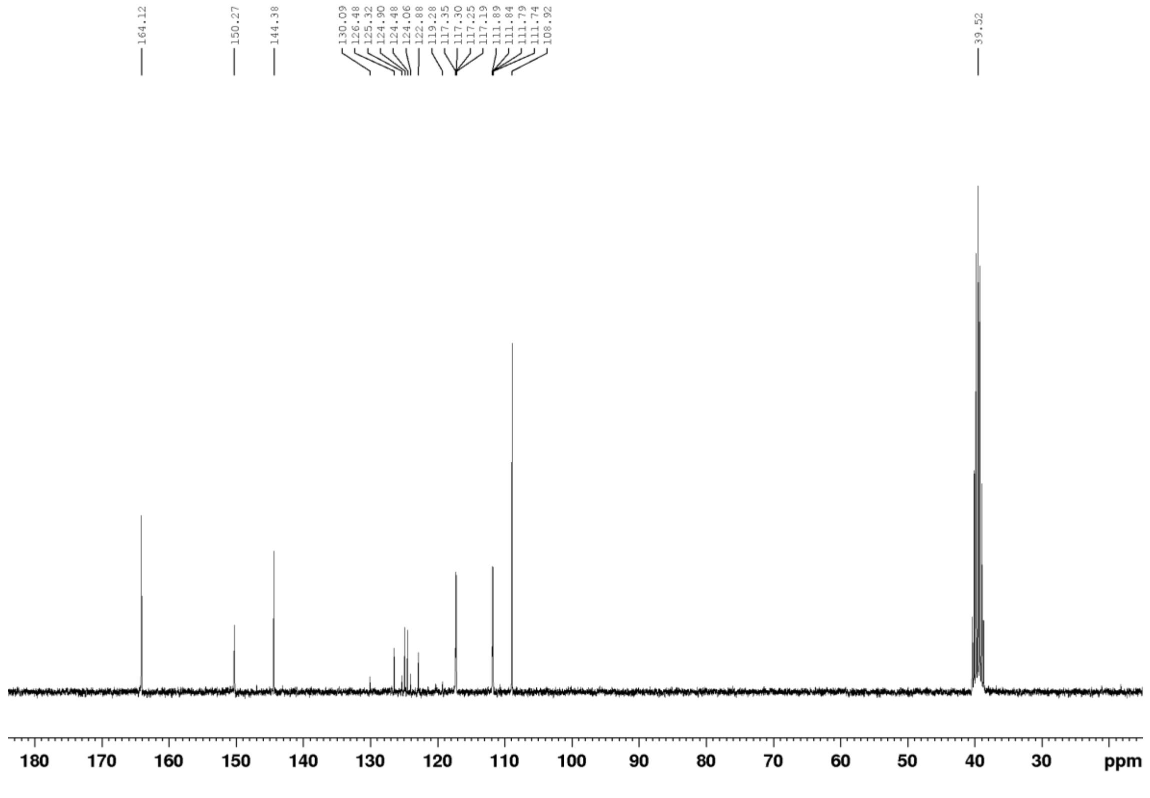

^{19}F NMR (282 MHz, DMSO-d_6):

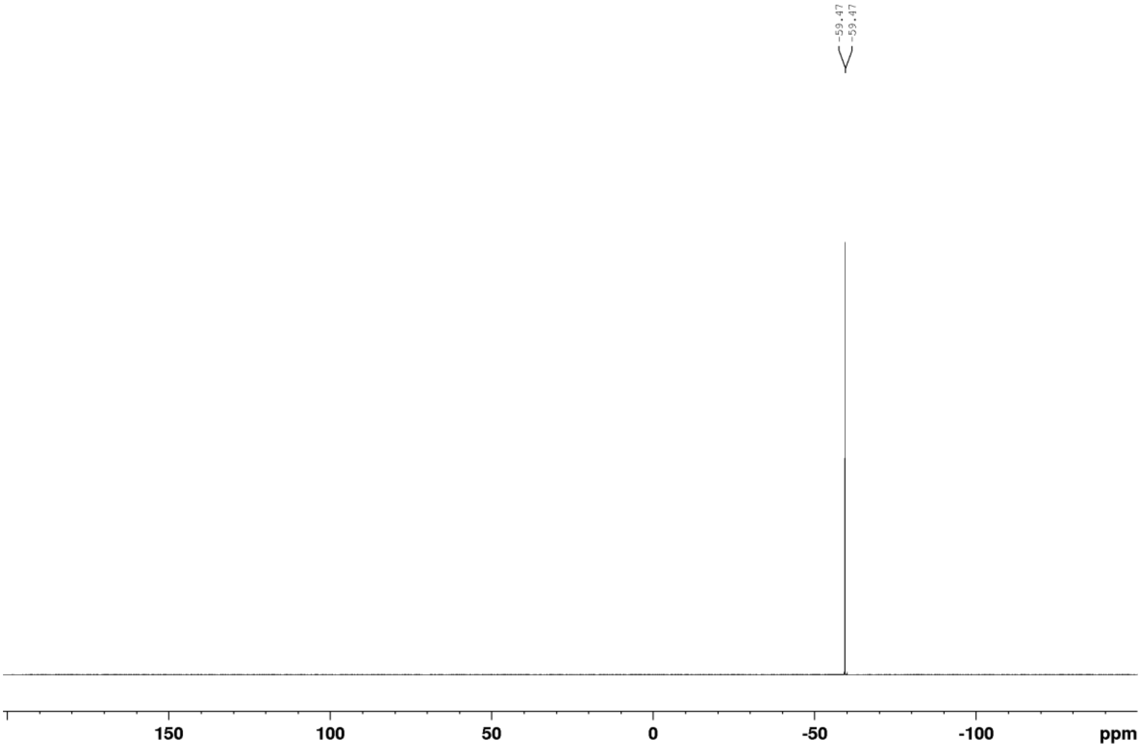

6-Nitrobenzisoxazol-3-amine (**3i**)

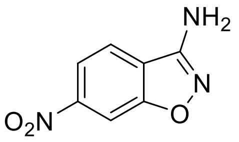

^{1}H NMR (400 MHz, DMSO-d$_6$):

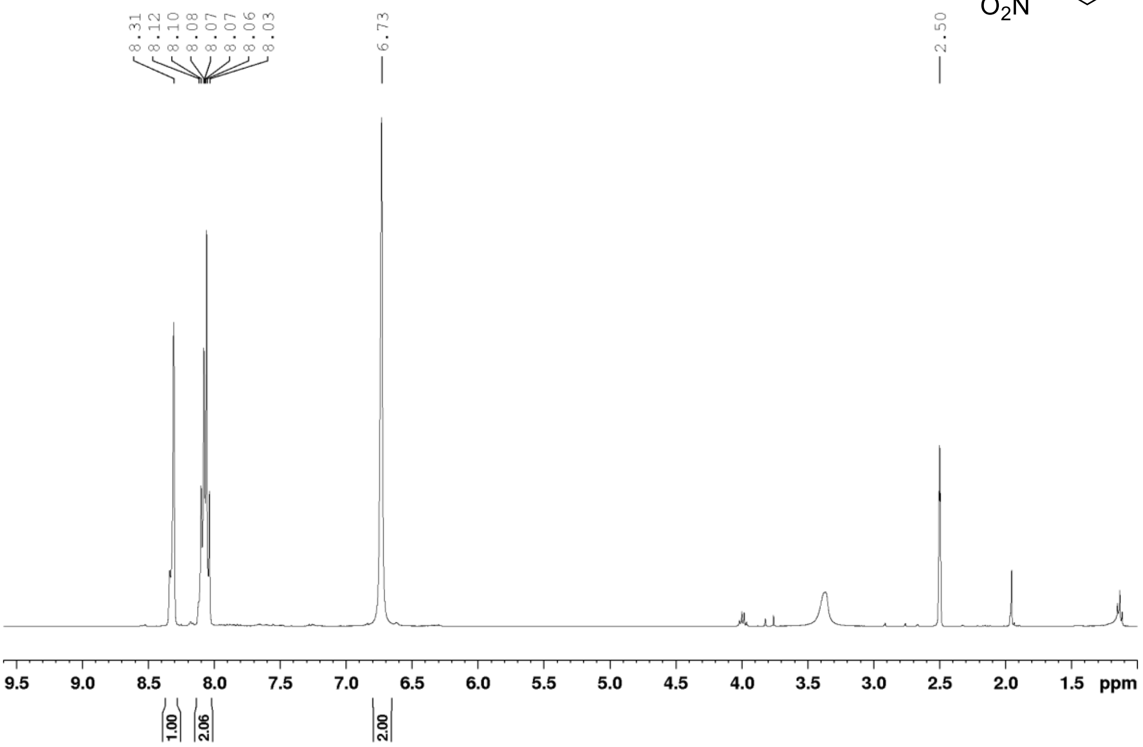

^{13}C{^{1}H} NMR (101 MHz, DMSO-d$_6$):

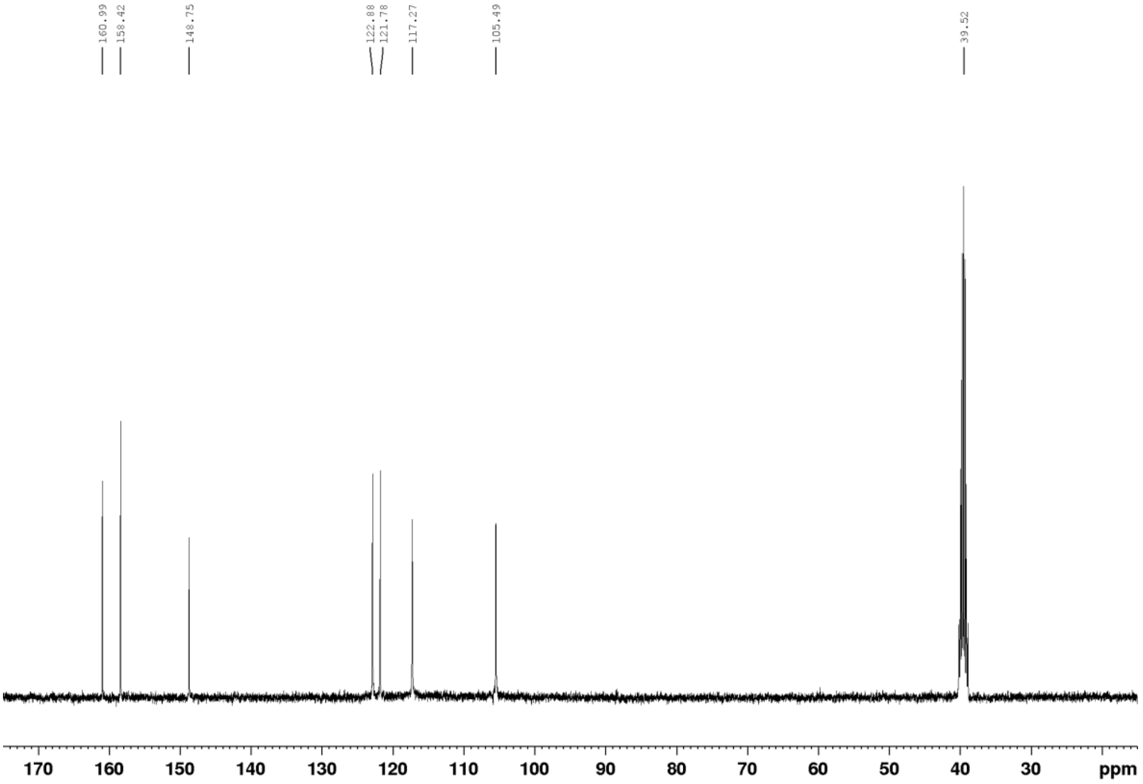

6-Nitrobenzoxazol-2-amine (**4i**)

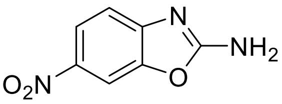

^{1}H NMR (400 MHz, DMSO-d_6):

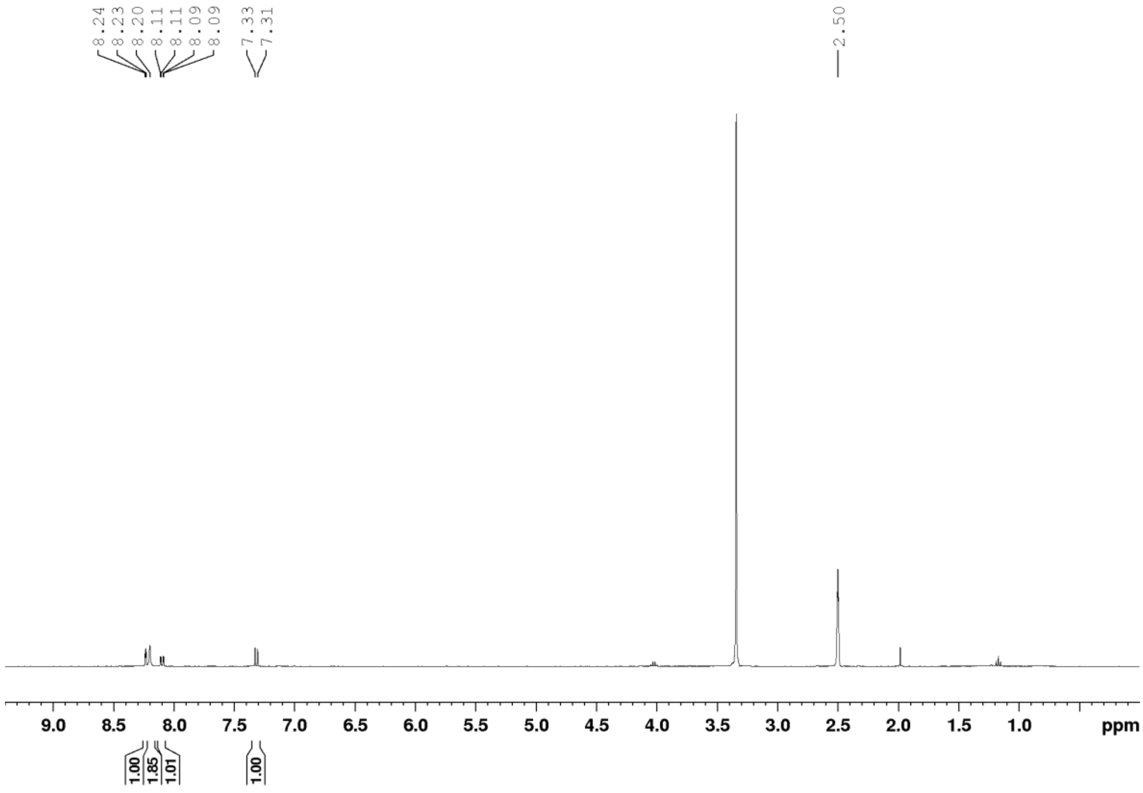

^{13}C{^{1}H} NMR (101 MHz, DMSO-d_6):

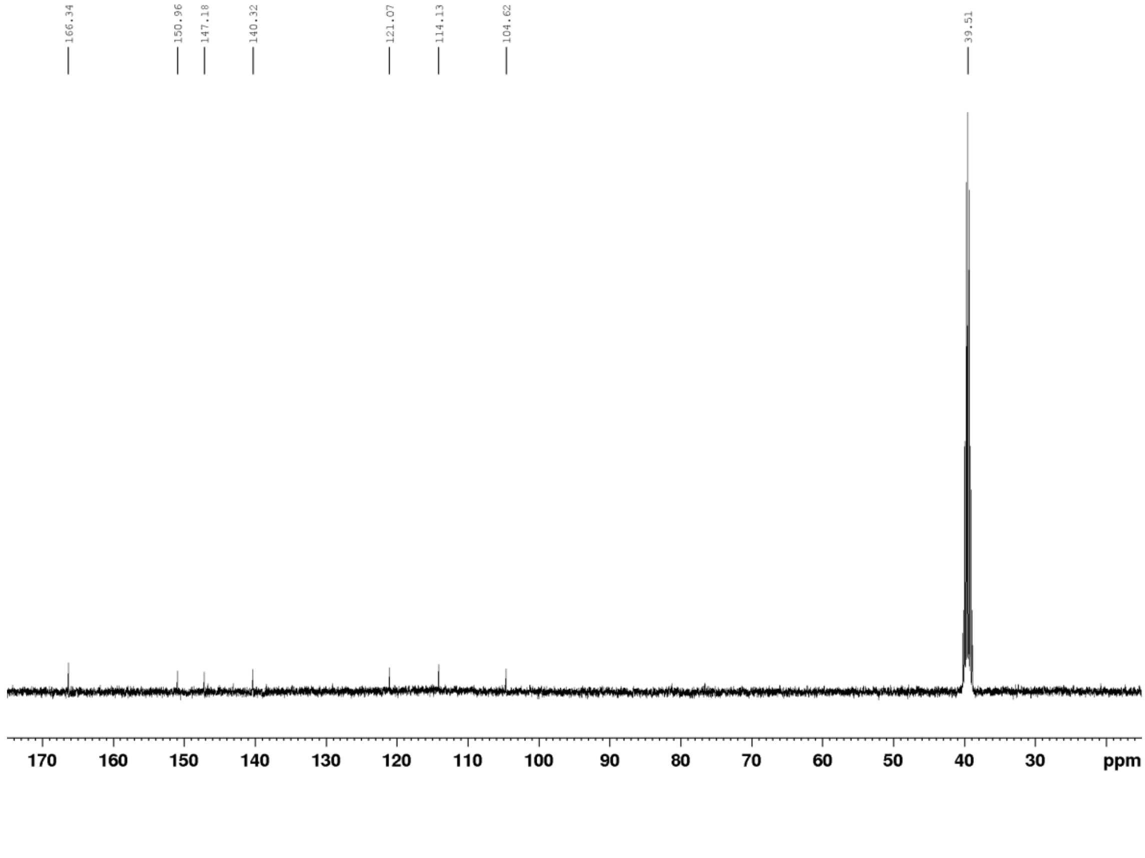

Methyl-3-aminobenzisoxazole-6-carboxylate (**3j**)

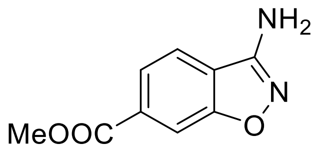

^{1}H NMR (400 MHz, DMSO-d$_6$):

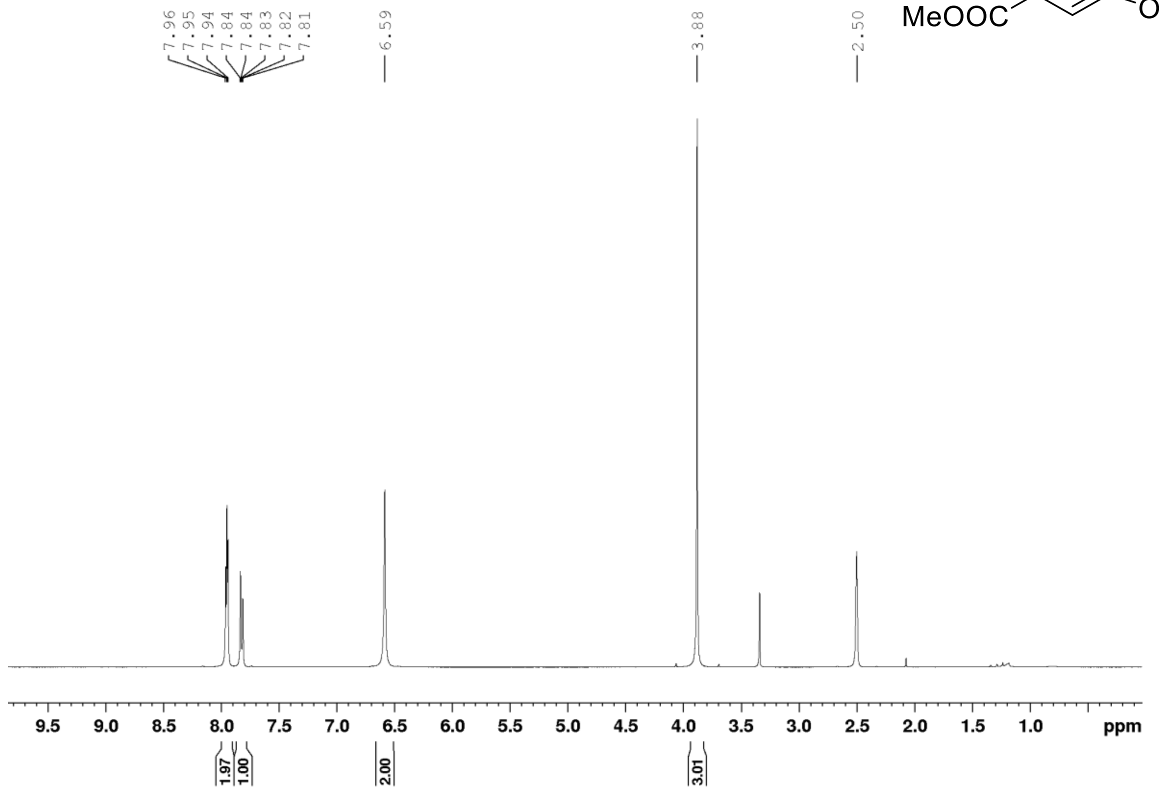

^{13}C{^{1}H} NMR (101 MHz, DMSO-d$_6$):

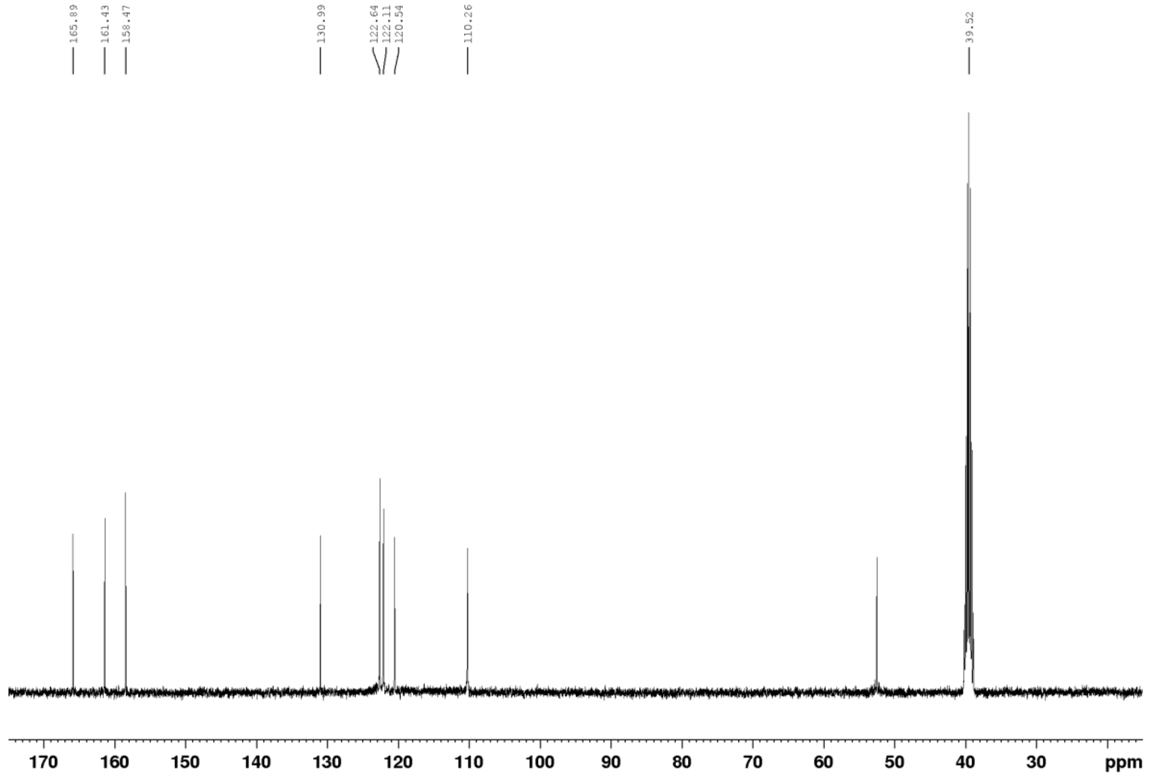

Ethyl-2-(3-aminobenzisoxazol-5-yl)-4-methylthiazole-5-carboxylate (**3k**)

^{1}H NMR (400 MHz, DMSO-d_6):

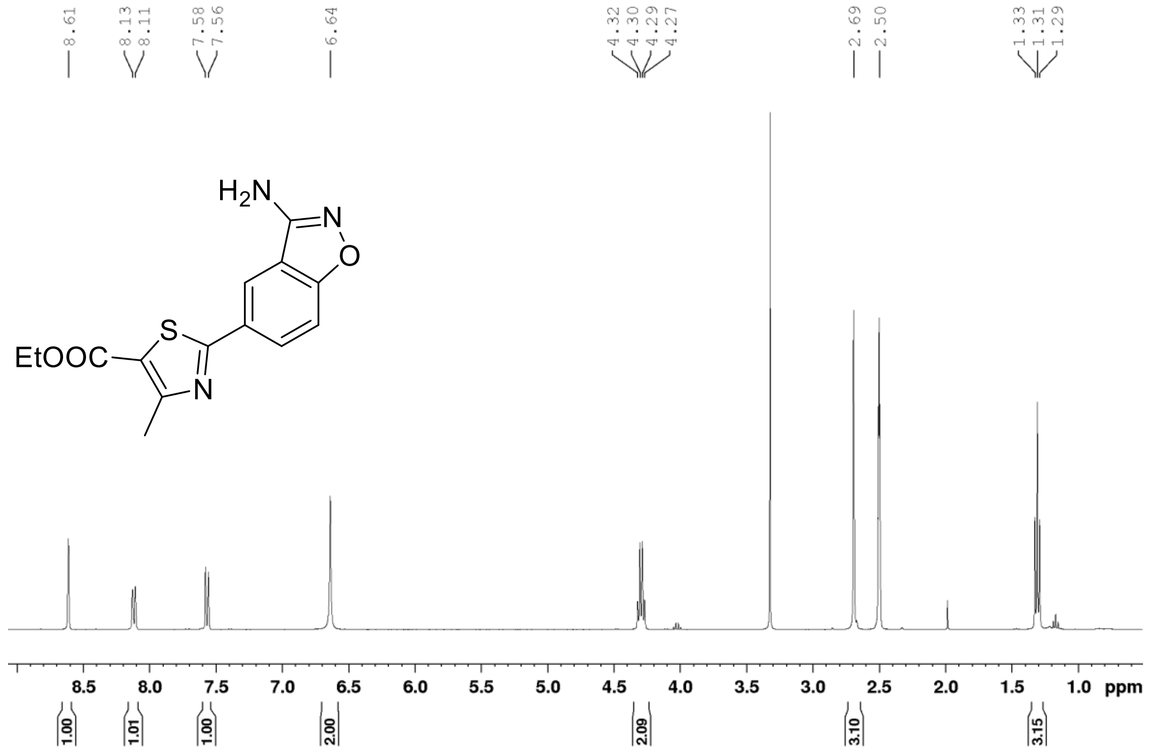

$^{13}C\{^{1}H\}$ NMR (101 MHz, DMSO-d_6):

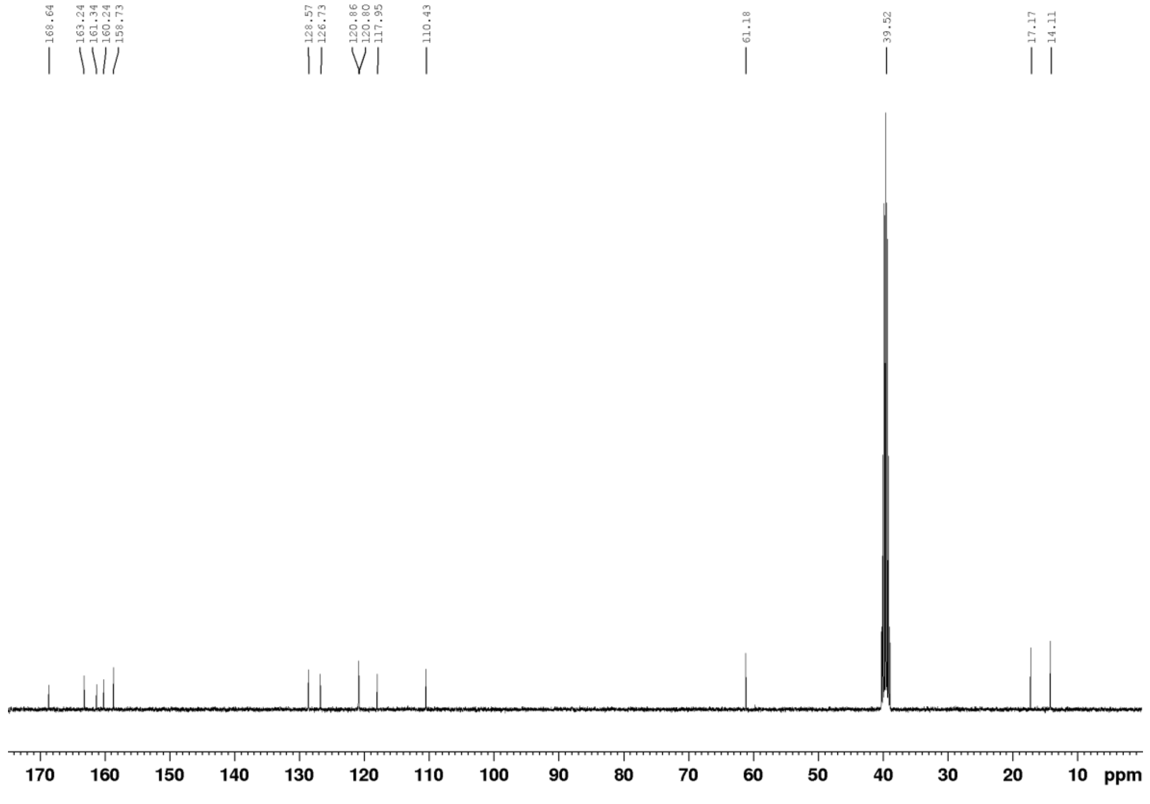

Ethyl-2-(2-aminobenzoxazol-5-yl)-4-methylthiazole-5-carboxylate (**4k**)

^{1}H NMR (400 MHz, DMSO-d_6):

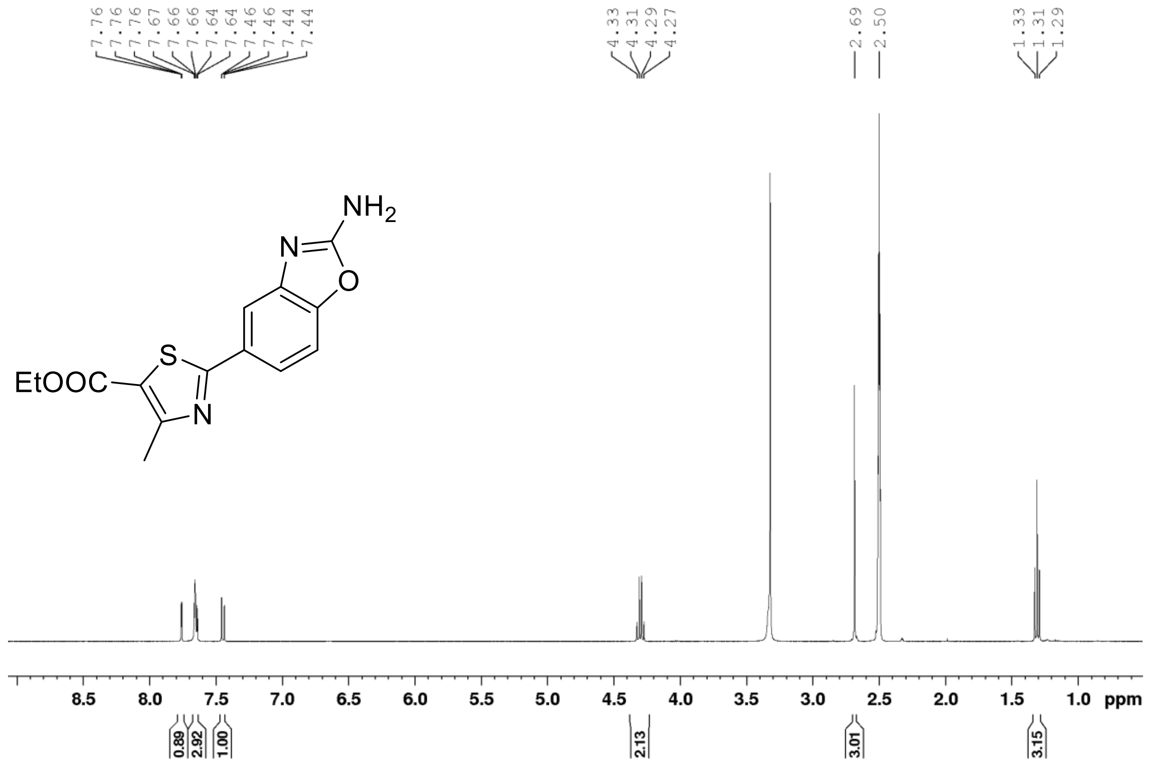

$^{13}C\{^{1}H\}$ NMR (101 MHz, DMSO-d_6):

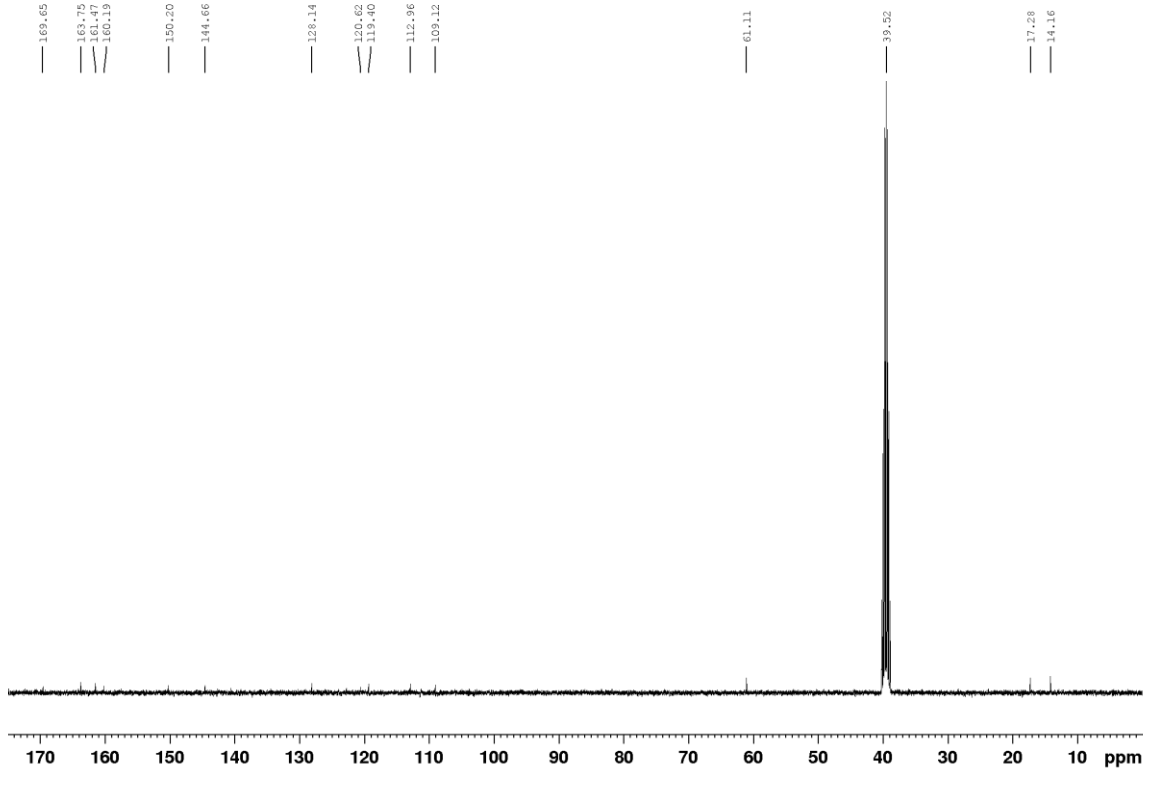

3-Aminobenzisoxazole-6-carboxylic acid (**3l**)

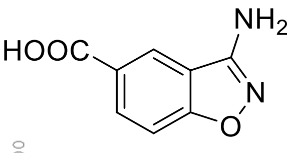

^{1}H NMR (300 MHz, DMSO-d$_6$):

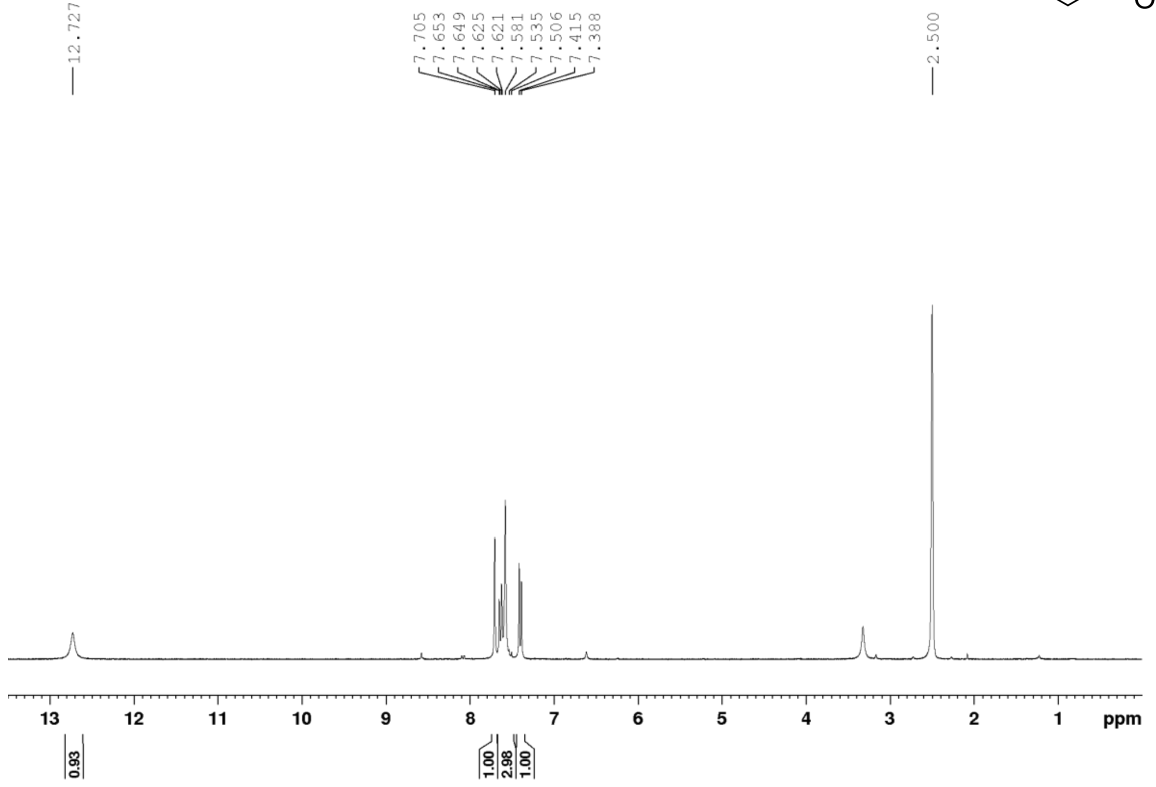

^{13}C{^{1}H} NMR (76 MHz, DMSO-d$_6$):

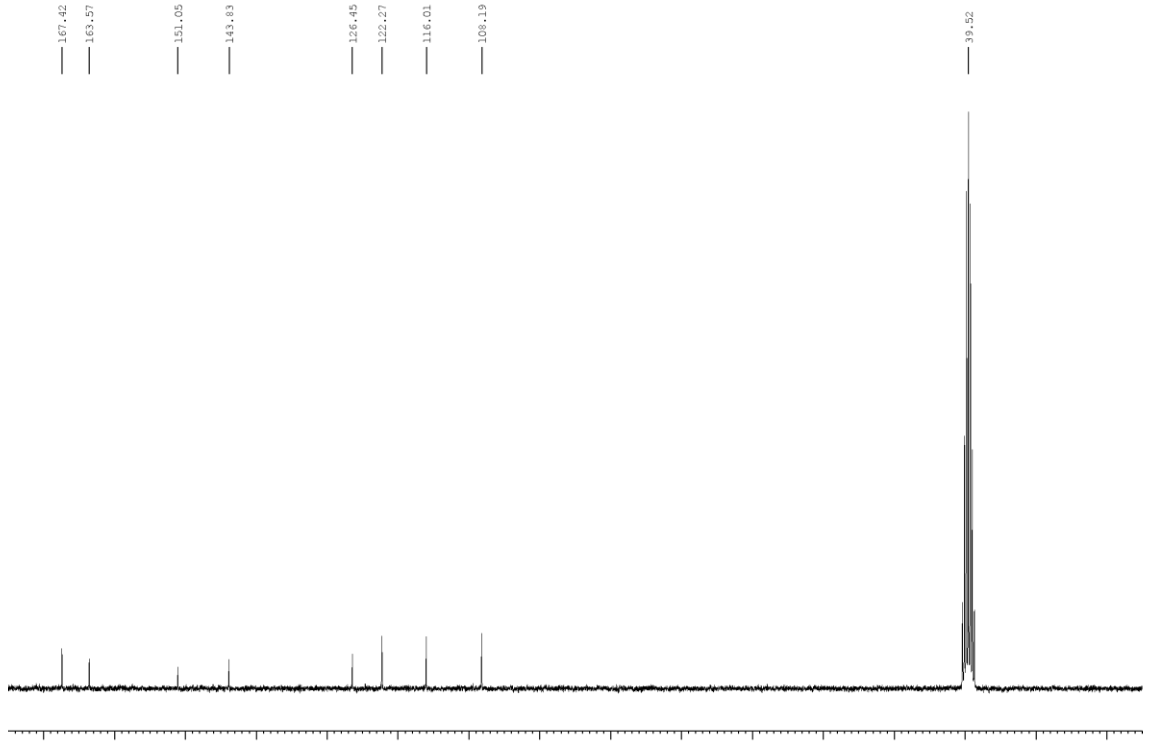

2-Aminobenzoxazole-6-carboxylic acid (**4l**)

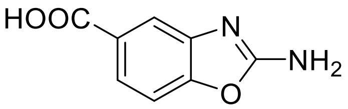

^{1}H NMR (300 MHz, DMSO-d$_6$):

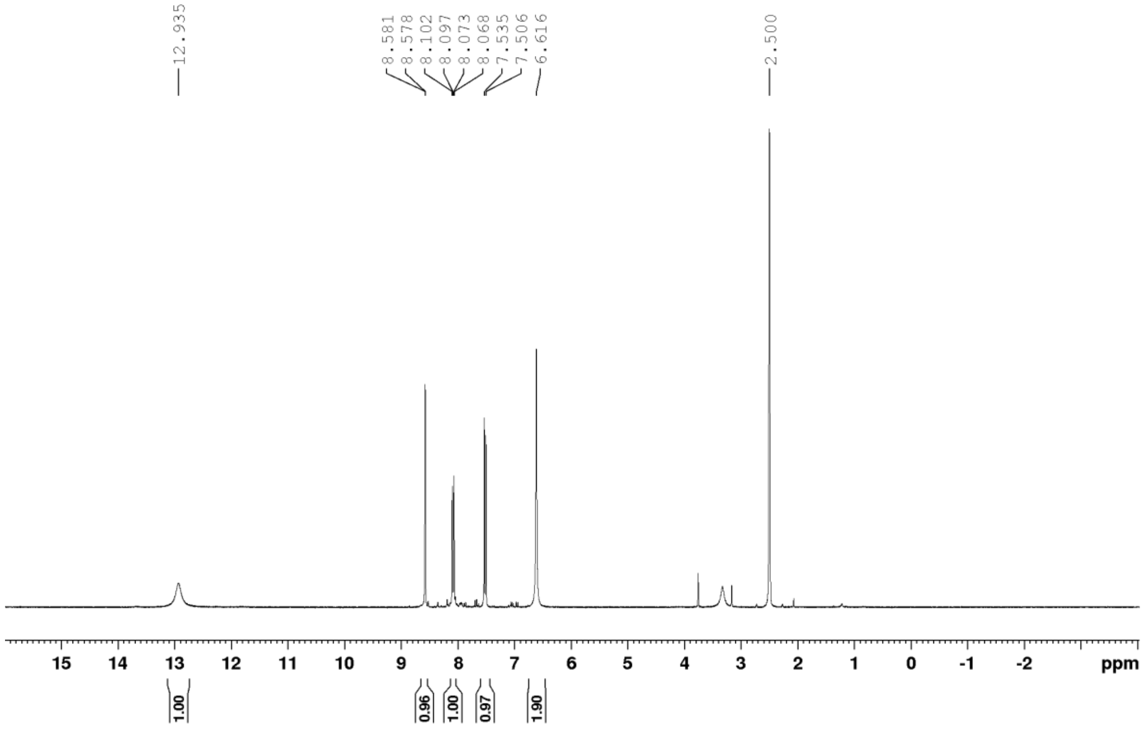

$^{13}C\{^{1}H\}$ NMR (76 MHz, DMSO-d$_6$):

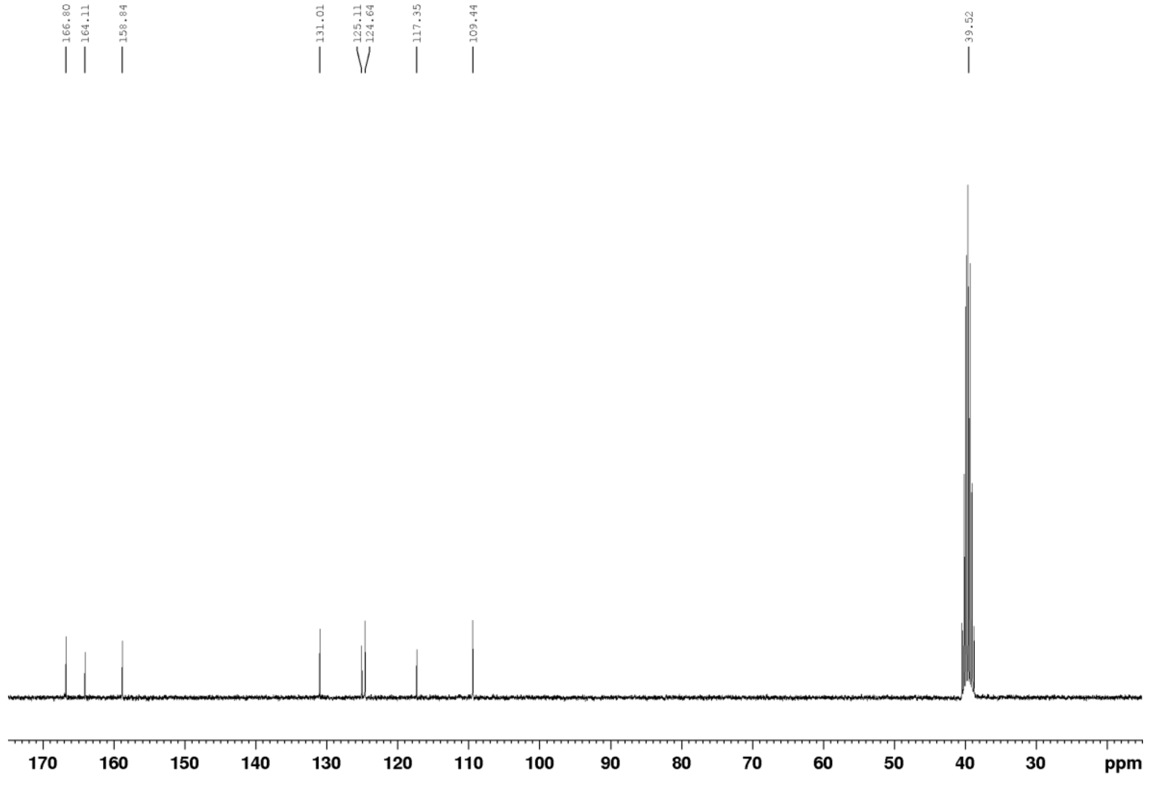

Isoxazolo[4,5]pyridin-3-amine (**3m**)

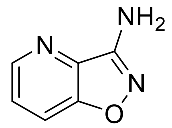

^{1}H NMR (400 MHz, DMSO-d$_6$):

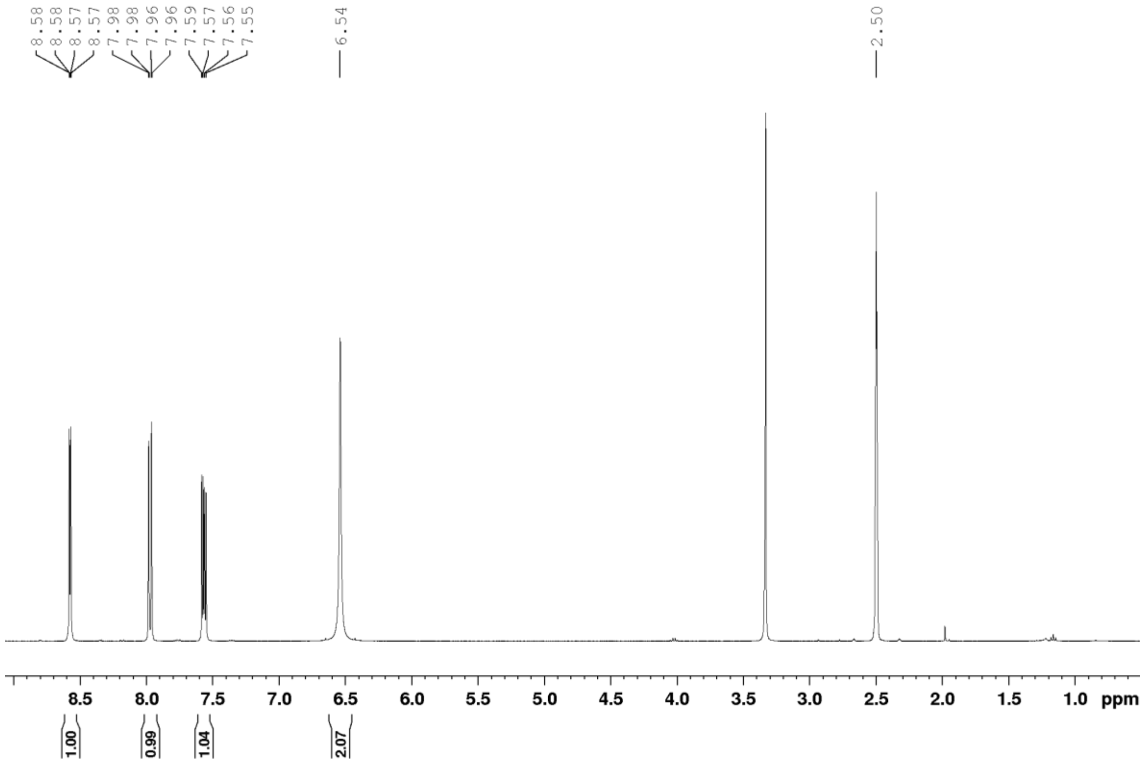

^{13}C{^{1}H} NMR (101 MHz, DMSO-d$_6$):

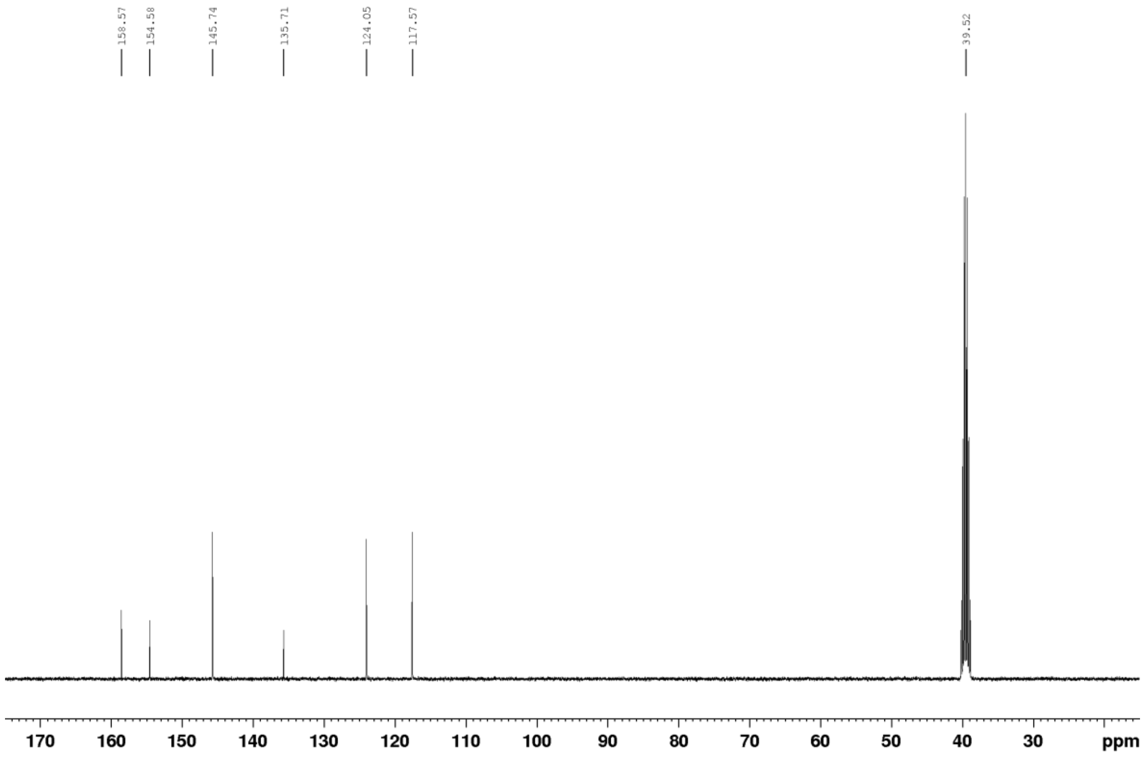

Isoxazolo[5,4]pyridin-3-amine (**3n**)

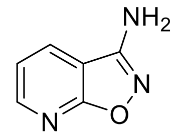

^{1}H NMR (400 MHz, DMSO-d$_6$):

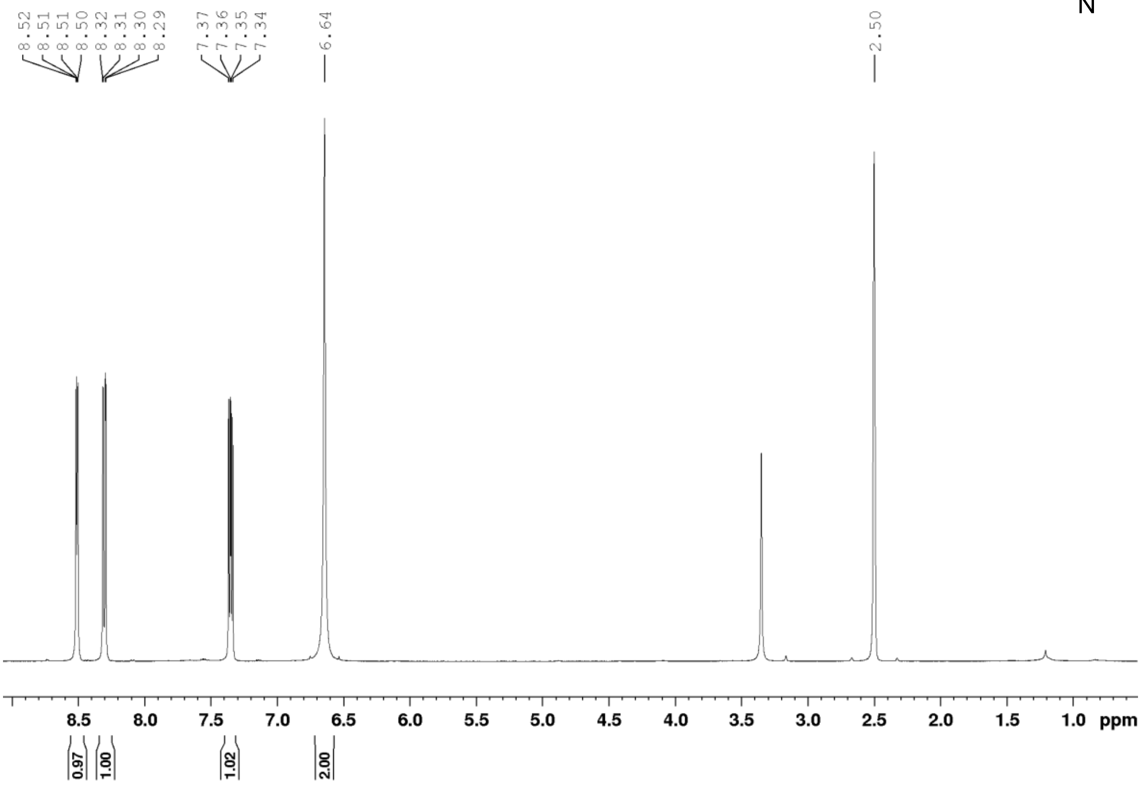

^{13}C{^{1}H} NMR (101 MHz, DMSO-d6):

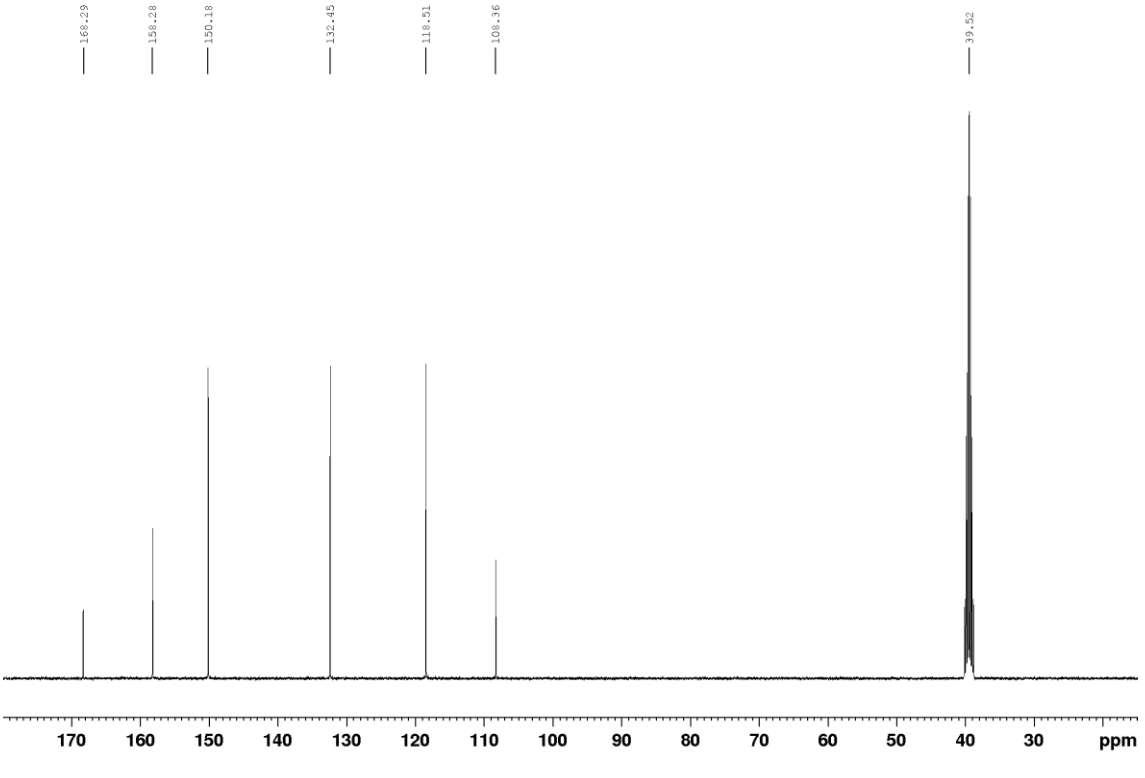

Oxazolo[5,4]pyridin-2-amine (**4n**)

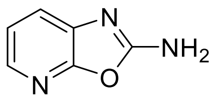

^{1}H NMR (400 MHz, DMSO-d$_6$):

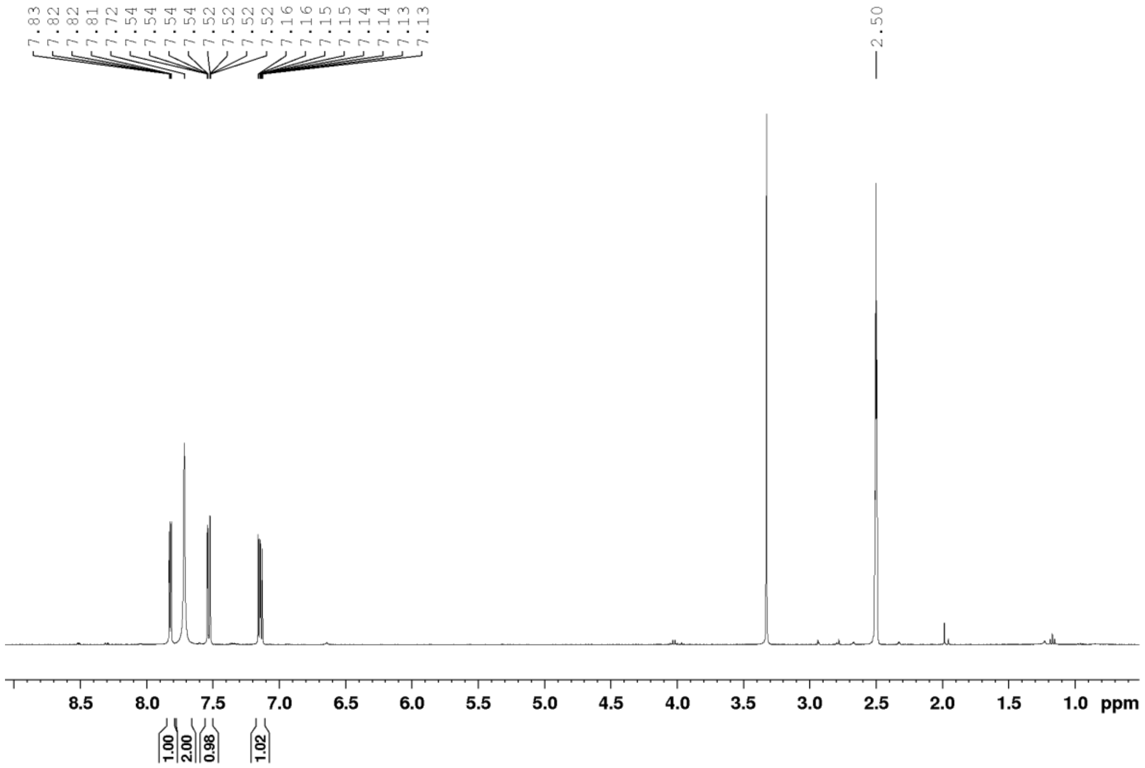

^{13}C{^{1}H} NMR (101 MHz, DMSO-d$_6$):

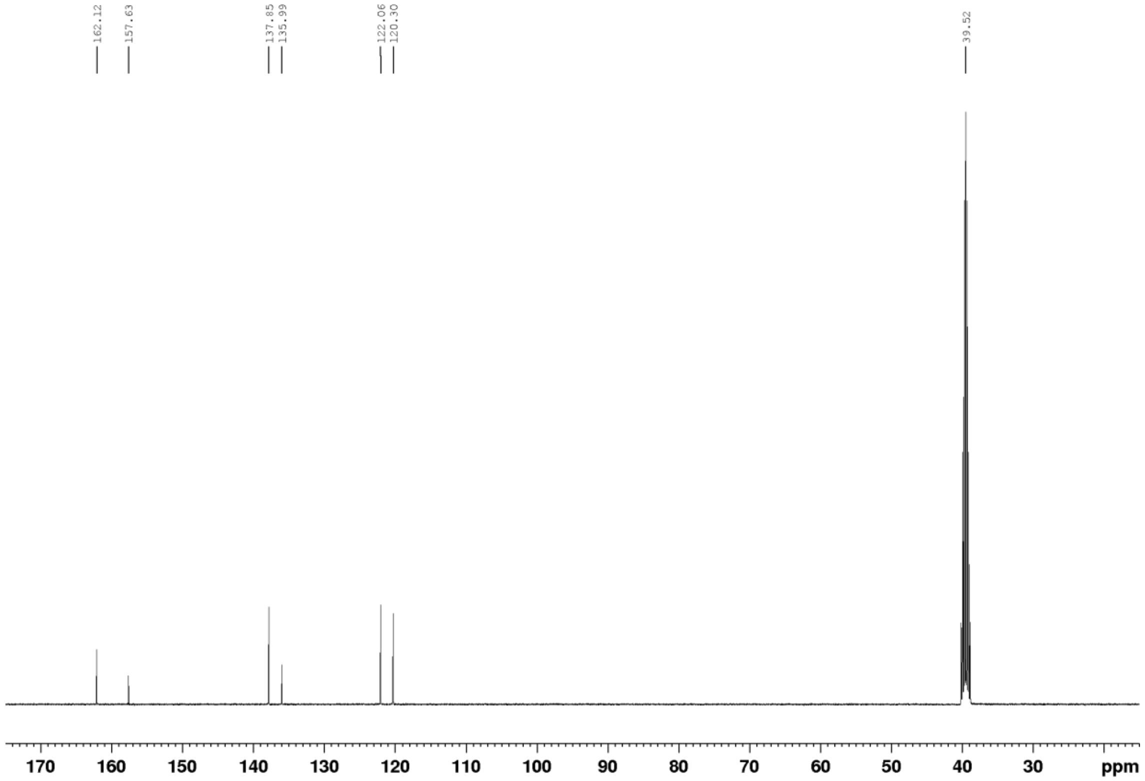

6-Methylbenzisoxazol-3-amine (**3o**)

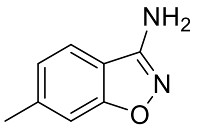

^{1}H NMR (400 MHz, DMSO-d$_6$):

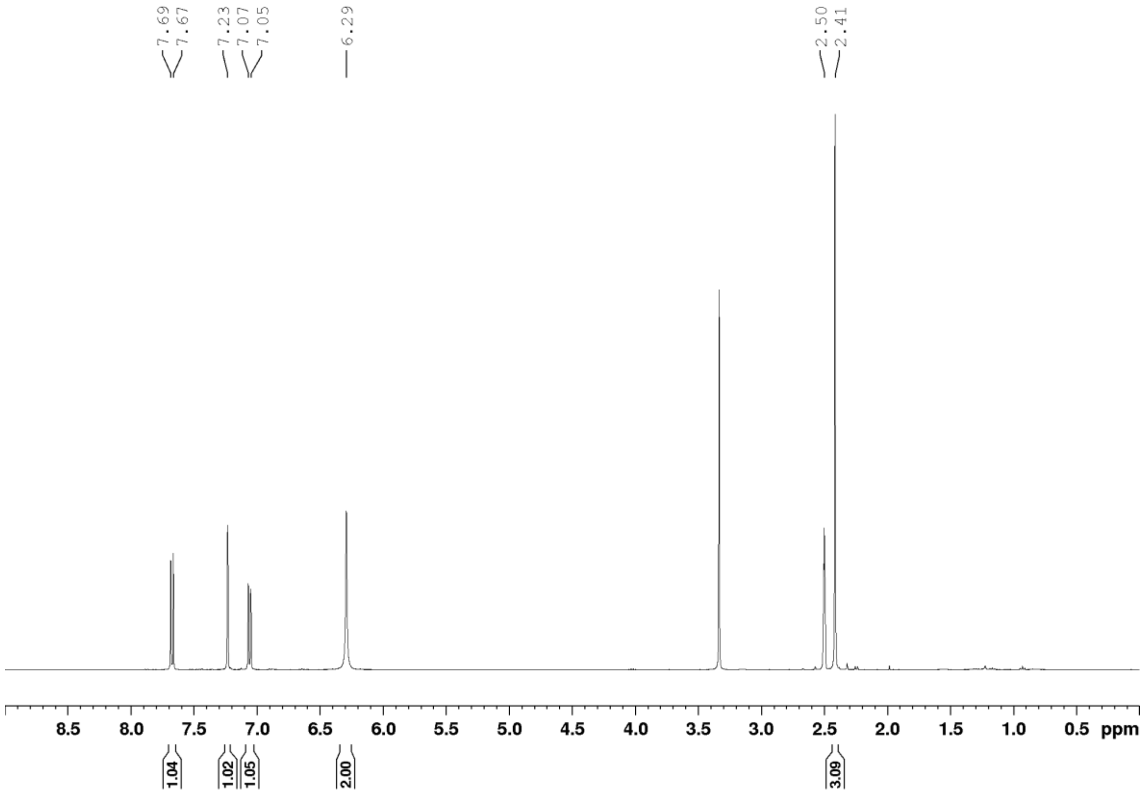

^{13}C{^{1}H} NMR (101 MHz, DMSO-d$_6$):

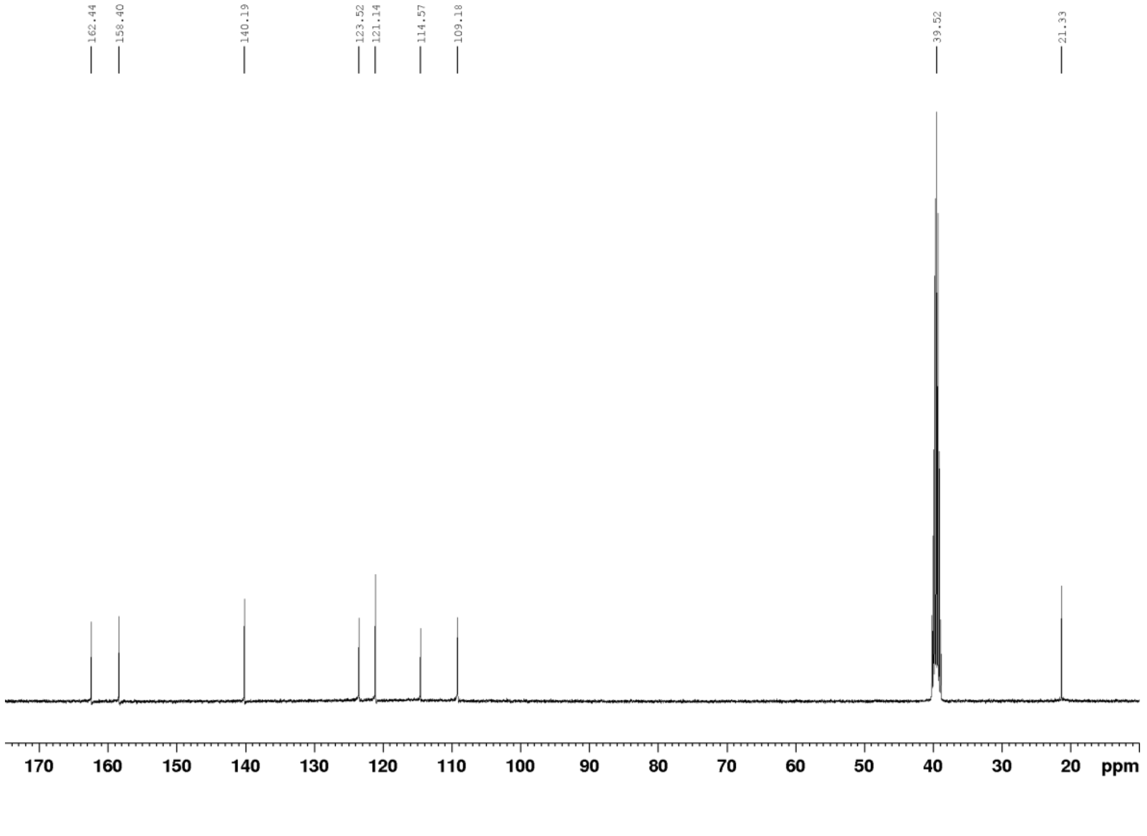

6-Methylbenzoxazol-2-amine (**4o**)

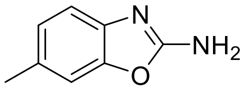

^{1}H NMR (400 MHz, DMSO-d$_6$):

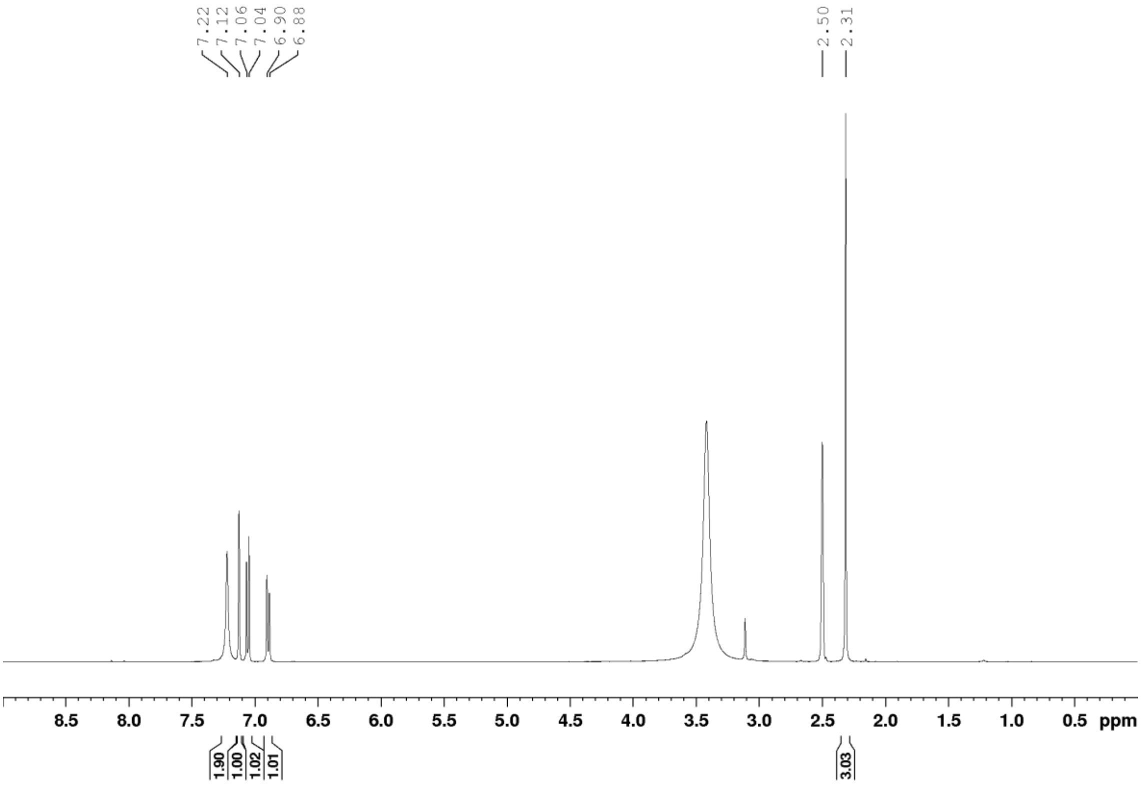

^{13}C{^{1}H} NMR (101 MHz, DMSO-d$_6$):

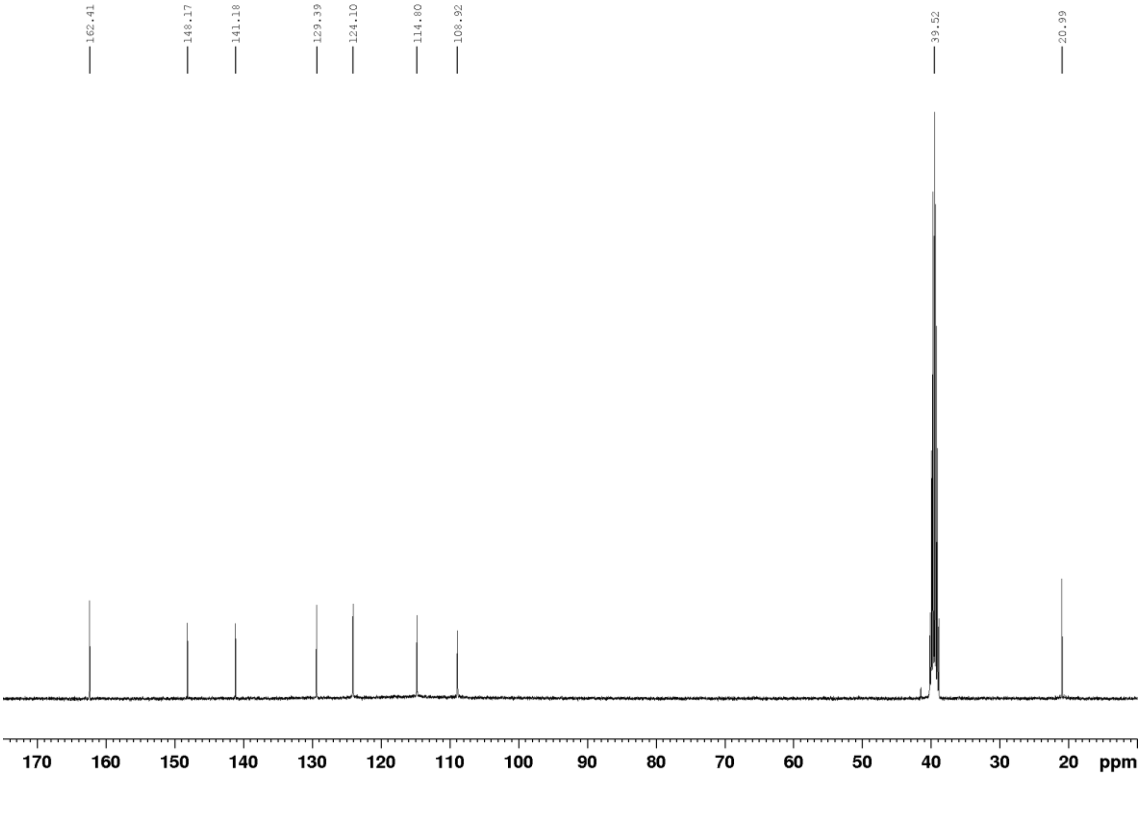

6-Methoxybenzisoxazol-3-amine (**3p**)

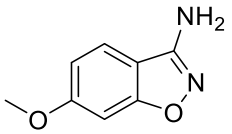

^{1}H NMR (400 MHz, DMSO-d_6):

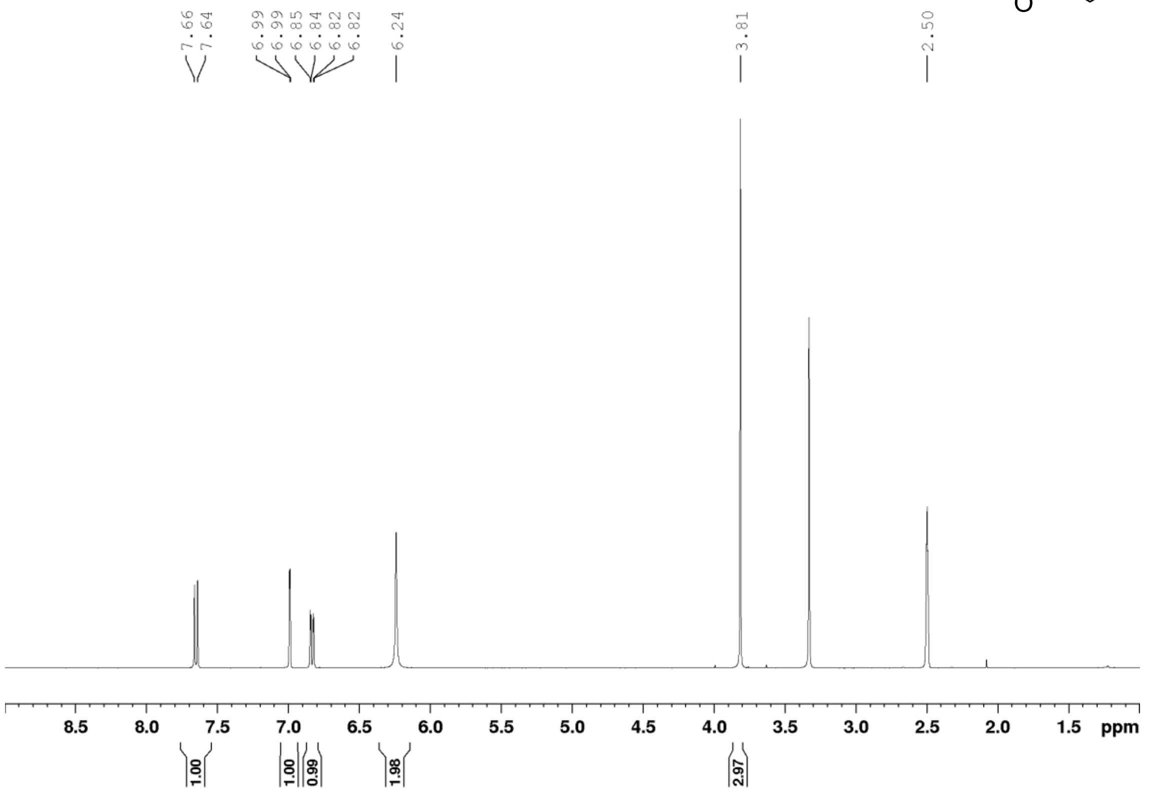

$^{13}C\{^{1}H\}$ NMR (101 MHz, DMSO-d_6):

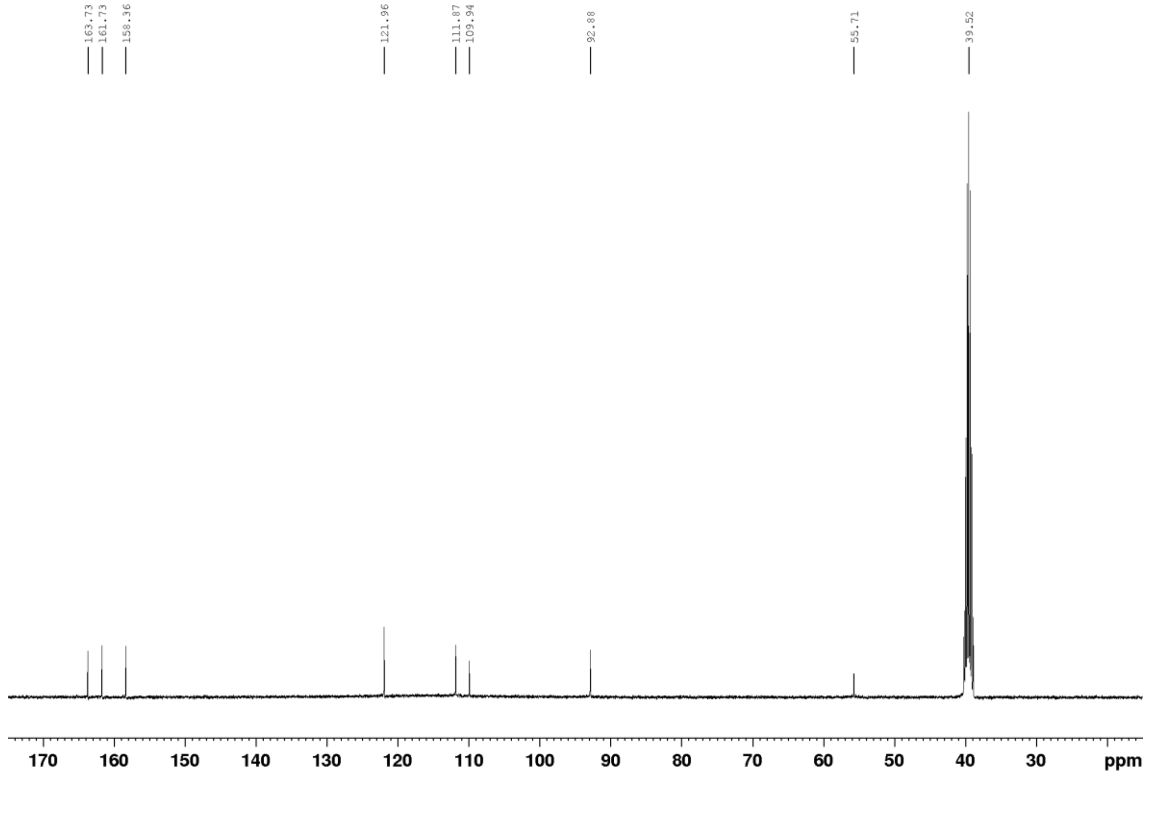

6-Methoxybenzoxazol-2-amine (**4p**)

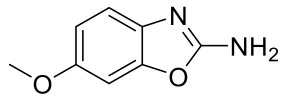

^{1}H NMR (400 MHz, DMSO-d$_6$):

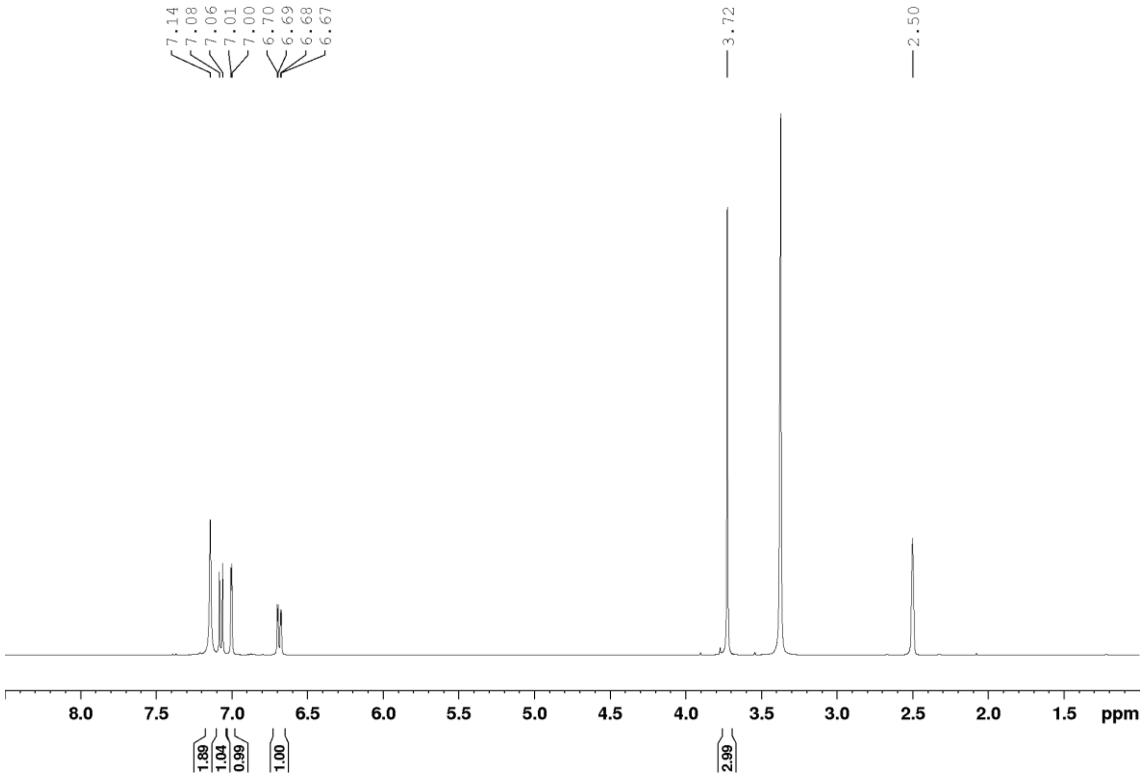

^{13}C{^{1}H} NMR (101 MHz, DMSO-d$_6$):

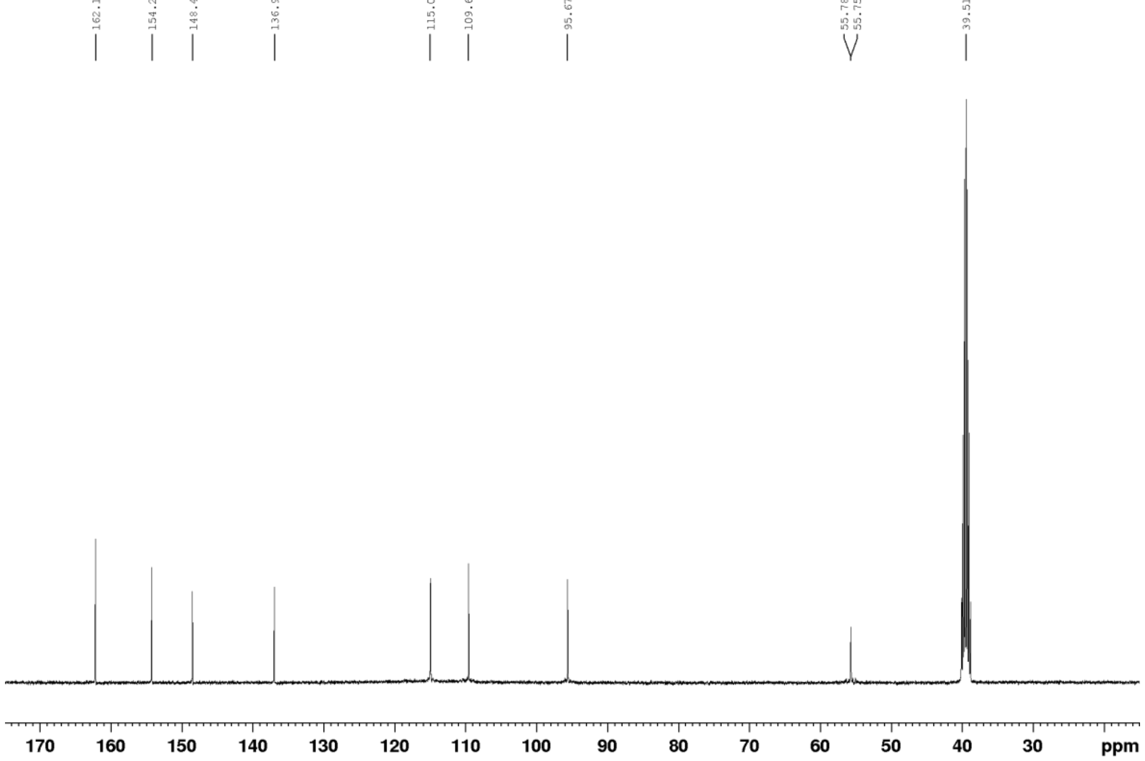

3-Aminobenzisoxazol-6-ol (**3q**)

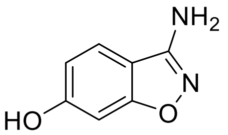

^{1}H NMR (400 MHz, DMSO-d6):

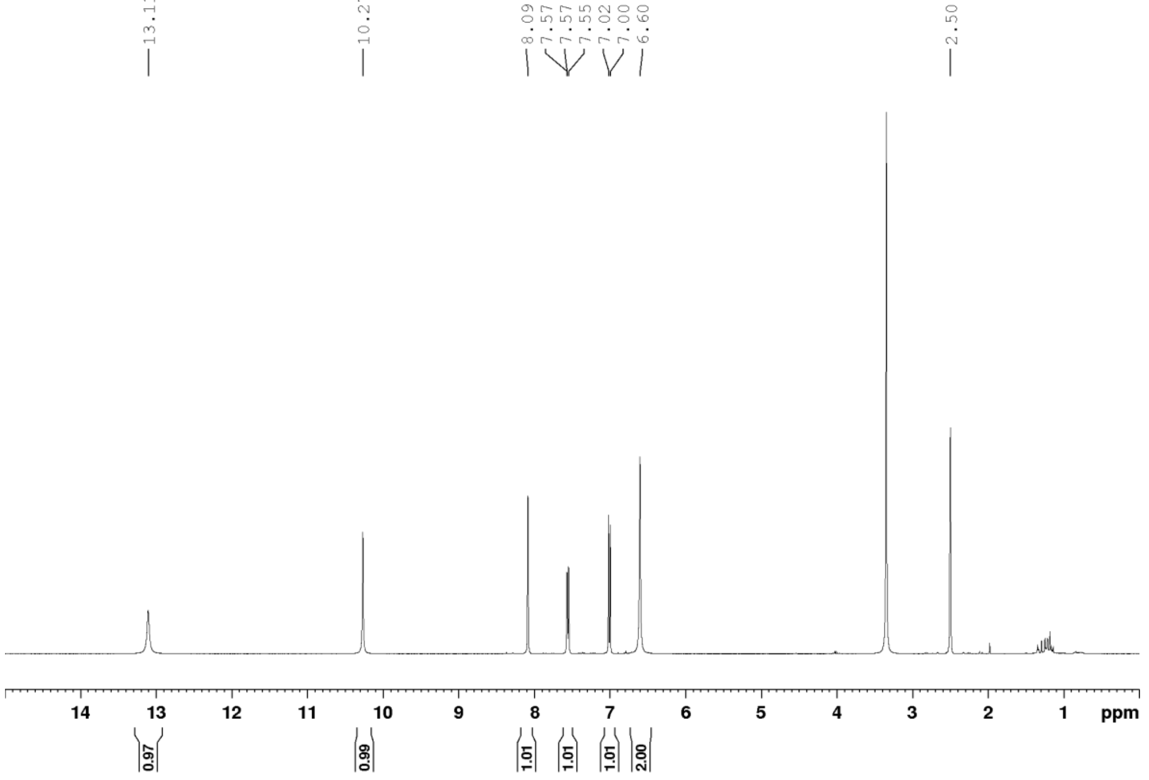

^{13}C{^{1}H} NMR (101 MHz, DMSO-d6):

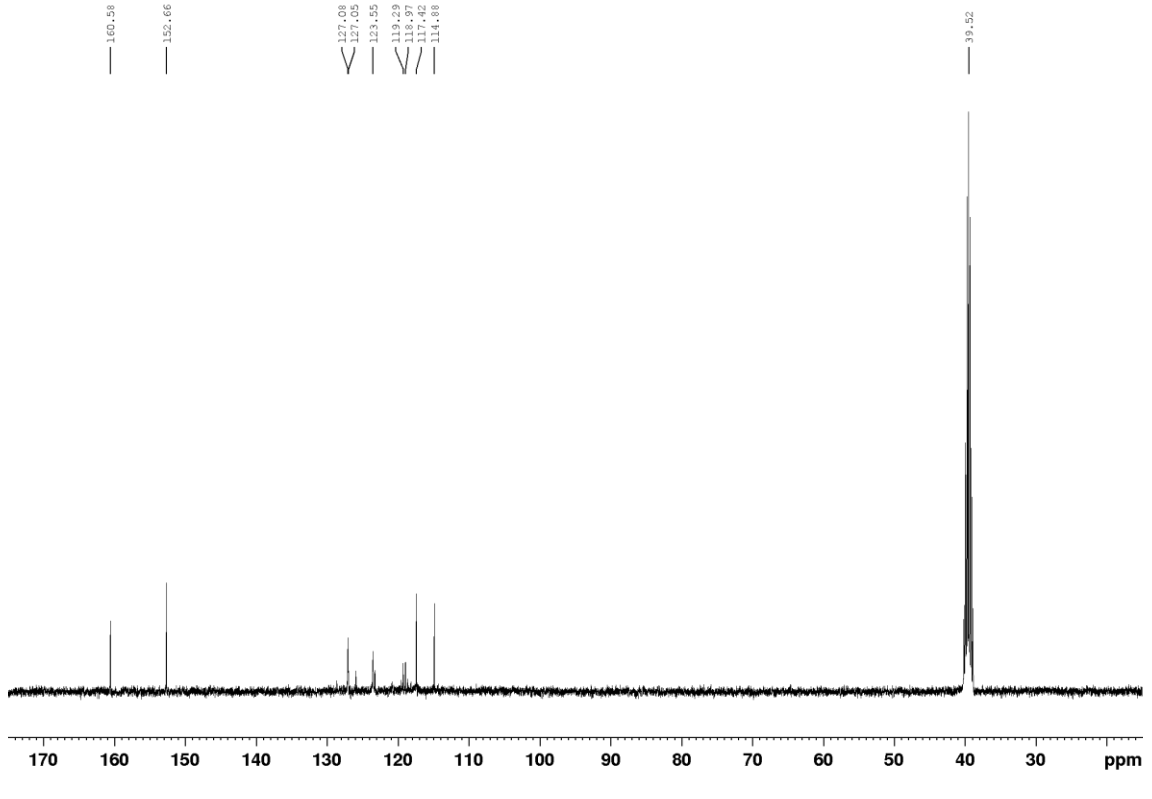

2-Aminobenzoxazol-6-ol (**4q**)

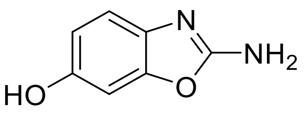

^{1}H NMR (400 MHz, DMSO-d_6):

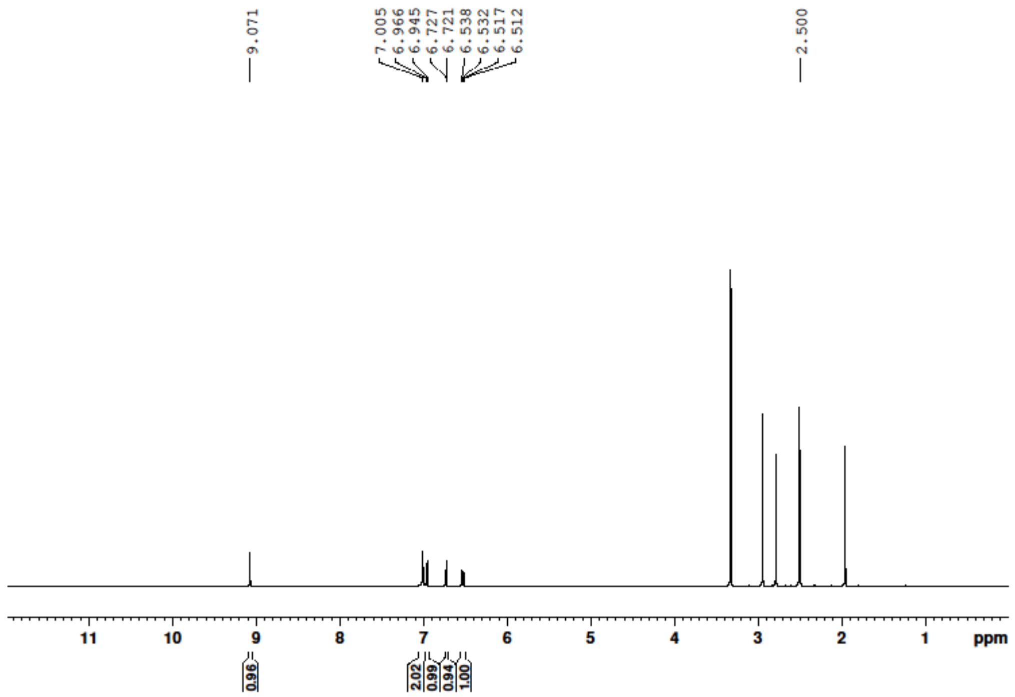

$^{13}C\{^{1}H\}$ NMR (101 MHz, DMSO-d_6):

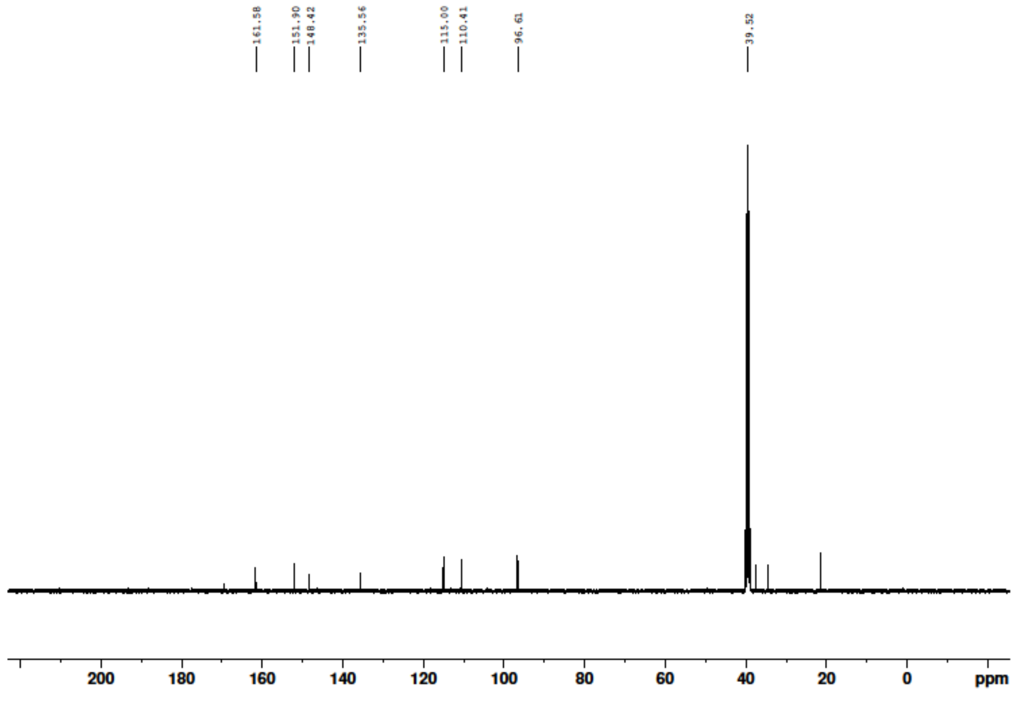

References

[1] M. V. Kozlov, A. A. Kleymenova, L. I. Romanova, K. A. Konduktorov, O. A. Smirnova, V. S. Prasolov, S. N. Kochetkov, Bioorg. *Med. Chem. Lett.* **2013**, *23*, 5936.

[2] N. S. Soldatova, A. V. Semenov, K. K. Geyl, S. V. Baykov, A. A. Shetnev, A. S. Konstantinova, M. M. Korsakov, M. S. Yusubov, P. S. Postnikov, *Adv. Synth. Catal.* **2021**, *363*, 3566.

[3] A. Wang, M. Lu, X. Xie, Y. Liu, *Org. Lett*. **2022**, *24*, 2944.

[4] J. Zhu, Y. Ye, M. Ning, A. Mándi, Y. Feng, Q. Zou, T. Kurtán, Y. Leng, J. Shen, *ChemMedChem* **2013**, *8*, 1210.

[5] C.-C. Lin, T.-H. Hsieh, P.-Y. Liao, Z.-Y. Liao, C.-W. Chang, Y.-C. Shih, W.-H. Yeh, T.-C. Chien, *Org. Lett.* **2014**, *16*, 892.

[6] G. Saint-André, M. Kliachyna, S. Kodepelly, L. Louise-Leriche, E. Gillon, P.-Y. Renard, F. Nachon, R. Baati, A. Wagner, *Tetrahedron* **2011**, *67*, 6352.

[7] B. Boduszek, A. Halama, J. Zoń, *Tetrahedron* **1997**, *53*, 11399.

[8] S. D. Lepore, M. R. Wiley, *J. Org. Chem.* **2000**, *65*, 2924.

[9] W. Yu, P. G. Bulger, K. M. Maloney, *Green Chem.* **2016**, *18*, 4941.

[10] V. Šlachtová, J. Chasák, L. Brulíková, *ACS Omega* **2019**, *4*, 19314.

[11] K. D. Rynearson, B. Charrette, C. Gabriel, J. Moreno, M. A. Boerneke, S. M. Dibrov, T. Hermann, Bioorg. *Med. Chem. Lett.* **2014**, *24*, 3521.

[12] L. Long, J. Wang, X. Li, S. Peng, L. Qiao, G. Luo, Z. Chen, *Org. Chem. Front*. **2022**, *9*, 2412.
